# Active nitrogen mediated selective ruthenium migration on ceria for high pressure ammonia decomposition

**DOI:** 10.1038/s41467-026-74205-7

**Published:** 2026-06-15

**Authors:** Gunjoo Kim, Gahong Kim, Hyunsik Hwang, Eunseong Yoo, Jae-eon Hwang, Jae Won Lee, Hae Ryeong Lee, Keunsoo Kim, Hyangsoo Jeong, Yongmin Kim, Suk Woo Nam, Sungeun Yang, Andreas T. Güntner, Hyunjoo Lee, Keun Hwa Chae, Hyung Chul Ham, Hyuntae Sohn

**Affiliations:** 1 Center for Hydrogen·Fuel Cell Research, Korea Institute of Science and Technology, Seoul, Republic of Korea; 2 Human-Centered Sensing Laboratory, Department of Mechanical and Process Engineering, ETH Zurich, Zurich, Switzerland; 3 Food and Soft Materials Laboratory, Department of Health Sciences & Technology, ETH Zurich, Zurich, Switzerland; 4 Department of Chemistry and Chemical Engineering, Education and Research Center for Smart Energy and Materials, Inha University, Incheon, Republic of Korea; 5 Department of Chemical and Biomolecular Engineering, Korea Advanced Institute of Science and Technology, Daejeon, Republic of Korea; 6 Department of Energy and Environmental Engineering, KIST School, University of Science & Technology (UST), Seoul, Republic of Korea; 7 Center for Hydrogen Energy Materials, Korea Institute of Science and Technology, Seoul, Republic of Korea; 8 Advanced Analysis & Data Center, Korea Institute of Science and Technology, Seoul, Republic of Korea

**Keywords:** Heterogeneous catalysis, Hydrogen storage materials, Catalytic mechanisms

## Abstract

Precise stabilization of atomic structures under reaction environments remains a central challenge in heterogeneous catalysis. Here, we demonstrate that ammonia (NH_3_) serves as a chemically active nitrogen source to derive the selective migration of ruthenium (Ru) atoms onto ceria (CeO_2_) domains, forming a durable atomically dispersed structure. During ammonia decomposition, nitrogen-containing intermediates promote atomic redistribution of Ru and anchor the atoms selectively at CeO_2_, yielding stable Ru-ceria interfaces. The resulting catalyst exhibits high activity in high-pressure ammonia decomposition for hydrogen production, attributed to its lowered activation energy and mitigated hydrogen poisoning. Furthermore, both the catalytic performance and the atomic Ru structure are preserved during long-term high-pressure operation, confirming the exceptional structural stability of the designed configuration. This study establishes active-nitrogen-driven migration as an effective strategy for constructing robust and reaction-friendly catalyst surface.

## Abstract (web summary)

Catalysts often change under working conditions, limiting their stability. This study shows that ammonia can direct ruthenium atoms onto ceria, forming durable active sites for efficient hydrogen production under high-pressure conditions.

## Introduction

Tailoring the atomic-scale interface between metal species and catalyst supports is essential for improving catalytic activity and stability^1–3^. The performance in systems containing mixtures of multiple phases of materials, such as metals, metal oxides, carbides, or nitrides, is strongly dependent on the component that forms the interface with the active metal species^4–8^. However, forming such reaction-specific interfaces remains challenging. Conventional synthesis methods often lead to randomly distributed active metal species across the support, hindering the achievement of selective interfacial structures^9,10^. Addressing this issue typically requires high-temperature treatments, complex precursor systems, or vacuum conditions, all of which increase fabrication complexity and cost^11–14^. Furthermore, even when interfacial structures are carefully tailored, harsh reaction conditions often cause metal migration or restructuring, leading to the loss of the originally designed configurations.

One widely used strategy for guiding the formation of specific metal-support interfaces is to engineer the support surface prior to metal deposition. This bottom-up approach relies on tuning surface properties such as charge distribution, chemical functionality, or defect concentration to create sites that selectively anchor active metal species^15,16^. Thus, metals can be positioned on the catalytically relevant domains of the support instead of being randomly distributed. For example, Li et al. demonstrated that adjusting the solution pH based on the difference in the point of zero charge between silica and cerium oxide allows for the selective adsorption of platinum precursors onto cerium oxide nanoclusters rather than on the surrounding silica surface^17^. This forms well-defined interfaces between platinum and cerium oxide that remain stable even under reducing conditions. Similarly, Kim et al. demonstrated that the introduction of iron into a cerium oxide lattice promotes the formation of oxygen vacancies near the iron sites, which guides rhodium atoms to anchor preferentially in those regions^18^. These examples highlight the advantages of bottom-up strategies for constructing highly active and structurally controlled interfaces through support surface design. However, because these structures are usually optimized under ideal synthesis conditions, they may not always remain intact during actual operation. In reaction environments involving high temperatures or reducing atmospheres, metal atoms can migrate or rearrange, which may alter the original interfacial configuration^19–21^. This suggests the need for alternative approaches that allow interfacial structures to stabilize under real reaction conditions.

In top-down strategies, metal species are initially deposited at random locations, followed by treatments that induce their migration and restructuring. Through such processes, the metal atoms relocate and stabilize into new, well-defined configurations under controlled conditions^22–25^. Wei et al. applied ultrahigh temperatures (~1000 °C) in an inert atmosphere to drive metal migration^26^. Several studies have demonstrated that specific chemicals can promote this transformation. For instance, Jones et al. used oxygen as a chemical mediator to direct atomic restructuring^27^. Hydrothermal treatment with water enabled temperature-dependent control of rhodium dispersion between atomic and clustered forms, while CO treatment induces mild reduction that strengthens palladium-support interactions^28,29^. PH_3_ treatment was also applied to transform noble metal nanoparticles into thermally stabilized metal single atoms on g-C_3_N_4_^30^.

Similarly, ammonia has been explored as an effective medium for inducing atomic dispersion. Qu et al. treated Cu foam and ZIF-8 with ammonia at 900 °C to mobilize Cu as Cu(NH_3_)_x_ and deposited on ZIF-8 as a single atom species^31^. Also, thermal annealing of the material with dicyandiamide (DCD) can induce a similar effect, as DCD is decomposed into NH_3_, leading to re-dispersion of metal particles into atomic structures^32,33^. While these approaches effectively enable atomic dispersion during catalyst synthesis, the restructuring processes are generally conducted as pretreatment or fabrication steps and are not directly coupled with the catalytic reaction environment. Extending such chemically mediated migration strategies into systems where the reactant itself continuously generates active intermediates during operation may provide a more directly aligned approach for constructing and stabilizing reaction-compatible metal-support interfaces under working conditions.

In this study, we integrated key elements of both bottom-up and top-down strategies to design a catalyst whose interfacial structure can be established and maintained under reaction conditions. Ceria was selected as the oxide support component due to its well-known redox flexibility (Ce^3+^/Ce^4+^), ability to form oxygen vacancies, and to have strong metal-support interactions that can stabilize dispersed metal species. However, the intrinsic advantages are not often fully realized when bulk ceria is used directly, as its redox properties and defect structure are strongly influenced by particle size and crystallinity^34,35^. One effective approach to modulate these properties is to control ceria domain size and structural order, which directly affect surface reducibility and vacancy concentration. To achieve this, we employed a high-surface-area carbon substrate as a dispersive framework for ceria domains. By controlling the annealing temperature, crystallinity, domain size, and surface defect density of ceria were tuned, enabling optimization of its surface redox characteristics. Ruthenium was deposited on the optimized support, and ammonia served as a chemically active nitrogen source to induce atomic-scale restructuring. Unlike conventional top-down approaches, reactive nitrogen intermediates generated during ammonia decomposition drove the selective migration of Ru toward CeO_2_ domains under reaction conditions. This reaction-coupled restructuring not only enabled redistribution but also stabilized a catalytically favorable Ru–CeO_2_ interface. In contrast, hydrogen treatment produced randomly dispersed Ru nanoparticles. The resulting Ru–CeO_2_ configuration suppressed hydrogen poisoning and improved compatibility with ammonia decomposition, leading to high hydrogen production under high-pressure conditions while maintaining structural stability after 100 h of continuous operation.

## Results

### Selective migration of Ru atoms onto CeO_2_ into an atomically dispersed structure

CeO_2_-C materials were prepared to obtain CeO_2_ domains with optimal surface redox properties. An amorphous carbon template with a high surface area was coated with CeO_2_ to control the domain size of CeO_2_. Carbon was impregnated with cerium precursors. The samples were annealed under N_2_ at 300, 550, and 700 °C, and are denoted as CeO_2_-C_300, CeO_2_-C_550, and CeO_2_-C_700, respectively. The crystallinity and domain size of CeO_2_ varied with the annealing temperature and were examined using X-ray diffraction (XRD) and transmission electron microscopy (TEM) (Supplementary Figs. 1 and 2). Lower annealing temperatures resulted in smaller crystalline domains. CeO_2_-C_300 exhibited less crystalline and smaller-sized CeO_2_, whereas CeO_2_-C_700 exhibited larger and more defined particles. Ce *3d* and O *1s* X-ray photoelectron spectroscopy (XPS) spectra confirmed that the smaller domains contained a higher concentration of surface defects (Supplementary Fig. 3). The fractions of Ce^3+^ and O_ads_ increased with a decreasing domain size, indicating the formation of more oxygen vacancies and undercoordinated sites. The reducibility of the surface oxygen was evaluated using H_2_-temperature-programmed reduction (H_2_-TPR, Supplementary Fig. 4). The peak onset temperature indicated surface oxygen activation, and the peak area was used to estimate the amount of reduced oxygen. CeO_2_-C_550 exhibited the lowest onset temperature and largest reduction peak, indicating the highest surface reducibility. CeO_2_-C_550 contained more active oxygen species than CeO_2_-C_300 and CeO_2_-C_700, leading to the selection of CeO_2_-C_550 as the optimal support. All subsequent ruthenium catalysts were synthesized and analyzed using CeO_2_-C_550. Ruthenium was impregnated into CeO_2_-C_550 and treated with N_2_. The resulting material was denoted as Ru/CeO_2_-C_N_2_. This catalyst was further treated at 500 °C for 1 h in either 100% NH_3_ or 50% H_2_/Ar and denoted as Ru/CeO_2_-C_NH_3_ and Ru/CeO_2_-C_H_2_, respectively. XRD analyses of the Ru/CeO_2_-C-based catalysts confirmed that CeO_2_ remained dispersed on the carbon support without significant aggregation, regardless of the thermal treatment process (Supplementary Fig. 5). The ruthenium content in the Ru/CeO_2_-C-based catalysts was 1.6 wt%, as determined using ICP-OES, and all the samples exhibited high surface areas >280 m^2^/g (Supplementary Table 1). The Ru/CeO_2_ and Ru/C catalysts had Ru contents of 1.8 and 1.7 wt%, with surface areas of 31.0 and 816.3 m^2^/g, respectively.

**Fig. 1:**
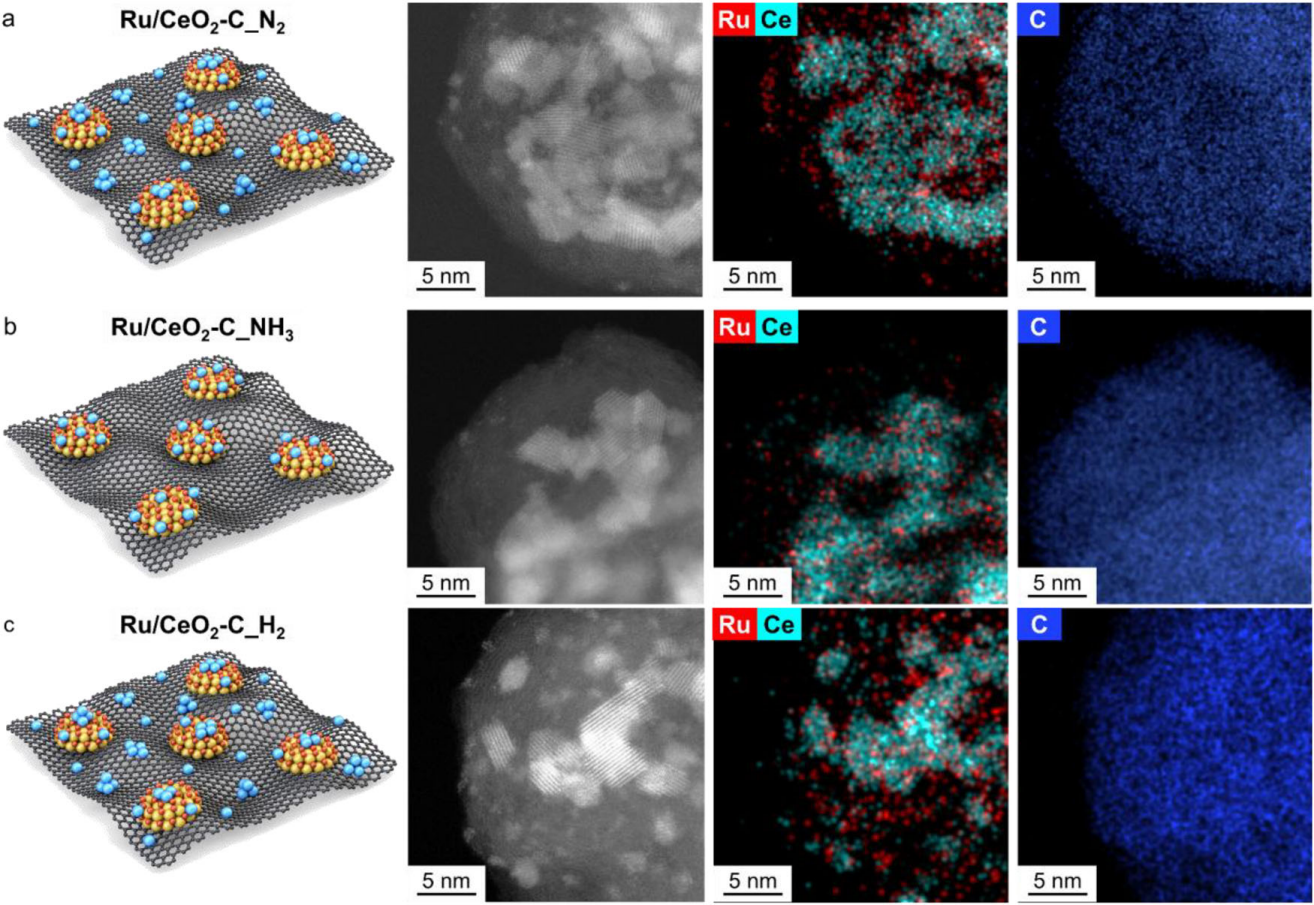
Tuning Ru-support interactions through gas-phase treatments. UHR-TEM and corresponding multi-EDS mapping images for **a** Ru/CeO_2_-C_N_2_, **b** Ru/CeO_2_-C_NH_3_, and **c** Ru/CeO_2_-C_H_2_.

The spatial distribution and local structure of ruthenium were analyzed using ultrahigh-resolution double Cs-corrected TEM (UHR-TEM) with multichannel energy-dispersive X-ray spectroscopy (multi-EDS). The ruthenium species in Ru/CeO_2_-C_N_2_ were randomly distributed over both the CeO_2_ and carbon domains. They existed either in an atomically dispersed form or as nanoparticles (Fig. 1a and Supplementary Fig. 6). After the NH_3_ treatment, most of the ruthenium located on the carbon disappeared, and ruthenium selectively migrated toward the CeO_2_ domains. Only a trace amount of Ru was still detected on the carbon regions together with Ce signals, although the corresponding intensities were significantly lower than those observed on ceria domains (Fig. 1b and Supplementary Fig. 7). In contrast, the H_2_ treatment formed randomly located Ru nanoparticles on both carbon and CeO_2_, with mixed sizes (Fig. 1c and Supplementary Fig. 8). Supplementary Fig. 9 shows additional TEM images. To further examine the structure of Ru species on CeO_2_, UHR-TEM images were taken at higher magnification (Supplementary Fig. 10). The CeO_2_ lattice was clearly resolved, and no Ru nanoparticles or sub-nanometer clusters were observed. Instead, some isolated bright features were detected along with the ceria domains, consistent with atomically dispersed Ru species. These results suggest that NH_3_ treatment enables selective migration with atomic dispersion of ruthenium onto CeO_2_, whereas H_2_ treatment leads to aggregation into small clusters or nanoparticles at random locations. The deposition of ruthenium on CeO_2_ and carbon and treatment with NH_3_ caused it to exist as nanoparticles (Supplementary Fig. 11).

**Fig. 2:**
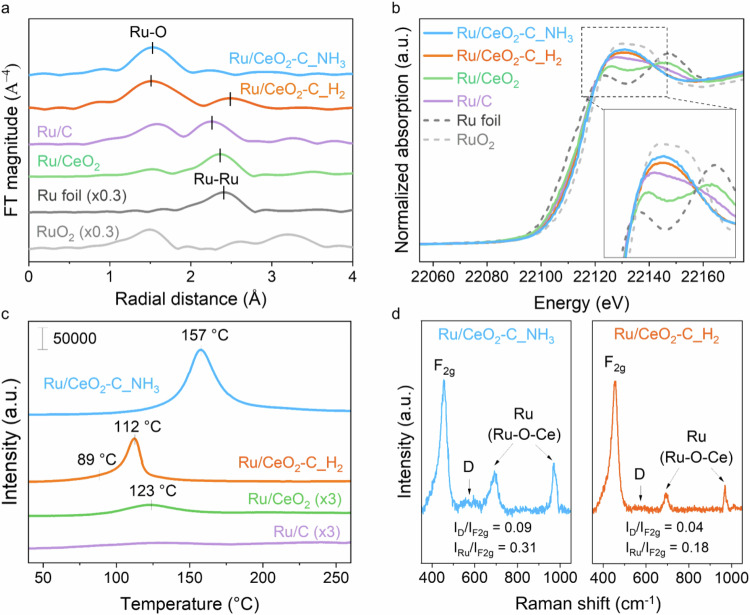
Spectroscopic characterization evidencing Ru–O–Ce interactions. **a** EXAFS and **b** XANES Ru K-edge results. **c** H_2_-TPR results for the ruthenium catalysts. **d** Raman spectra for Ru/CeO_2_-C_NH_3_ and Ru/CeO_2_-C_H_2_.

X-ray absorption fine structure (EXAFS) results confirmed the atomic feature of Ru/CeO_2_-C_NH_3_ (Fig. 2a). Notably, because the scattering paths of Ru–O, Ru–N, and Ru–C are difficult to distinguish owing to their similar bond lengths and backscattering amplitudes, all such first-shell coordinations will hereafter be referred to as Ru–O^36,37^. Ru/CeO_2_-C_NH_3_ exhibited a distinct Ru–O coordination peak at approximately 1.6 Å, while no Ru–Ru peak was observed near 2.4 Å, indicating that ruthenium existed in an atomically dispersed state on the CeO_2_ surface. In contrast, Ru/CeO_2_-C_H_2_, Ru/CeO_2_, and Ru/C exhibited both Ru–O and Ru–Ru coordination peaks, indicating surface Ru nanoparticles. Fitting results revealed Ru–Ru coordination numbers of 0.3 and 1.7 for Ru/CeO_2_-C_NH_3_ and Ru/CeO_2_-C_H_2_, respectively (Supplementary Fig. 12 and Supplementary Table 2). The electronic state of ruthenium was examined using X-ray absorption near-edge structure (XANES) analyses (Fig. 2b). Ru/CeO_2_-C_NH_3_ exhibited a slightly higher white-line intensity than Ru/CeO_2_-C_H_2_, and both exhibited higher intensities than Ru foil, Ru/CeO_2_, and Ru/C.

In addition to the XAS results, H_2_-TPR analyses were performed to gain deeper insight into the surface reducibility of the catalysts and their metal-support interactions (Fig. 2c). Ru/CeO_2_ exhibited a small reduction peak at 123 °C, while Ru/C exhibited no reduction peak. Ru/CeO_2_-C_NH_3_ exhibited a single broad reduction peak at 157 °C, whereas Ru/CeO_2_-C_H_2_ exhibited two distinct peaks at 89 and 112 °C. The higher reduction temperature of Ru/CeO_2_-C_NH_3_ indicates that Ru forms strong interactions with CeO_2_, likely as Ru–O–Ce species. In contrast, the peaks at lower temperatures for Ru/CeO_2_-C_H_2_ suggest the presence of weakly bound ruthenium nanoparticles or clusters on CeO_2_. Notably, Ru/CeO_2_-C_NH_3_ also exhibited a larger reduction peak area, indicating a greater amount of reducible oxygen species. This feature suggests the presence of a higher density of redox-active interfaces in the catalyst, which are associated with surface defect concentration. Such an increase in reducible oxygen is expected to enhance redox cycling at the Ru–CeO_2_ interface, potentially facilitating hydrogen adsorption-desorption dynamics and mitigating hydrogen accumulation on Ru sites. To further probe the oxygen-related surface properties, cryo-TPO was conducted (Supplementary Fig. 13). Both catalysts exhibited oxygen uptake starting from −95 °C, indicating that the Ru surface can be readily oxidized even at very low temperatures. Notably, Ru/CeO_2_-C_NH_3_ showed a larger oxygen uptake with an additional low-temperature shoulder feature compared to Ru/CeO_2_-C_H_2_, suggesting a higher density of oxygen-taking Ru species. This result is consistent with the formation of strongly interacting and highly dispersed Ru species on CeO_2_, leading to a more oxygen-rich interfacial environment. Overall, the combined H_2_-TPR and cryo-TPO results support that Ru/CeO_2_-C_NH_3_ possesses a higher density of redox-active interfacial oxygen species.

Enhanced interaction with Ru and CeO_2_ was also confirmed with Raman spectra (Fig. 2d). The peak at 465 cm^−1^ in the Raman spectra indicates an octahedrally symmetrical vibration mode of CeO_2_ (F_2g_), and that at 600 cm^−1^ indicates a defect-induced mode (D). Furthermore, the peaks observed at 690 and 971 cm^−1^ were attributed to Ru–O–Ce (Ru) interactions^38–41^. Those peaks were not observed in the Raman spectrum of CeO_2_-C (Supplementary Fig. 14). The intensity ratios of these peaks (*I*_D_/*I*_F2g_ and *I*_Ru_/*I*_F2g_) were estimated to compare the surface defect concentration and interaction between Ru and CeO_2_. The surface defect concentration was higher for Ru/CeO_2_-C_NH_3_, as also confirmed by the Ce *3d* and O *1s* XPS profiles (Supplementary Fig. 15). *I*_Ru_/*I*_F2g_ was also higher for Ru/CeO_2_-C_NH_3_ than for Ru/CeO_2_-C_H_2_, indicating that NH_3_-treated Ru/CeO_2_-C exhibited more interaction between ruthenium and CeO_2_. Collectively, these results indicate that NH_3_ treatment may induce atomic dispersion of ruthenium on the CeO_2_ surface, leading to the formation of Ru–O–Ce interactions.

### Driving force of Ru migration during NH_3_ treatment

**Fig. 3:**
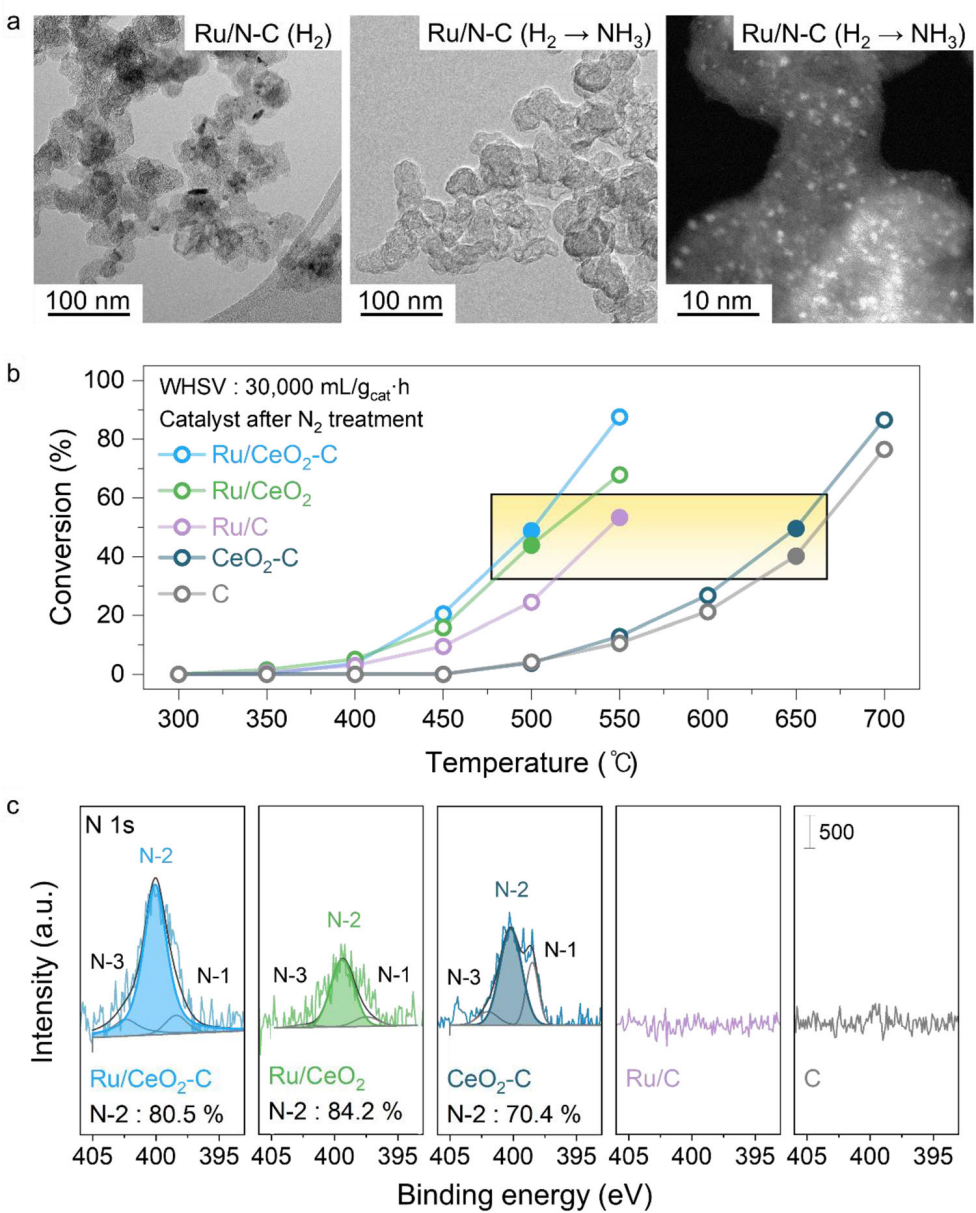
Controlled experiments probing the role of reaction intermediates. **a** TEM images of Ru/N-doped carbon (N–C) after H_2_ reduction (left) and after a subsequent NH_3_ treatment (middle), along with the corresponding HAADF-STEM image (right). **b** NH_3_ decomposition results on the ruthenium base catalysts and bare supports pretreated in N_2_. NH_3_ decomposition was performed at a gas flow of 100% NH_3_ with a WHSV of 30,000 mL/g_cat_·h. **c** N *1s* XPS spectra of the NH_3_-treated catalysts and supports.

To identify the driving force behind ruthenium migration during NH_3_ treatment, the influence of the active intermediate species generated during NH_3_ decomposition was examined. Nitrogen-doped carbon (N–C) was first synthesized and deposited with a target amount of 2 wt% Ru. Nitrogen incorporation into the carbon matrix of N–C was confirmed using N *1s* XPS (Supplementary Fig. 16). The resulting material was then reduced under a 10% H_2_/Ar gas condition at 500 °C for 1 h to evaluate the effect of H and surface-doped N species. Hydrogen can transform the catalyst structure by inducing active metal mobility or surface defect formation, and surface N-doped structures can form stoichiometrically unstable environments that serve as anchoring sites^42,43^. The TEM images of the resulting Ru/N–C (H_2_) revealed highly aggregated ruthenium nanoparticles (Fig. 3a, left), suggesting that the surface-doped nitrogen and hydrogen-derived active intermediates were insufficient to disperse ruthenium. To assess the effect of nitrogen-containing intermediates, the H_2_-treated Ru/N–C sample was further exposed to NH_3_ at 500 °C for 1 h. NH_3_ treatment caused the previously aggregated ruthenium particles to disappear (Fig. 3a, middle), and high-angle annular dark-field scanning transmission electron microscope (HAADF-STEM) analyses confirmed that the ruthenium species were redistributed into smaller particles (Fig. 3a, right). These results indicate that the active-nitrogen-containing species generated from NH_3_ decomposition acted as the key driving force for ruthenium mobilization.

As ruthenium migration is likely driven by its interaction with nitrogen-based intermediates, it was hypothesized that the stabilization sites of these nitrogen species would correlate with the anchoring sites of the mobilized ruthenium. To test this hypothesis, various ruthenium catalysts and their corresponding supports were treated under similar conditions with comparable NH_3_ decomposition rates (Fig. 3b). Blank test result using the quartz reactor is shown in Supplementary Fig. 17. N *1s* XPS measurements were conducted to determine the nitrogen incorporation after treatment. As shown in Fig. 3c, nitrogen doping was observed only in the samples containing CeO_2_ (Ru/CeO_2_-C, Ru/CeO_2_, and CeO_2_-C), whereas no significant nitrogen signals were detected for Ru/C or the bare carbon support. The N *1s* spectrum was deconvoluted into three components N-1 (398.3 eV), N-2 (400.1 eV), and N-3 (402.3 eV). The N-1 peak is assigned to substitutional nitrogen (i.e., N replacing lattice oxygen) and/or surface NH species, the N-2 peak to interstitial nitrogen species and/or NH_2_ species, and the N-3 peak to chemisorbed NH_3_, NH_4_^+^ species, or oxidized nitrogen species (e.g., NO_x_)^44–48^. The largest N *1s* XPS peak appeared at 400.1 eV, suggesting that nitrogen-containing species preferentially incorporated as an interstitial structure, or *NH_2_ species adsorbed on CeO_2_ domains, thereby guiding the migration and anchoring of ruthenium onto CeO_2_ surfaces. Notably, this behavior is distinct from that observed for ruthenium supported on nitrogen-doped carbon, in which nitrogen species predominantly exist as substitutes rather than interstitial nitrogen (Supplementary Fig. 16).

To further resolve the temporal evolution of this process, time-resolved UHR-TEM and N *1s* XPS analyses were conducted. Ru/CeO_2_-C_N_2_ was exposed to NH_3_ at 500 °C for varying durations (1, 5, 10, 20, and 40 min), followed by rapid quenching (Supplementary Fig. 18). At early stages (1 and 5 min), Ru species remained broadly distributed, including on carbon domains. A pronounced redistribution occurred between 5 and 10 min, where Ru signals on carbon decreased and became increasingly localized on CeO_2_ domains. With prolonged treatment, Ru species were predominantly associated with CeO_2_, indicating structural stabilization. Consistently, time-resolved N *1s* XPS results showed that nitrogen species were not detected at early stages but emerged from ~10 min onward, coinciding with the onset of Ru migration (Supplementary Fig. 19). Collectively, these findings demonstrate that nitrogen-containing intermediates generated during NH_3_ decomposition not only mobilize ruthenium, but also direct its relocation to the CeO_2_ domains, thereby promoting the formation of Ru–CeO_2_ interfaces.

To clarify the nature of nitrogen species, N K-edge soft X-ray absorption measurements were performed. As a reference, N-doped CeO_2_ was synthesized via urea thermolysis under N_2_, which is known to have dominant interstitial N species^45^. Although N-doped CeO_2_ exhibited a similar N *1s* XPS peak to Ru/CeO_2_-C_NH_3_, its N K-edge spectrum showed a σ^*^-dominant feature with negligible π^*^ contribution (Supplementary Fig. 20a). In contrast, Ru/CeO_2_-C_NH_3_ showed a markedly different N K-edge spectrum, characterized by a pronounced π^*^ feature at 402.2 eV and a broader, less defined σ^*^ region (Supplementary Fig. 20b)^49–51^. This indicates that, although the N *1s* XPS results are similar, the nitrogen species in Ru/CeO_2_-C_NH_3_ are not equivalent to interstitial nitrogen, but instead exist in more electronically hybridized environments, likely surface chemisorbed *NH_2_ on CeO_2_ domains.

To further examine the evolution of nitrogen species under reaction conditions, the catalyst after 100 h of ammonia decomposition was analyzed. While the N *1s* XPS remains centered at 400.1 eV, the N K-edge spectrum showed a clear change, with decreased π^*^ intensity at 402.2 eV and an increased intensity at 399.8 eV (Supplementary Fig. 20c). Although this change primarily occurred within the π^*^ region rather than forming a fully σ^*^-dominant spectrum, the overall spectral shape shifted toward that of the interstitial-N-dominant reference material. These results suggest that nitrogen species are initially formed as chemisorbed *NH_2_, but undergo partial evolution toward a more stabilized, lattice-associated interstitial configuration during reaction.

Finally, density functional theory calculations were performed to elucidate the role of nitrogen-containing intermediates in both the mobilization of Ru species and their subsequent stabilization on CeO_2_ surfaces. The adsorption energies and the energy required to eject a single ruthenium atom from the Ru(0001) surface in the presence of the adsorbed *NH_3_, *NH_2_, *NH, and *H species were calculated (Supplementary Fig. 21 and Supplementary Table 3). Among these, *NH_2_ was the most effective at promoting ruthenium detachment, corresponding to the highest upshift in the ruthenium d-band center. The resulting Ru–NH_2_ species were then stabilized on both carbon and CeO_2_ (111) supercells, with adsorption showing a preference for CeO_2_ over carbon (Supplementary Fig. 22). Also, we constructed models of N-doped CeO_2_ (111) with nitrogen located at interstitial sites and evaluated their stability. Among the considered configurations, surface interstitial (case 1 in Supplementary Fig. 23a) was found to be the most stable (nitrogen binding energy of −2.45 eV), compared to subsurface configurations. Based on this configuration, the adsorption energies of Ru on pristine CeO_2_ and N-doped CeO_2_ surfaces were calculated. The adsorption energy of Ru was significantly enhanced from −4.97 eV on pristine CeO_2_ to −6.58 eV on N-doped CeO_2_, indicating a stronger interaction between Ru and the N-doped surface.

To explore the applicability of NH_3_ treatment beyond ruthenium, Pt/CeO_2_-C and Rh/CeO_2_-C catalysts were prepared and treated under conditions that provided comparable NH_3_ decomposition rates to those of Ru/CeO_2_-C_N_2_ (Supplementary Fig. 24a). UHR-TEM and multi-EDS mapping revealed that both platinum and rhodium particles were located near the CeO_2_ domains (Supplementary Fig. 24b, c). These observations suggest that NH_3_ treatment facilitates the formation of metal-CeO_2_ interfaces for ruthenium and for other noble metals.

### Hydrogen production by high-pressure NH_3_ decomposition

Ammonia decomposition at high pressure represents a key step in realizing efficient hydrogen production and storage, yet catalyst stability remains a major challenge under such harsh conditions. Ammonia is a promising hydrogen carrier because of its high hydrogen content, carbon-free composition, and ease of liquefied storage and transport^52–54^. Hydrogen extraction from ammonia typically requires thermal decomposition above 450 °C^55–57^. Especially, high-pressure operation is favored for practical applications. Although ammonia decomposition is thermodynamically favored at low pressure, high-pressure operation is advantageous from a process perspective as it enables direct integration with downstream hydrogen purification systems such as pressure swing adsorption (PSA)^58–60^. In low-pressure systems, an additional compression step is required prior to purification, which is energy-intensive and increases both capital and operational costs^61,62^. Hydrogen compression from 1 to 10 bar typically requires on the order of ~1 kWh/kg_H2_, corresponding to a non-negligible energy penalty and additional cost^63^. Operating the reactor under elevated pressure eliminates the need for recompression, thereby improving overall process efficiency despite a modest thermal penalty associated with slightly higher reaction temperatures.

However, under the harsh reaction conditions of high-pressure NH_3_ decomposition, catalyst degradation is accelerated, including metal sintering and interfacial breakdown. To overcome these issues, a catalyst capable of maintaining structural integrity under reactive NH_3_ conditions is required. Our NH_3_-treated Ru/CeO_2_-C catalyst may fulfill this criterion, as the high-temperature NH_3_ treatment drives selective relocation of Ru into atomically dispersed configurations stabilized on CeO_2_ surfaces. Therefore, this catalyst was employed for high-pressure NH_3_ decomposition to evaluate its performance and durability.

**Fig. 4:**
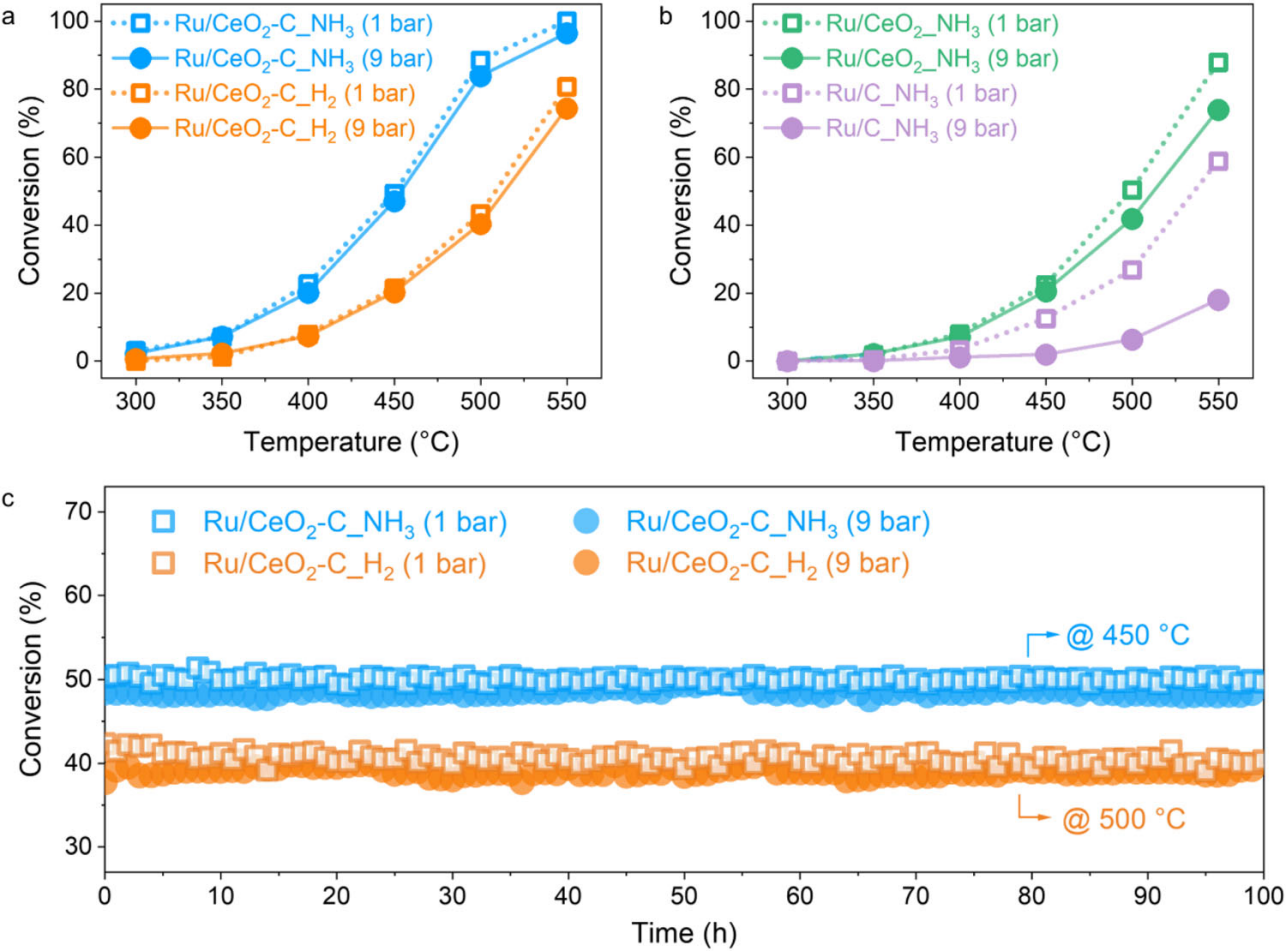
Catalytic performance in ammonia decomposition reactions. NH_3_ conversion results for **a** Ru/CeO_2_-C_NH_3_, Ru/CeO_2_-C_H_2_, **b** Ru/CeO_2_, and Ru/C at 1 and 9 bar. **c** Long-term stability test of Ru/CeO_2_-C_NH_3_ and Ru/CeO_2_-C_H_2_ for 100 h at 1 and 9 bar. The long-term reaction was conducted at 450 and 500 °C for Ru/CeO_2_-C_NH_3_ and Ru/CeO_2_-C_H_2_, respectively. NH_3_ decomposition was conducted using 100% NH_3_ with a WHSV of 30,000 mL/g_cat_·h.

When ammonia decomposition was monitored under both 1 and 9 bar, Ru/CeO_2_-C_NH_3_ exhibited significantly higher activity than Ru/CeO_2_-C_H_2_, Ru/CeO_2_, and Ru/C (Fig. 4a, b). Varying the NH_3_ pretreatment temperature from 300 to 700 °C revealed that the optimal activity was achieved at 500 °C (Supplementary Fig. 25). Moreover, as compared to previously reported values, the hydrogen production rate achieved using our catalyst under high-pressure conditions highly exceeded those reported for NH_3_ decomposition at similar pressures (Supplementary Table 4).

To understand the origin of this enhanced performance, kinetic and mechanistic analyses were conducted in both ambient and pressurized conditions, based on the activation energies and reaction orders. The activation energy determined from the Arrhenius plots showed that Ru/CeO_2_-C_NH_3_ exhibited a lower activation barrier of 64.0 kJ/mol (1 bar) and 59.9 kJ/mol (9 bar), as compared to 103.9 kJ/mol (1 bar) and 98.8 kJ/mol (9 bar) for Ru/CeO_2_-C_H_2_. This indicates that NH_3_ activation occurred more easily over the Ru/CeO_2_-C_NH_3_ (Supplementary Fig. 26a, b). The reaction order was determined by varying the partial pressures of H_2_ and NH_3_. The order with respect to NH_3_ was 0.95 (1 bar) and 0.96 (9 bar) for the Ru/CeO_2_-C_NH_3_, 0.91 (1 bar) and 0.94 (9 bar) for the Ru/CeO_2_-C_H_2_, suggesting that NH_3_ activation proceeded through a similar kinetic regime for both catalysts (Supplementary Fig. 26c, d). In contrast, the reaction order with respect to hydrogen was −0.54 (1 bar), and −0.64 (9 bar) for Ru/CeO_2_-C_H_2_, −0.23 (1 bar), and −0.32 (9 bar) for Ru/CeO_2_-C_NH_3_, confirming that the Ru/CeO_2_-C_NH_3_ had less hydrogen poisoning on Ru sites (Supplementary Fig. 26e, f). These results indicate that the selectively formed Ru–CeO_2_ interfaces via NH_3_ treatment facilitated efficient NH_3_ activation while mitigating hydrogen inhibition, thereby accelerating the overall decomposition process. As the differences between ambient and elevated pressures were not pronounced, the consistent activation energy trends and reaction orders confirmed that the intrinsic catalytic behavior is maintained.

The structural stability of the catalysts was assessed through a long-term durability test for 100 h at 1 and 9 bar. Both Ru/CeO_2_-C_NH_3_ and Ru/CeO_2_-C_H_2_ maintained their initial catalytic activities throughout the reactions without noticeable deactivation, indicating their high operational durability under these reaction conditions (Fig. 4c). In addition, the catalytic performance was maintained over three consecutive reaction cycles under high-pressure conditions (Supplementary Fig. 27). The catalysts were investigated after long-term reaction at high pressure to confirm structure stability. The EXAFS analysis of the Ru/CeO_2_-C_NH_3_ after durability test showed that the Ru–O coordination remains high (3.9), while the Ru–Ru coordination remains negligible (0.2), indicating that no significant aggregation of Ru species occurred during reaction. In contrast, the Ru/CeO_2_-C_H_2_ after durability test exhibited a substantially higher Ru–Ru coordination (1.4), consistent with the presence of Ru nanoparticles. These results demonstrated that the atomic Ru structure formed after NH_3_ treatment was preserved even under prolonged high-pressure ammonia decomposition (Supplementary Fig. 28a, b, and Supplementary Table 5). Furthermore, Ru K-edge XANES spectra showed negligible changes before and after reaction, indicating that the oxidation state of Ru was well maintained (Supplementary Fig. 28c). Ru *3p* XPS analysis exhibited slight shift to higher binding energy (~0.4 eV), without any evidence of metallic Ru formation, while preserving the relative difference between catalysts (Supplementary Fig. 28d). In addition, Ce *3d* XPS and Raman analysis confirmed that the ceria structure and the Ru–CeO_2_ interaction remain largely unchanged after reaction (Supplementary Fig. 28e–g). XRD results for the Ru/CeO_2_-C-based catalysts after 100 h of reaction showed that the CeO_2_ domains did not aggregate or sinter during the operation (Supplementary Fig. 28h). Finally, the UHR-TEM and multi-EDS results confirmed that the ruthenium in Ru/CeO_2_-C_NH_3_ remained atomically dispersed on CeO_2_ domains even after the long-term high-pressure reaction (Supplementary Fig. 29a). In contrast, the ruthenium in Ru/CeO_2_-C_H_2_ still existed as nanoparticles randomly distributed across the support without preferential interaction with CeO_2_ (Supplementary Fig. 29b). This structural conservation suggests that ammonia-driven restructuring occurs primarily before the formation of stabilized Ru nanoparticles. Once strong Ru–Ru bonding and anchoring interactions are established, large-scale redistribution becomes kinetically hindered. Consequently, initial NH_3_ treatment directed the formation of energetically stabilized and structurally robust Ru–CeO_2_ interfaces, which preserve atomic dispersion and catalytic performance under high-pressure NH_3_ decomposition.

### Interface-dependent reaction mechanisms for NH_3_ decomposition

To elucidate the effect of the NH_3_ treatment-induced structural rearrangement on catalytic performance, the distribution and stabilization of hydrogen and electrons produced during NH_3_ decomposition were examined. Ruthenium species can reside on both carbon and CeO_2_, and their distribution depends on the pretreatment conditions. When NH_3_ decomposes over ruthenium on carbon, the resulting hydrogen and electrons can reduce carbon, leading to methane (CH_4_) formation with the undesired consumption of electrons and hydrogen. In contrast, when NH_3_ decomposes over ruthenium on CeO_2_, hydrogen is effectively trapped and released from the CeO_2_ support, while electrons are transferred to the carbon support and subsequently supplied to the ruthenium surface when required, thereby increasing the ruthenium electron density.

**Fig. 5:**
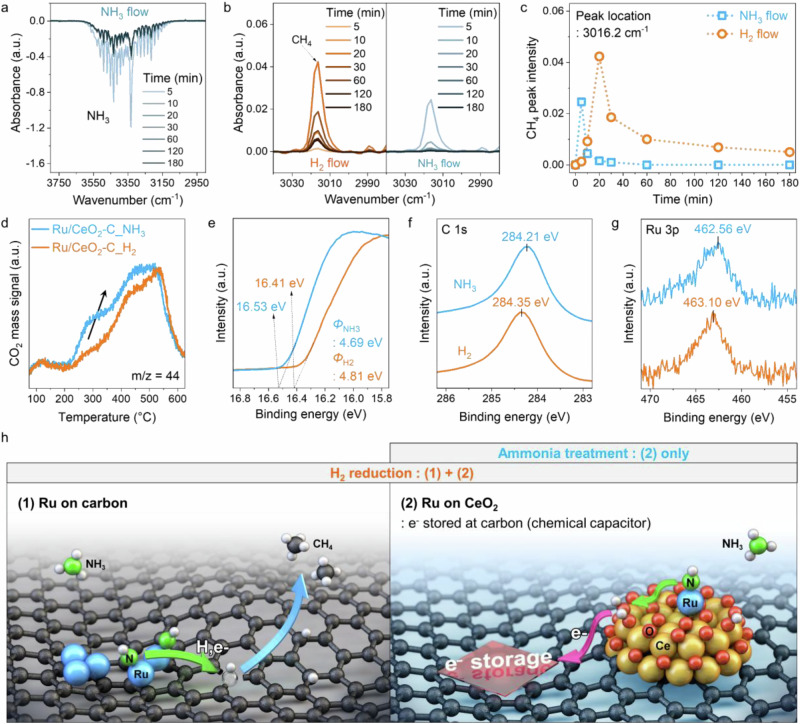
Spatial separation of hydrogen and electron pathways during NH_3_ decomposition. **a** NH_3_ consumption for Ru/CeO_2_-C_N_2_ monitored using FT-IR gas cells at 500 °C for 3 h. **b**, **c** CH_4_ formation observed using FT-IR gas cells for Ru/CeO_2_-C_N_2_ after treatment with 50 % H_2_/Ar (orange) and 100 % NH_3_ (blue) at 500 °C for 3 h. **d** CO_2_-TPD results of Ru/CeO_2_-C_NH_3_ and Ru/CeO_2_-C_H_2_ observed using a mass detector. **e** UPS spectra, **f** C *1s* XPS, and **g** Ru *3p* XPS results for Ru/CeO_2_-C_NH_3_ and Ru/CeO_2_-C_H_2_. **h** Schematic diagram illustrating the hydrogen and electron pathways during NH_3_ decomposition over ruthenium located on the carbon of CeO_2_.

This behavior was first confirmed by CH_4_ detection using Fourier-transform infrared spectroscopy with a gas-cell setup. Treating Ru/CeO_2_-C_N_2_ with pure NH_3_ at 500 °C resulted in rapid consumption of NH_3_ within 10 min, which stabilized for 3 h (Fig. 5a). Simultaneously, CH_4_ was produced, but diminished quickly and disappeared after approximately 20 min, indicating the rapid rearrangement of ruthenium onto the CeO_2_ surface. This CH_4_ formation result was consistent with time-resolved TEM and time-resolved N *1s* XPS results (Supplementary Figs. 18 and 19), which showed that Ru migration occurs within a similar time window accompanied by the formation of nitrogen species. The coincidence of methane formation with the Ru migration onset and nitrogen incorporation suggests a strong correlation between these results. In contrast, the H_2_ treatment at 500 °C on Ru/CeO_2_-C_N_2_ exhibited continuous CH_4_ formation over 3 h with a larger total amount, suggesting the prolonged residence of ruthenium on the carbon support (Fig. 5b, c).

The formation of basic surface sites due to hydrogen trapping on CeO_2_ was supported by the results of temperature-programmed desorption using CO_2_ (CO_2_-TPD). Ru/CeO_2_-C_NH_3_ exhibited a desorption peak that shifted toward higher temperatures with a larger peak signal, indicating enhanced basicity of the surface (Fig. 5d). This agrees with the increase in the Ce^3+^ and O_ads_ ratios determined using XPS (Supplementary Fig. 15). Also, to directly investigate hydrogen adsorption behavior, temperature-programmed desorption using H_2_ (H_2_-TPD) experiments were performed (Supplementary Fig. 30). A low-temperature desorption peak below ~200 °C is attributed to hydrogen desorption from Ru sites, while a broader feature at 300–600 °C corresponds to hydrogen stored on CeO_2_ via H_2_-spillover^64,65^. Notably, Ru/CeO_2_-C_NH_3_ exhibited a pronounced high-temperature desorption peak despite hydrogen adsorption at room temperature, indicating efficient hydrogen spillover from Ru to the CeO_2_ support. This redistribution of hydrogen away from Ru sites reduced hydrogen accumulation and mitigated hydrogen poisoning.

The associated electron transfer and storage on the carbon support were investigated using ultraviolet photoelectron spectroscopy (UPS) and XPS. The UPS results show that Ru/CeO_2_-C_NH_3_ exhibited a lower work function (4.69 eV) than Ru/CeO_2_-C_H_2_ (4.81 eV), indicating an upward shift in the Fermi level and an increased electron density on the surface (Fig. 5e). The C *1s* and Ru *3p* XPS spectra of Ru/CeO_2_-C_NH_3_ also showed redshifts, confirming electron accumulation on the carbon and ruthenium surfaces (Fig. 5f, g). Supplementary Fig. 31 shows additional C *1s* XPS and UPS results for the bare carbon and CeO_2_-C. These results suggest that during NH_3_ decomposition of Ru/CeO_2_-C_NH_3_, hydrogen atoms generated on Ru migrate to the CeO_2_ surface, where they are temporarily trapped and then coupled to form H_2_. Meanwhile, the accompanying electrons are transferred to the carbon support and later supplied back to the Ru sites when needed. Figure 5h schematically illustrates the proposed mechanism. This spatial separation of hydrogen and electron pathways enhances reaction efficiency and suppresses hydrogen poisoning, as evidenced by the reduced hydrogen inhibition observed for Ru/CeO_2_-C_NH_3_ (Supplementary Fig. 26).

## Discussion

NH_3_ treatment on the Ru/CeO_2_-C catalyst induced a selective atomic rearrangement of ruthenium toward CeO_2_ domains, yielding an atomically dispersed, robust Ru structure on the CeO_2_ surface (Ru/CeO_2_-C_NH_3_). This interfacial reconstruction was driven by reactive nitrogen intermediates generated during NH_3_ decomposition, which mediated site-specific migration and anchoring of Ru atoms. Detailed structural analyses using UHR-TEM, multi-EDS, EXAFS, and Raman spectroscopy confirmed that Ru was atomically dispersed and preferentially stabilized on CeO_2_. The Ru/CeO_2_-C_NH_3_ catalyst exhibited efficient catalytic activity and long-term durability in high-pressure NH_3_ decomposition. The enhanced performance can be attributed to the enriched atomic Ru sites on CeO_2_ that promote redox-mediated NH_3_ activation and to the efficient removal of hydrogen through CeO_2_-assisted trapping and recombination, which mitigates hydrogen poisoning. Collectively, these findings demonstrate that the active-nitrogen-driven formation of atomic Ru sites on CeO_2_ surfaces offers a robust and general approach to construct thermodynamically stable and catalytically active structures.

## Methods

### Catalyst synthesis

CeO_2_-coated carbon (CeO_2_-C) with a CeO_2_ content of 63.0 wt% was synthesized by a wet impregnation method. Specifically, 600 mg of Ketjen black carbon (EC 300J) was dispersed in 30 mL of ethanol, and 1.8 g of cerium(III) nitrate hexahydrate (Ce(NO_3_)_3_·6H_2_O, 99.99 %, Kanto Chemical) was dissolved in 20 mL of ethanol. Two solutions were combined in a 250 mL round-bottom flask and stirred vigorously for over 2 h. Ethanol was evaporated using a rotary evaporator under reduced pressure (0.08 MPa) in a 40 °C water bath. The resulting solid was dried overnight at 60 °C in a vacuum oven and then thermally treated under flowing N_2_ in a tube furnace at 300, 550, or 700 °C for 4 h. The resulting samples were designated as CeO_2_-C_300, CeO_2_-C_550, and CeO_2_-C_700, respectively.

Ruthenium was deposited onto CeO_2_-C_550 using a wet impregnation method. Initially, CeO_2_-C_550 was dispersed in ethanol, and an appropriate amount of Ru(III) chloride hydrate (≥99 %, Sigma-Aldrich) for 1.6 wt% was dissolved in 5 mL of ethanol and added dropwise to the CeO_2_-C_550 suspension. This mixture was stirred vigorously for 2 h and heated at 60 °C using a heating mantle until complete evaporation of ethanol. The resulting powder was dried overnight at 60 °C, followed by calcination at 200 °C for 1 h under N_2_ flow. The synthesized catalyst was denoted as Ru/CeO_2_-C_N_2_. Catalysts subsequently treated at 500 °C for 1 h under pure NH_3_ or 50% H_2_/Ar were labeled Ru/CeO_2_-C_NH_3_ and Ru/CeO_2_-C_H_2_, respectively. Using the same method, Ru was also deposited onto commercial CeO_2_ nanopowder (<25 nm, Sigma-Aldrich) followed by NH_3_ treatment at 500 °C for 1 h, and these catalysts were denoted as Ru/CeO_2_. When deposited onto carbon followed by NH_3_ treatment at 550 °C, the catalyst was denoted as Ru/C.

Nitrogen-doped carbon (N–C) was synthesized following a previously reported method^66^. Briefly, 1 g of dicyandiamide (DCD) was dissolved in 50 mL of ethanol under sonication in a round-bottom flask. Then, 500 mg of carbon was added to the mixture along with an additional 20 mL of ethanol. The resulting solution was sonicated for 2 h and stirred overnight at 60 °C until complete solvent removal. The obtained solid was thermally treated at 550 °C for 4 h under Ar flow. Ru was subsequently loaded onto the N–C via wet impregnation using the same procedure described above, followed by calcination at 200 °C for 1 h under N_2_.

Nitrogen-doped CeO_2_ was synthesized following a previously reported method^45^. 200 mg of urea (Sigma-Aldrich) was well mixed with 400 mg of CeO_2_ (Sigma-Aldrich). Then, this powder was thermally treated in N_2_ at 400 °C for 4 h.

### Characterization

The crystal structure of the samples was analyzed using a powder X-ray diffractometer (XRD; D8 ADVANCE, LynxEye). The Brunauer-Emmett-Teller (BET) surface area of the catalysts was determined using a Tristar II 3020 instrument (Micromeritics). X-ray absorption spectroscopy (XAS) at the Ru K-edge was performed at the BM31 setup of Swiss-Norwegian beamlines (SNBL at ESRF) and the 7D beamline of the Pohang Light Source (PLS). The Ru K-edge spectra were calibrated against a Ru foil, and data processing was carried out using the ATHENA and ARTEMIS software packages. N K-edge spectra were obtained in the 10D beamline of Pohang Light Source (PLS).

Structure optimization of Ru in the Ru/CeO_2_-C catalyst was investigated using ultrahigh-resolution double Cs-corrected transmission electron microscopy (UHR-TEM), conducted on a Spectra Ultra TEM (Thermo Fisher Scientific) equipped with multichannel energy-dispersive X-ray spectroscopy (multi-EDS) mapping. Bright-field (BF) images were acquired using a Tecnai F20 G2 microscope (FEI), while high-angle annular dark-field scanning transmission electron microscopy (HAADF-STEM) images were obtained using a Titan TM 80–300 microscope (FEI).

Temperature-programmed reduction using hydrogen (H_2_-TPR) was conducted with a BELCAT M instrument (MicrotracBEL) equipped with a thermal conductivity detector (TCD). Prior to measurement, the catalysts were pretreated under argon at 150 °C for 1 h and then cooled to room temperature. CO_2_ temperature-programmed desorption (CO_2_-TPD) was carried out on a BELCAT II (MicrotracBEL) with a mass spectrometer (BELMASS; MicrotracBEL) as a detector. Catalysts were pretreated under helium at 200 °C for 1 h and then cooled to room temperature. CO_2_ was purged for 30 min, and He was injected to eliminate gaseous CO_2_. The temperature was ramped up by 10 °C, observing CO_2_ emission with a mass spectrometer. Cryo-TPO and H_2_-TPD were conducted with AutoChem III (Micromeritics) equipped with a thermal conductivity detector (TCD). In the case of cryo-TPO, the samples were first mildly reduced at 200 °C under 5% H_2_/Ar, followed by cooling to −95 °C under He. Subsequently, the gas was switched to 5% O_2_/He, and after stabilization, the temperature was increased to 400 °C while monitoring oxygen consumption. For H_2_-TPD, the catalyst was pretreated in flowing Ar at 200 °C and cooled down to 30 °C under Ar. This was followed by hydrogen adsorption at 30 °C using a 4 vol% H_2_/Ar mixture and subsequent Ar purging to remove gas-phase and weakly physisorbed hydrogen. Finally, the temperature was ramped from 30 to 800 °C under Ar.

Raman spectra were obtained using an InVia Raman Microscope (Renishaw). X-ray photoelectron spectroscopy (XPS) was carried out on a Thermo Fisher Scientific Nexsa spectrometer using a monochromatic Al Kα radiation source (1486.6 eV). Ultraviolet photoelectron spectroscopy (UPS) was conducted using a Thermo Fisher Scientific Nexsa spectrometer with a He I photon source (21.22 eV). The work function was calculated from the secondary electron cutoff energy (*E*_cut-off_) in the UPS spectra using Eq. (1).

1 $${{\rm{work}}}\,{{\rm{function}}}={hv}\,({{\rm{He}}}\; {{\rm{I}}}\; {{\rm{photon}}}\; {{\rm{energy}}})-{E}_{{{\rm{cut}}}-{{\rm{off}}}}$$

All XPS measurements were calibrated against a cleaned Au film (Au 4*f*_7/2_ binding energy of 84.0 eV) prior to analysis, and the samples were stored under a nitrogen atmosphere in a glove box immediately after gas treatment to minimize exposure to air.

Gas-phase species were analyzed via Fourier-transform infrared spectroscopy (FT-IR) using an IRTracer-100 instrument (Shimadzu) equipped with a long-path gas cell (PIKE). Ar was introduced to purge the system, and then heated to 500 °C. Then, the feed gas was switched to either 50 % H_2_/Ar or 100% NH_3_. Spectra were collected every 5 min.

### Ammonia decomposition

Ammonia decomposition for hydrogen production was carried out in a fixed-bed quartz reactor with a ½-inch diameter under a flow of pure NH_3_. For high-pressure experiments, a ½-inch SUS reactor was used. The catalyst was placed at the center of the reactor, and the reaction temperature was monitored using a thermocouple positioned directly beneath the catalyst bed. Unless otherwise specified, the catalysts were pretreated using pure ammonia. The gaseous products were analyzed via an online gas chromatograph (GC, Agilent 7890 A) equipped with a thermal conductivity detector (TCD) and a CP-Volamine column (CP7448, Agilent, 60 m × 0.32 mm × 5 μm). Ammonia conversion was calculated using Eq. (2).

2 $${{\rm{NH}}}_{3}\,{{\rm{conversion}}}(\%)=\frac{{[{{\rm{NH}}}_{3}]}_{inlet}-{[{{\rm{NH}}}_{3}]}_{outlet}}{{[{{\rm{NH}}}_{3}]}_{inlet}+{[{{\rm{NH}}}_{3}]}_{outlet}} \times 100$$

[NH_3_]_inlet_ and [NH_3_]_outlet_ refer to NH_3_ concentrations in reactant and product gases measured by GC-TCD. Here, [NH_3_]_outlet_ considers the expansion of the product gas from ammonia decomposition. Therefore, it is equivalent to Eq. (3).

3 $${[{{\rm{NH}}}_{3}]}_{{\rm{outlet}}}=\frac{{n}_{{NH}3,{outlet}}}{{V}_{{NH}3,{outlet}}+{V}_{N2,{outlet}}+{V}_{H2,{outlet}}}$$

${n}_{{{\rm{NH}}}3,{{\rm{outlet}}}}$ denotes the number of moles of ammonia in product gas, and ${V}_{{{\rm{NH}}}3,{{\rm{outlet}}}}$, ${V}_{{{\rm{N}}}2,{{\rm{outlet}}}}$, and ${V}_{{{\rm{H}}}2,{{\rm{outlet}}}}$ are the volumes of unreacted ammonia, produced nitrogen, and hydrogen, respectively.

For the activation barrier, the weight hourly space velocity (WHSV) was maintained at 120,000 mL/g_cat_·h to ensure sufficient NH_3_ feed during the reaction. For reaction order experiments with H_2_ and NH_3_, the weight hourly space velocity (WHSV) was maintained at 60,000 mL/g_cat_·h. Activation barrier measurement under ambient pressure for Ru/CeO_2_-C_NH_3_ was conducted at 420, 430, 440, 450, and 460 °C. At each temperature point, the reaction was allowed to reach equilibrium before the next step. For Ru/CeO_2_-C_H_2_, experiment was performed at 430 °C, 440 °C, 450 °C, 460 °C, and 470 °C. The reaction order experiments were conducted by varying the partial pressures of H_2_ and NH_3_ while maintaining a constant total flow rate. The total flow rate was balanced with Ar to keep the overall space velocity constant at 60,000 mL/g_cat_ · h. For the H_2_ reaction order experiment, Ru/CeO_2_-C_NH_3_ was tested at 450 °C with H_2_ concentrations set to 10%, 20%, 30%, and 40%. In the case of Ru/CeO_2_-C_H_2_, an experiment was conducted at 500 °C. NH_3_ reaction order experiments were carried out by adjusting the NH_3_ concentration to 100%, 60%, 40%, and 20%. Ru/CeO_2_-C_NH_3_ was tested at 400 °C, while Ru/CeO_2_-C_H_2_ was evaluated at 500 °C. In case of high-pressure experiments, H_2_ reaction order of Ru/CeO_2_-C_NH_3_ was tested at 400 °C, meanwhile Ru/CeO_2_-C_H_2_, experiment was conducted at 450 °C. NH_3_ reaction order experiments under high pressure with Ru/CeO_2_-C_NH_3_ were tested at 400 °C, while Ru/CeO_2_-C_H_2_ was evaluated at 450 °C.

### Computational detail

Calculations were performed based on spin-polarized density functional theory (DFT) by employing the Vienna Ab initio Simulation Package (VASP, version 5.4.4)^67^. To approximate the exchange-correlation interaction, the generalized gradient approximation (GGA) of Perdew–Burke–Ernzerhof was employed^68–70^. The projector-augmented wave (PAW) method was also employed to describe the interaction between ion cores and valence electrons^71^. An energy cutoff of 500 eV was applied for the expansion of plane wave functions. For the Brillouin zone integration, we used Monkhorst-Pack mesh of 3 × 3 × 1 k-points in structural optimization, and increased 7 × 7 × 1 k-points in a partial density of states (PDOS) analysis^72^. Ionic relaxation was conducted until the residual force was less than 5 × 10^−2 ^eV/Å and the energy convergence criterion was 1 × 10^−4^ eV. Also, especially for ceria, the empirical Hubbard, U, parameter (U = 5.0 eV) was provided to describe localized electrons of the 4f orbital in Ce of CeO_2_^73^.

To investigate the migration of Ru atoms induced by NH_x_, a Ru (0001) surface was constructed, which is generally known as the most stable surface^74^. Its surface was represented by a 4 × 4 supercell slab model with 5 atomic layers, where the top three layers were fully relaxed, and the bottom two layers were fixed. Also, a Graphene and CeO_2_ (111) facet slab model was constructed to investigate the thermodynamic favorability of Ru migration induced by NH_x_. In this model, a 5 × 5 graphene supercell and a 3 × 3 CeO_2_ (111) slab were constructed to form a heterointerface. The graphene layer was modeled as a planar honeycomb structure, while the CeO_2_ surface was cleaved along the (111) facet, which is the most thermodynamically stable surface of fluorite-type ceria. To minimize interfacial strain and lattice mismatch, the lateral lattice constants of the two components were carefully matched, with the graphene and CeO_2_ supercells having lattice parameters of 12.337 Å and 11.660 Å, respectively. This supercell configuration enables a stable interface suitable for further surface interaction and electronic structure analysis.

The adsorption energies of NH_x_ species on Ru(0001) and the ejection energies of Ru-NH_x_ complexes were calculated using Eqs. (4) and (5). Here, Ru^*^_NHx_ refers to the system in which an NH_x_ species is adsorbed on the bare Ru surface, E_mol_ denotes the energy of the corresponding molecule in the gas phase, and Ru_vacancy_ represents the Ru slab with one Ru atom removed from the surface. Calculations were performed for NH_3_, NH_2_, NH, N, and H adsorbed on the Ru(0001) surface.

4 $${{{\rm{E}}}}_{{{\rm{ads}}}}={{\rm{E}}}\left({{\rm{R}}}{{{\rm{u}}}}^{*}{{{\rm{NH}}}}_{{{\rm{x}}}}\right)-{{\rm{E}}}\left({{\rm{Ru}}}\right)-{{{\rm{E}}}}_{{{\rm{mol}}}}({{{\rm{NH}}}}_{{{\rm{x}}}})$$

5 $${{{\rm{E}}}}_{{{\rm{eject}}}}={{\rm{E}}}\left({{\rm{R}}}{{{\rm{u}}}}_{{{\rm{vacancy}}}}\right)+{{{\rm{E}}}}_{{{\rm{mol}}}}\left({{\rm{Ru}}}-{{{\rm{NH}}}}_{{{\rm{x}}}}\right)-{{\rm{E}}}({{\rm{R}}}{{{\rm{NH}}}}_{{{\rm{x}}}})$$

To further validate this, partial density of states (PDOS) calculations for Ru atoms directly bonded to each adsorbate were performed. From these results, the average d-band center energy of the Ru atoms was determined for each adsorption configuration. Additionally, to investigate where the NH_2_-induced Ru species is preferentially stabilized, the formation energies of Ru–NH_2_ adatoms migrating from the Ru surface to each support were calculated with Eqs. (6) and (7). Specifically, ${E}_{{adatom},{Ru}-{NH}2}^{({Ru}\to {Gr})}$ was calculated, which denotes the energy difference between the initial state of NH_2_ adsorbed on the Ru surface and the final state of Ru–NH_2_ adsorbed on Graphene. Similarly, ${E}_{{adatom},{Ru}-{NH}2}^{({Ru}\to {CeO}2)}$ was also calculated. which denoted the energy difference between the initial state of NH_2_ adsorbed on Ru surface and the final state of Ru–NH_2_ adsorbed on CeO_2_ (111). Each value is defined as follows. Gr^*^_Ru-NH2_ and CeO_2_^*^_Ru-NH2_ refer to Ru–NH_2_ species adsorbed on graphene and CeO_2_, respectively. The specific definitions of each energy term are provided in the following equations.

6 $${E}_{{adatom},{Ru}-{NH}2}^{({Ru}\to {Gr})}={{\rm{E}}}\left({{\rm{G}}}{{{{\rm{r}}}}^{*}}_{{{\rm{Ru}}}-{{\rm{NH}}}2}\right)+{{\rm{E}}}\left({{\rm{R}}}{{{\rm{u}}}}_{{{\rm{vacancy}}}}\right)-({{\rm{E}}}({{\rm{Gr}}})+{{\rm{E}}}({{\rm{R}}}{{{{\rm{u}}}}^{*}}_{{{\rm{NH}}}2}))$$

7 $${E}_{{adatom},{Ru}-{NH}2}^{({Ru}\to {CeO}2)}={{\rm{E}}}\left({{{{\rm{C}}}{{\rm{e}}}{{{\rm{O}}}}_{2}}^{*}}_{{{\rm{Ru}}}-{{\rm{NH}}}2}\right)+{{\rm{E}}}\left({{\rm{R}}}{{{\rm{u}}}}_{{{\rm{vacancy}}}}\right)-\left({{\rm{E}}}\left({{\rm{Ce}}}{{{\rm{O}}}}_{2}\right)\right)+{{\rm{E}}}\left({{\rm{R}}}{{{{\rm{u}}}}^{*}}_{{{\rm{NH}}}2}\left.\right)\right)$$

To model the stable N-doped CeO_2_(111) system, and to reflect the experimental observation of interstitial nitrogen, three potential sites of N were simulated. Case 1 represented a nitrogen atom located at the Ce-Ce-O site on the surface of the CeO_2_(111) slab, case 2 represented a nitrogen atom located at the Ce-O-Ce-O site, and case 3 represented a nitrogen atom located at the Ce-Ce-O site on the subsurface of the CeO_2_(111) slab. Adsorption energy of the Ru atom on the surface was calculated on the N-doped CeO_2_(111) and pristine CeO_2_(111), according to Eq. (8) shown below.

8 $${{{\rm{E}}}}_{{{\rm{ads}}}}\left({{\rm{Ru}}}\right)={{\rm{E}}}\left({{\rm{Ru}}}/{{\rm{substrate}}}\right)-{{\rm{E}}}\left({{\rm{substrate}}}\right)-{{\rm{E}}}({{\rm{Ru}}})$$

Where, E(Ru/substrate), E(substrate), and E(Ru) denote the total energy of a Ru-adsorbed substrate (pristine or N-doped CeO_2_ (111) slab), the total energy of a bare substrate, and the total energy of an isolated single Ru atom in a vacuum system.

## Supplementary information

Supplementary Information

Transparent Peer Review file

## Data Availability

The authors declare that the data supporting the findings of this study are available within the paper and its Supplementary Information files. Should any raw data files be needed in another format, they are available from the corresponding author upon request.
