# Supplementary material for: Active nitrogen mediated selective ruthenium migration on ceria for high pressure ammonia decomposition
Source: Nat Commun. 2026 Jun 15;17:7541. doi: 10.1038/s41467-026-74205-7 (PMC13408114; doi:10.1038/s41467-026-74205-7)
Supplement: Supplementary file 1 — Supplementary Information [file 41467_2026_74205_MOESM1_ESM.pdf]

Supplementary Information for

Active Nitrogen Mediated Selective Ruthenium Migration on Ceria for High Pressure Ammonia Decomposition

Gunjoo Kim[a, b‡], Gahong Kim[a, c‡], Hyunsik Hwang[a], Eunseong Yoo[d], Jae-eon Hwang[e], Jae Won Lee[e], Hae Ryeong Lee[e], Keunsoo Kim[a,f], Hyangsoo Jeong[a,f], Yongmin Kim[a], Suk Woo Nam[a], Sungeun Yang[g], Andreas T. Güntner[b], Hyunjoo Lee[e], Keun Hwa Chae[h], Hyung Chul Ham[d], and Hyuntae Sohn[a,f,*].

[a] Center for Hydrogen·Fuel Cell Research, Korea Institute of Science and Technology, Seoul 02792, Republic of Korea

[b] Human-Centered Sensing Laboratory, Department of Mechanical and Process Engineering, ETH Zurich, CH 8092 Zurich, Switzerland

[c] Food and Soft Materials Laboratory, Department of Health Sciences & Technology, ETH Zurich, 8092 Zurich, Switzerland

[d] Department of Chemistry and Chemical Engineering, Education and Research Center for Smart Energy and Materials, Inha University, Incheon 22212, Republic of Korea

[e] Department of Chemical and Biomolecular Engineering, Korea Advanced Institute of Science and Technology, Daejeon 34141, Republic of Korea

[f] Department of Energy and Environmental Engineering, KIST School, University of Science & Technology (UST), Seoul, 02792, Republic of Korea

[g] Center for Hydrogen Energy Materials, Korea Institute of Science and Technology, Seoul 02792, Republic of Korea

[h] Advanced Analysis & Data Center, Korea Institute of Science and Technology, Seoul 02792, Republic of Korea

[‡]These authors contributed equally to this work

Table of Contents :

Supplementary Figures (Figure S1 ~ S31)
Supplementary Table (Table S1 ~ S5)
Supplementary References

Supplementary Figures :

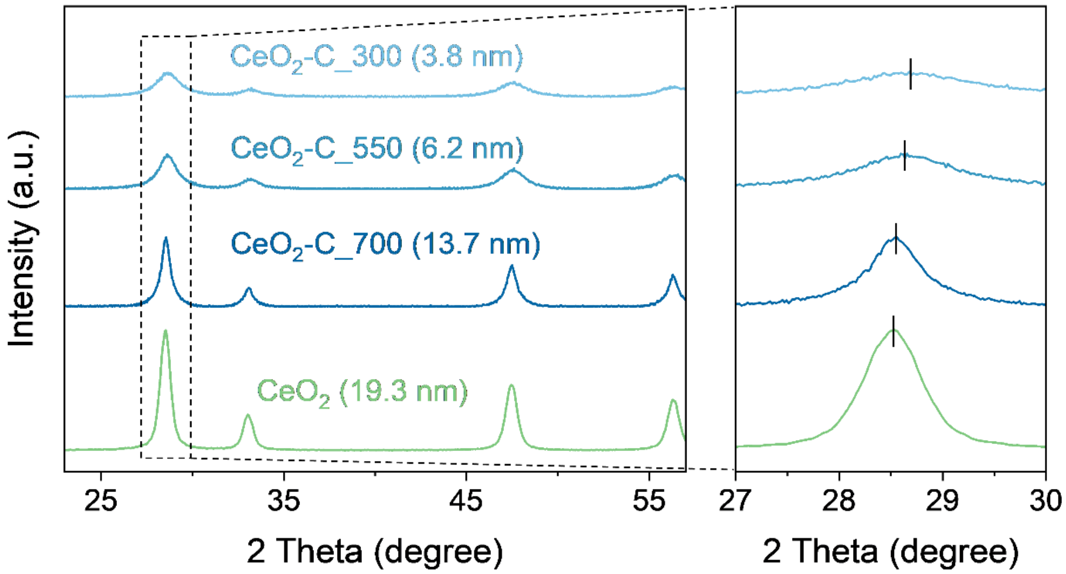

Supplementary Fig. 1 X-ray diffraction (XRD) patterns of CeO_2-coated carbon supports (CeO_2-C) after different annealing temperatures. CeO_2-C_300, CeO_2-C_550, and CeO_2-C_700 denotes supports after annealing under N_2 at 300 °C, 550 °C, and 700 °C respectively.

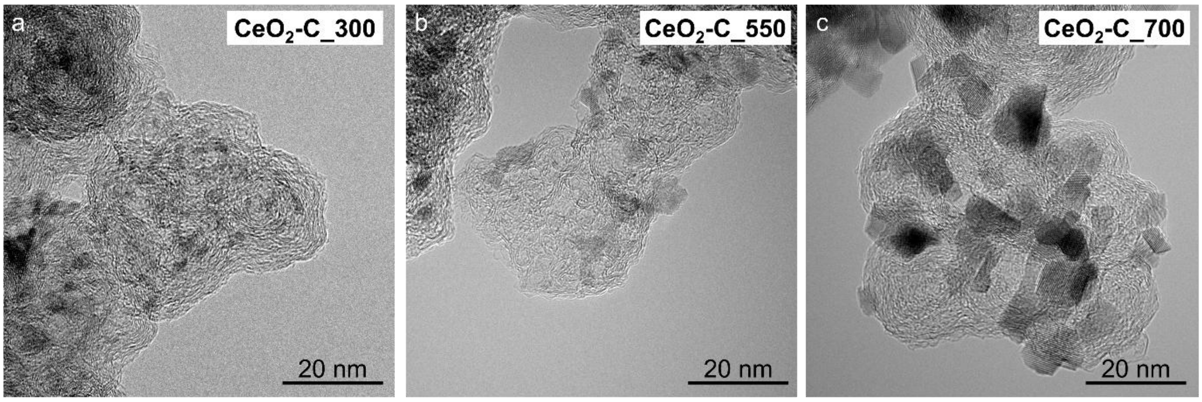

Supplementary Fig. 2 TEM images of CeO_2-C_300, CeO_2-C_550, and CeO_2-C_700.

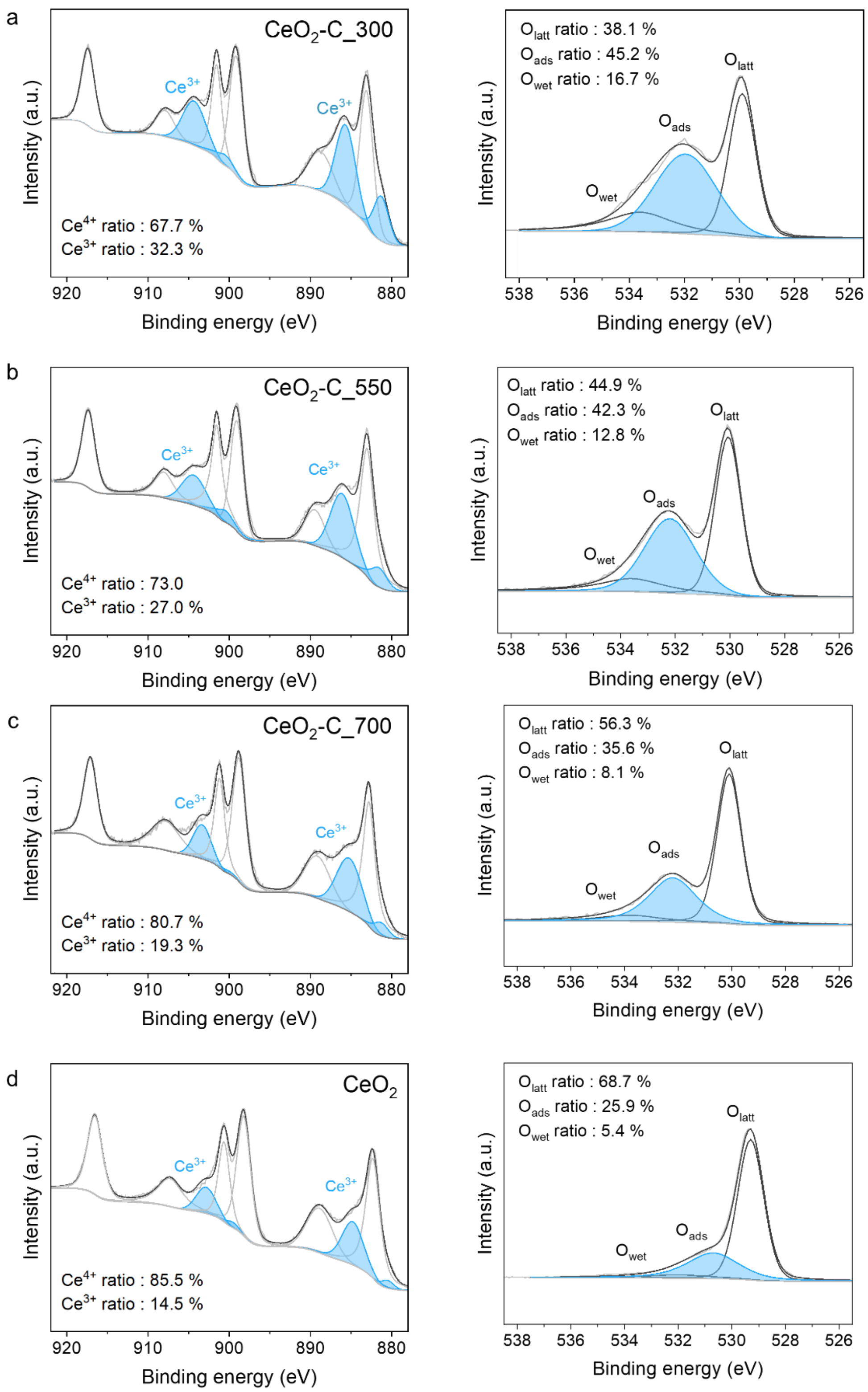

Supplementary Fig. 3 Ce 3d (left column) and O 1s (right column) XPS results of (a) CeO_2-C_300, (b) CeO_2-C_550, (c) CeO_2-C_700, and (d) CeO_2.

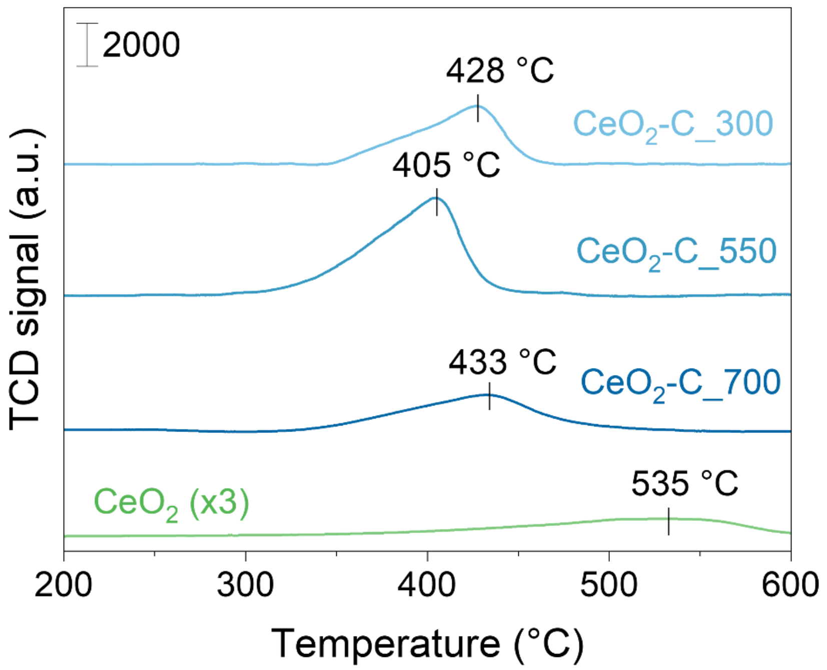

Supplementary Fig. 4 H_2-TPR results for CeO_2-C_300, CeO_2-C_550, CeO_2-C_700, and CeO_2.

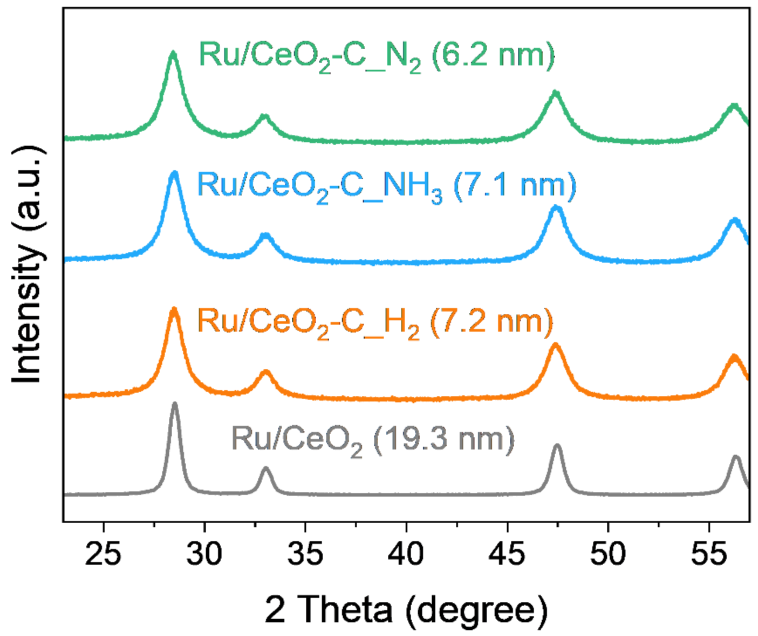

Supplementary Fig. 5 XRD results for Ru/CeO_2-C_N_2, Ru/CeO_2-C_NH_3, Ru/CeO_2-C_H_2, and Ru/CeO_2.

Supplementary Table 1. ICP-OES and BET surface area results of the catalysts.

Sample	Ru content (wt %)	CeO_2 content (wt %)	Surface area (m^2/g)
Ru/CeO_2-C_N_2	1.6	63.0	321.1
Ru/CeO_2-C_H_2	1.6	62.4	280.6
Ru/CeO_2-C_NH_3	1.6	66.6	283.1
Ru/CeO_2	1.8	-	31.0
Ru/C	1.7	-	816.3

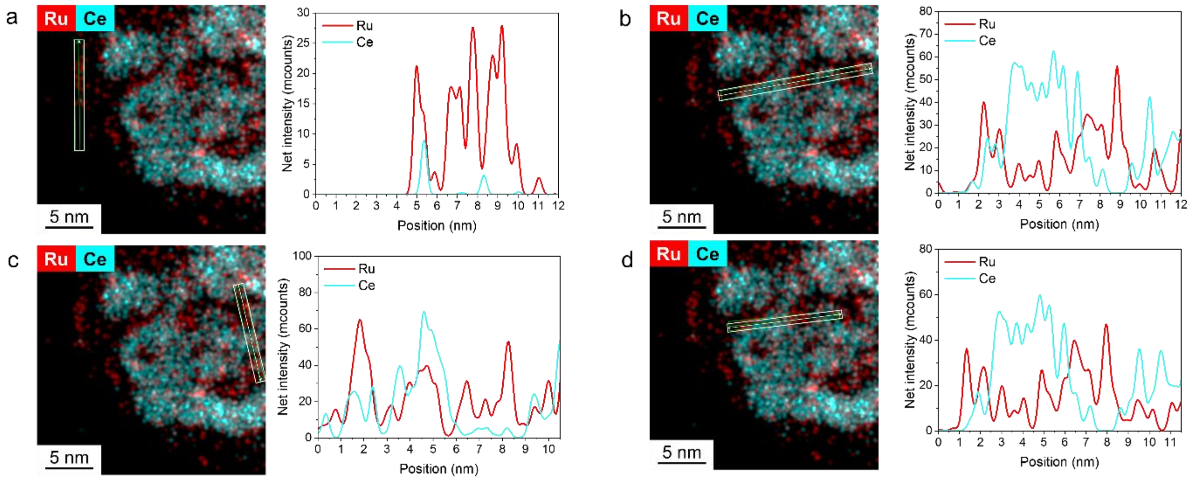

Supplementary Fig. 6 Line EDS mapping results for Ru/CeO_2-C_N_2.

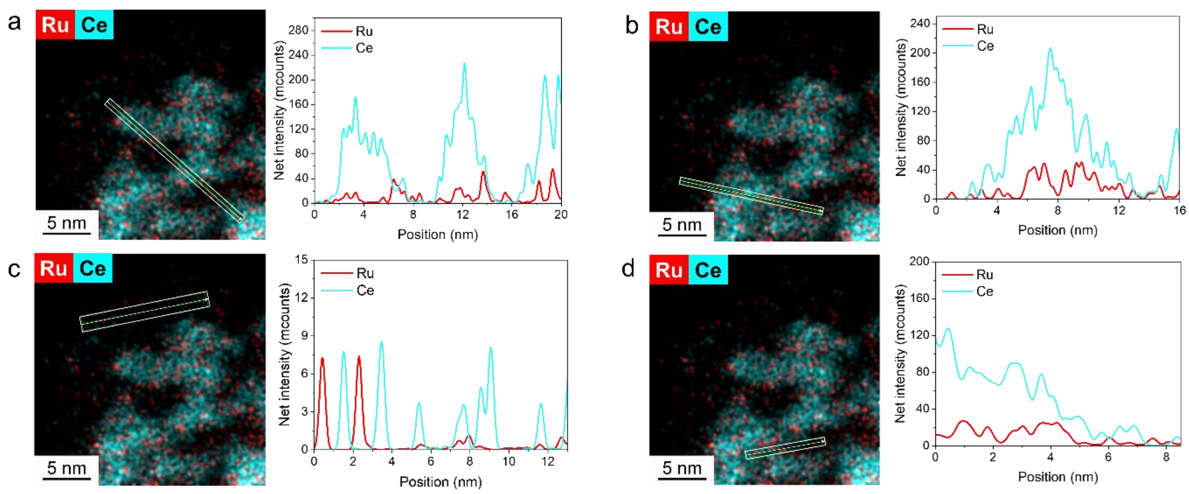

Supplementary Fig. 7 Line EDS mapping results for Ru/CeO_2-C_NH_3.

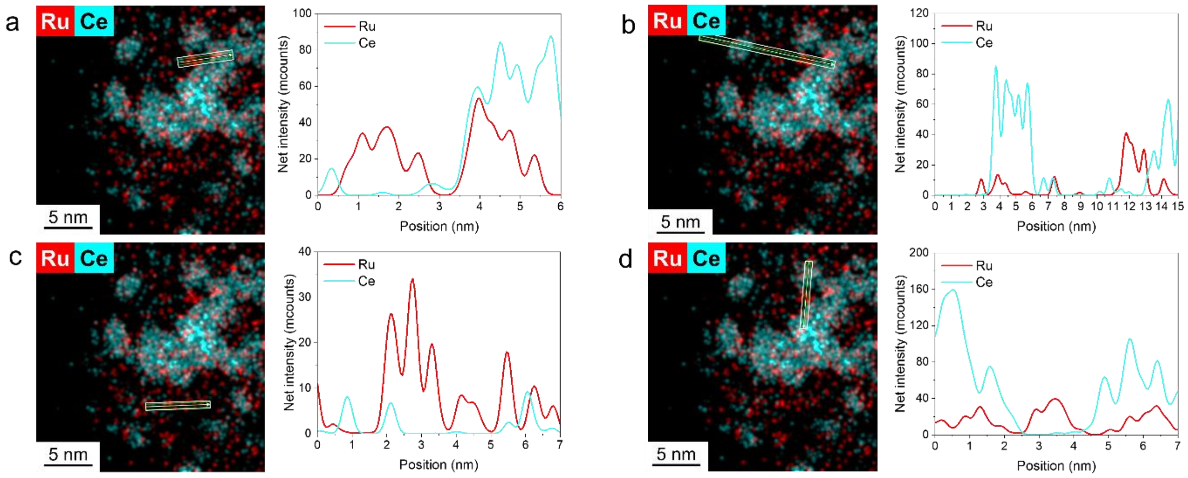

Supplementary Fig. 8 Line EDS mapping results for Ru/CeO_2-C_H_2.

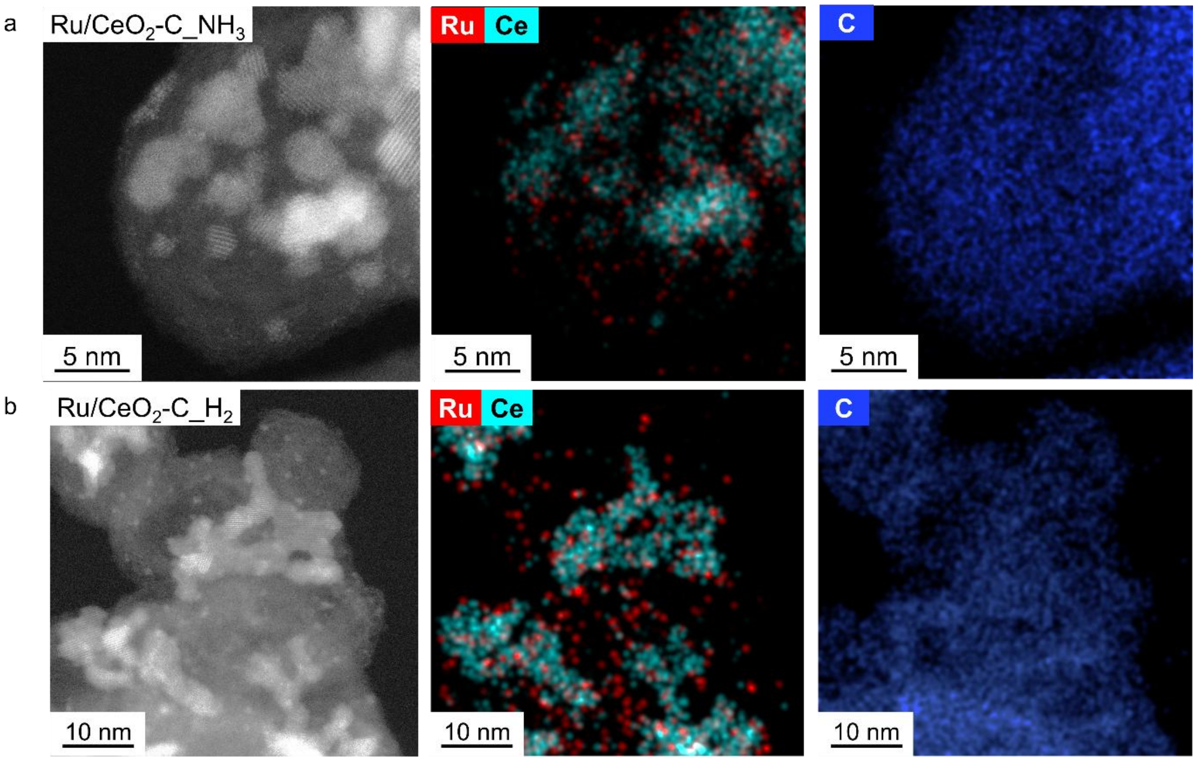

Supplementary Figure 9. Additional UHR-TEM and corresponding multi-EDS mapping images for (a) Ru/CeO_2-C_NH_3 and (c) Ru/CeO_2-C_H_2.

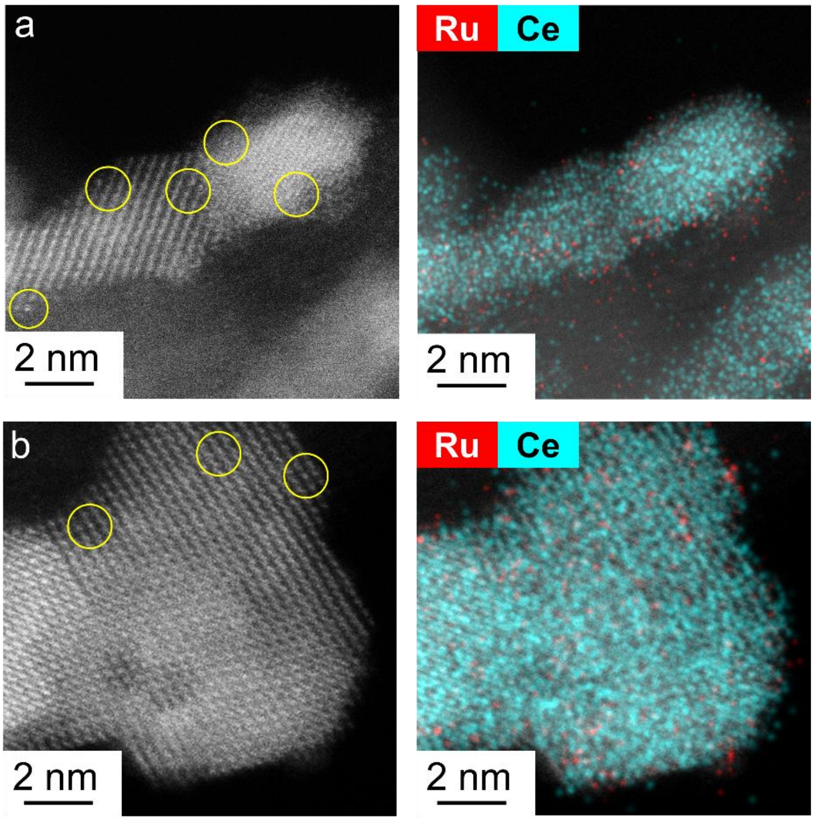

Supplementary Fig. 10 UHR-STEM images of Ru/CeO_2-C_NH_3 in 2 nm scale resolution. Sites for atomic Ru were highlighted with yellow circles.

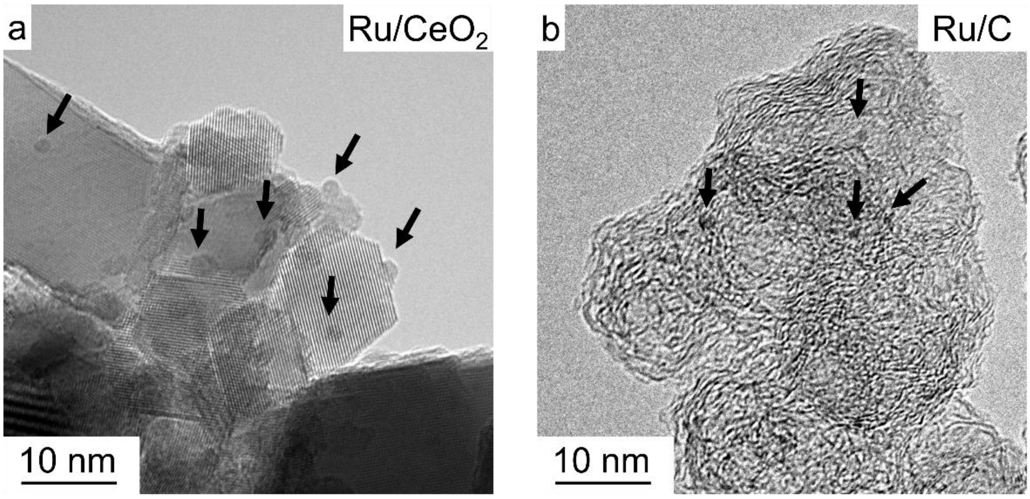

Supplementary Figure 11. TEM images of Ru/CeO_2 and Ru/C after NH_3 treatment at 500 °C for 1 h.

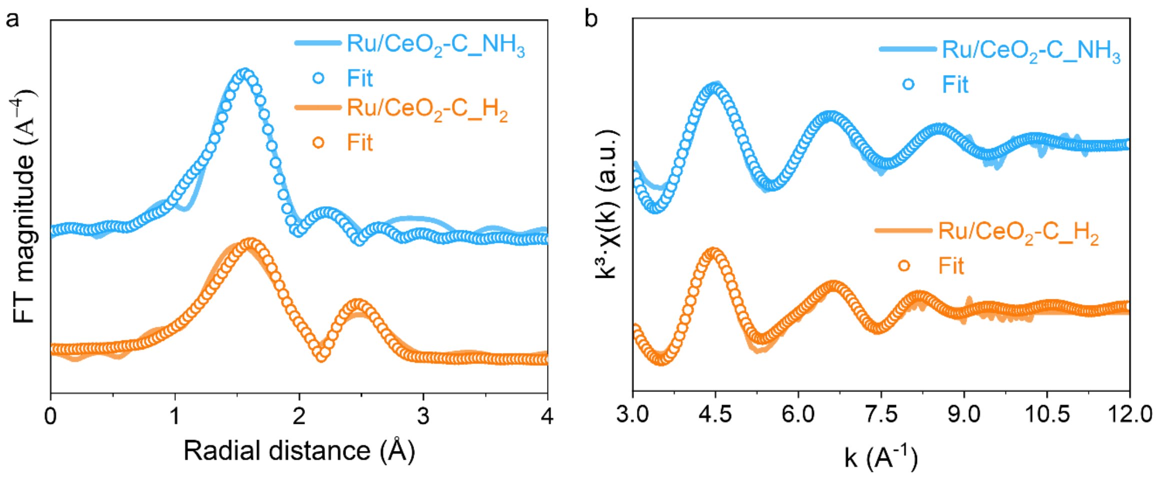

Supplementary Figure 12. EXAFS fitting results of Ru K edge for Ru/CeO_2-C_NH_3 and Ru/CeO_2-C_H_2 in (a) R space, and (b) K space. The solid lines indicate experimental data, and the dots denote the fitting results.

Supplementary Table 2. Best-fit values for the EXAFS results of Ru/CeO_2-C_NH_3 and Ru/CeO_2-C_H_2.

Sample	Path	Coordination number	Debye-Waller Factor [$\sigma^2 \cdot 10^{-3}$ / Å^2]	R [Å]	R-factor
Ru/CeO_2-C_NH_3	Ru-O	3.7 ± 0.6	8.6 ± 2.4	2.0 ± 0.07	0.026
	Ru-Ru	0.3 ± 0.1	3.0*	2.6 ± 0.1	
Ru/CeO_2-C_H_2	Ru-O	3.1 ± 0.8	12.4 ± 3.4	2.0 ± 0.1	0.015
	Ru-Ru	1.7 ± 0.9	9.8 ± 4.9	2.6 ± 0.1	

* This factor was fixed during the EXAFS fitting

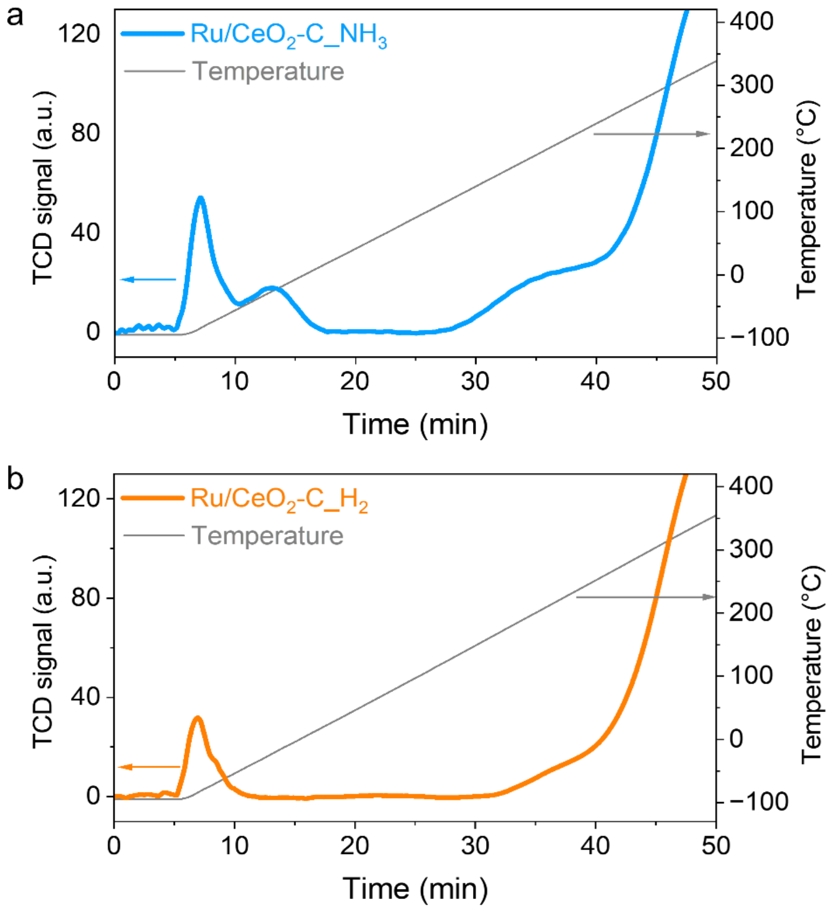

Supplementary Fig. 13 Cryo-TPO results of (a) Ru/CeO_2-C_NH_3 and (b) Ru/CeO_2-C_H_2.

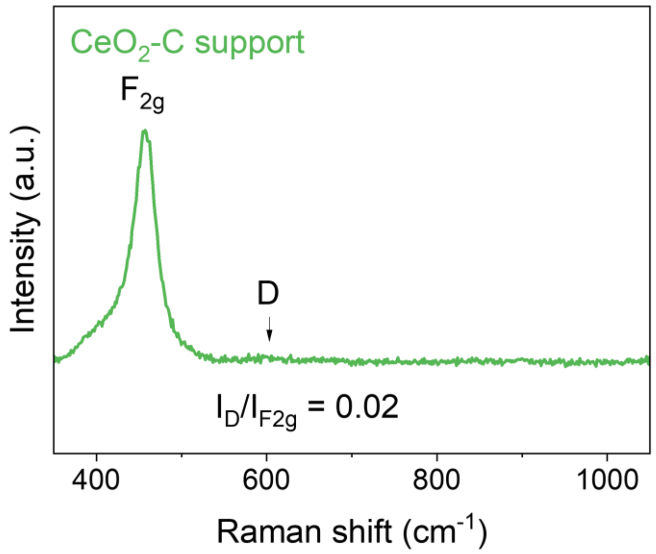

Supplementary Fig. 14 Raman spectrum result of CeO_2-C support.

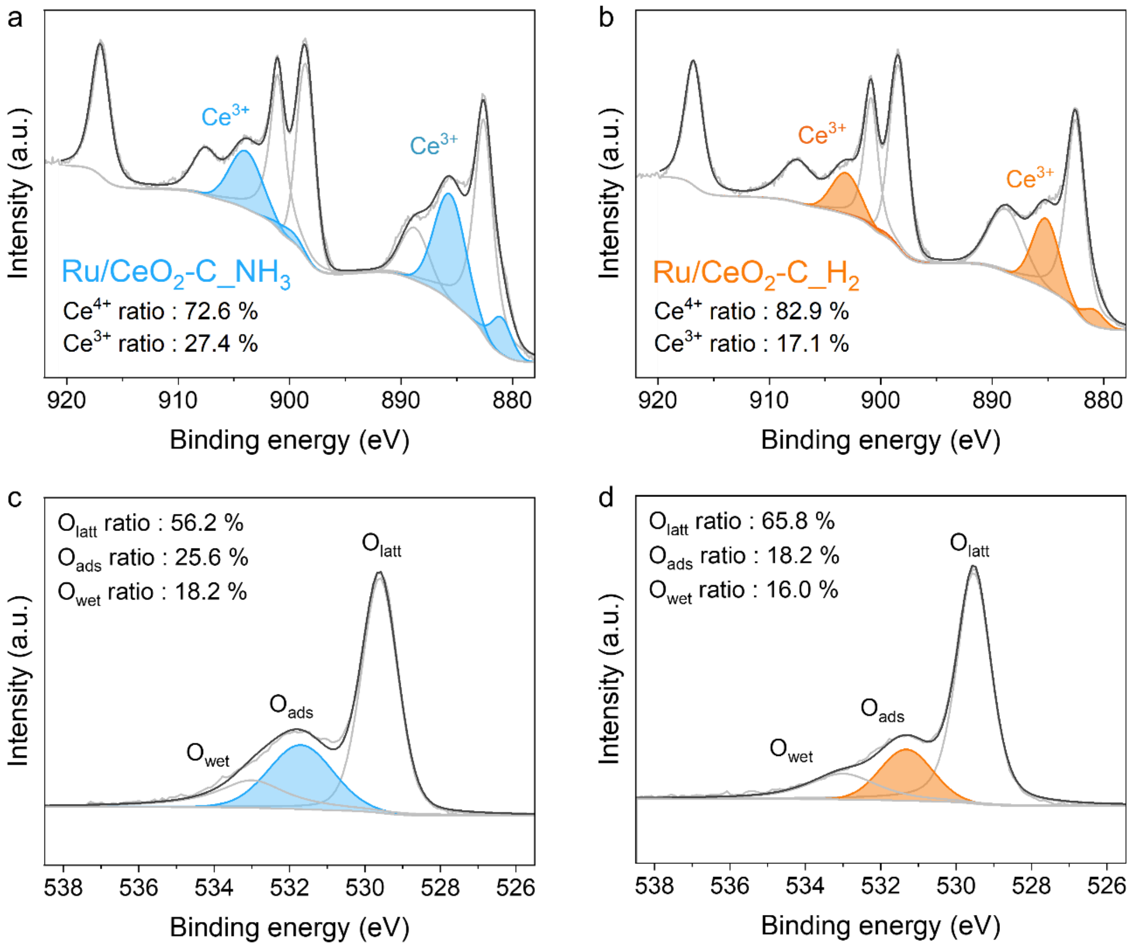

Supplementary Figure 15. Ce 3d XPS spectra of (a) Ru/CeO$_2$-C_NH$_3$, and (b) Ru/CeO$_2$-C_H$_2$, and O 1s XPS spectra for (c) Ru/CeO$_2$-C_NH$_3$, and (d) Ru/CeO$_2$-C_H$_2$.

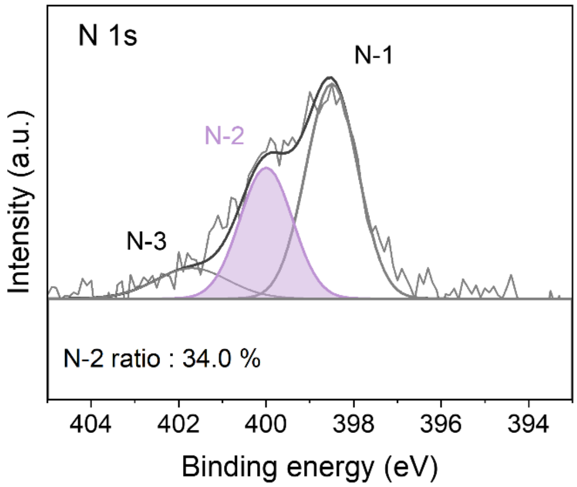

Supplementary Figure 16. N 1s XPS spectra of Ru/N-doped C (N-C).

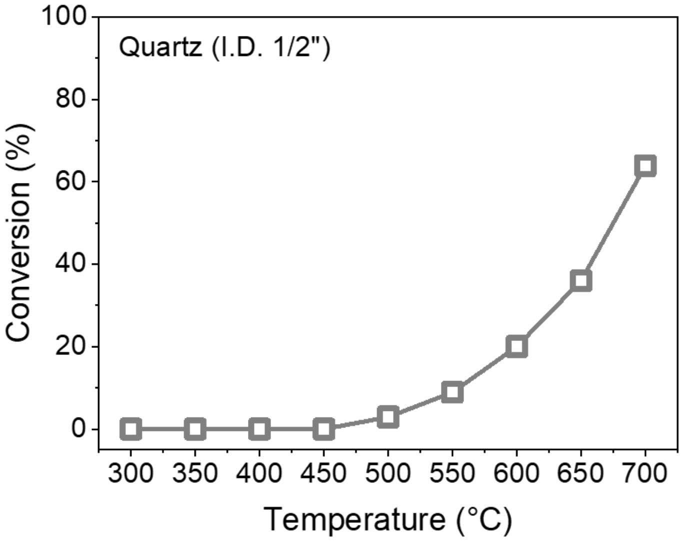

Supplementary Fig. 17 NH_3 conversion results for blank test, using quartz reactor with inner diameter of ½ inch. Ammonia flow was 40 mL/min, which corresponds to WHSV of 30,000 mL/g_{cat}·h with catalysts.

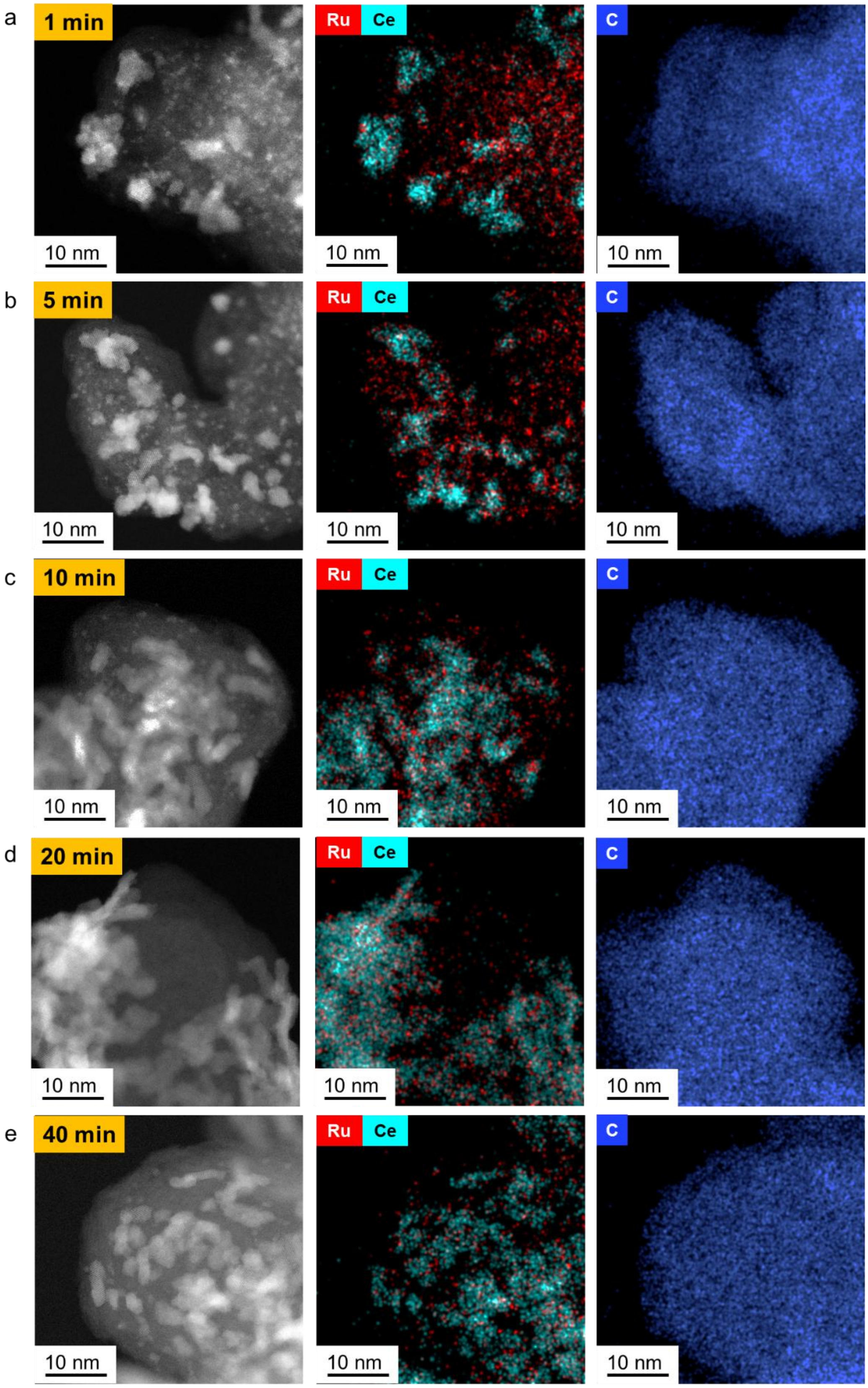

Supplementary Fig. 18 Time-resolved UHR-TEM images and corresponding multi-EDS results. Images were taken after NH_3 treatment on Ru/CeO_2-C_N_2 for 1 min, 5 min, 10 min, 20 min, and 40 min at 500 °C

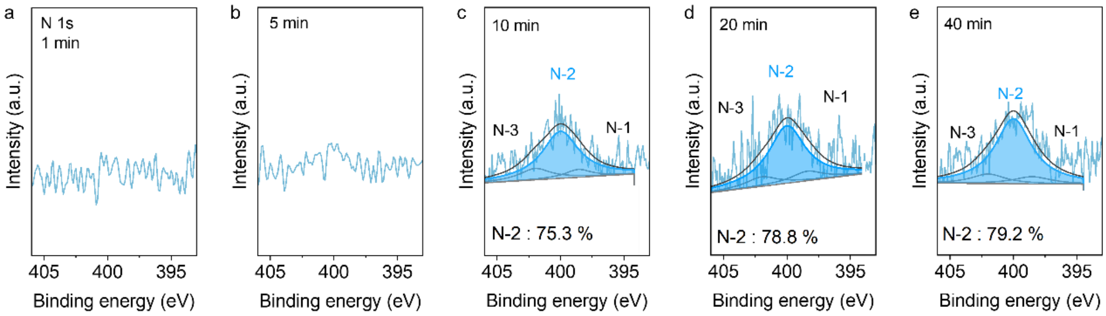

Supplementary Fig. 19 Time-resolved N 1s XPS results NH_3 treatment on Ru/CeO_2-C_N_2 for 1 min, 5 min, 10 min, 20 min, and 40 min at 500 °C.

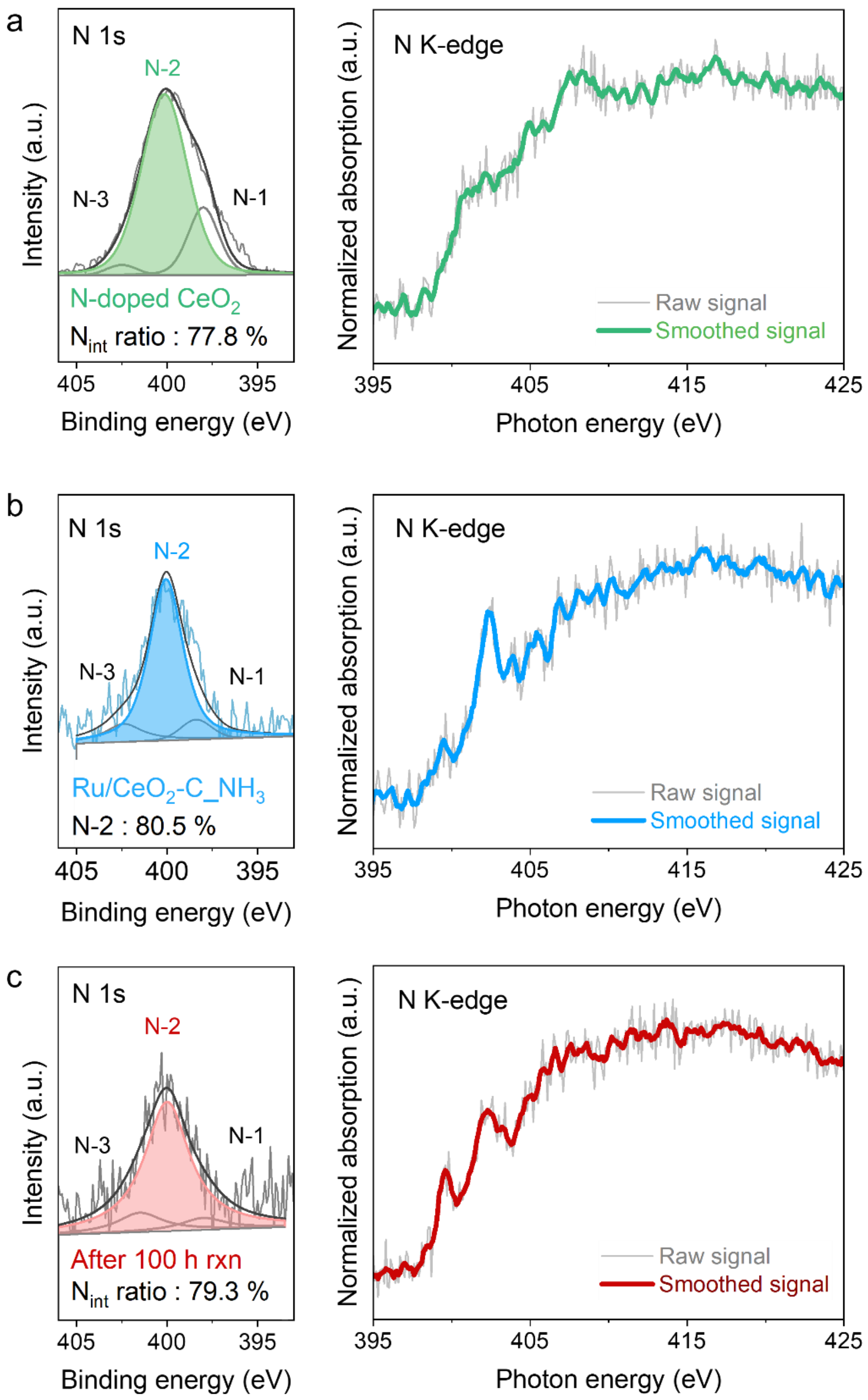

Supplementary Fig. 20 N 1s XPS and N K-edge X-ray absorption spectroscopy results of (a) N-doped CeO_2, (b) Ru/CeO_2-C_NH_3, and (c) Ru/CeO_2-C_NH_3 after 100 h of reaction.

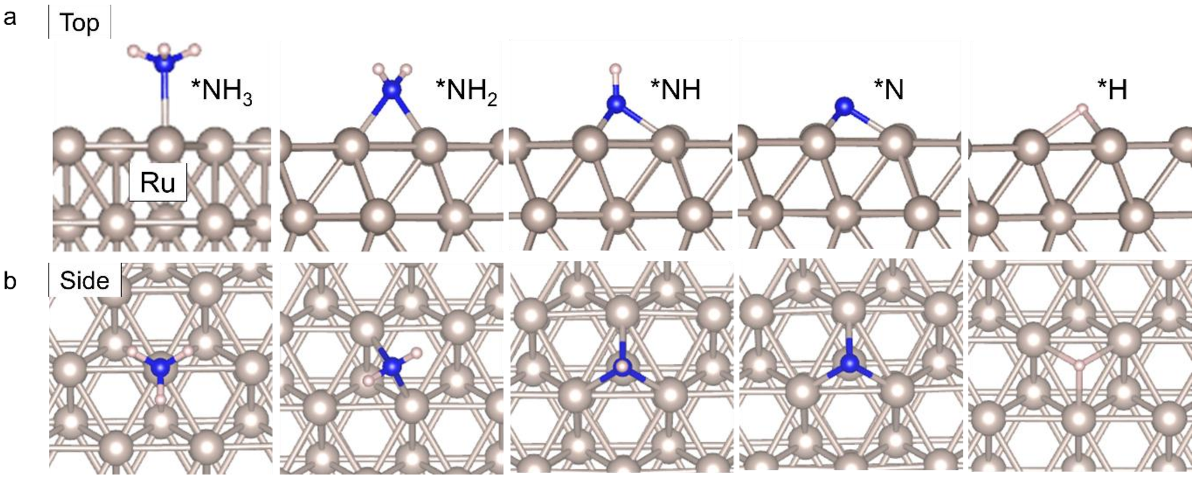

Supplementary Figure 21. (a) Top and (b) side of the most stable adsorption structure $*NH_3$, $*NH_2$, *NH, *N, and *H on Ru (0001) surface.

Supplementary Table 3. Adsorption energies (E_{ads}) and favorable adsorption sites of $*NH_x$ and *H on Ru(0001). Ejection energies (E_{eject}) of Ru-NH_x and d band center energies of Ru atom bound with above intermediates on Ru surface are contained. (T: top, B: HCP bridge, H: HCP hollow, FCC H: FCC hollow)

	Vac	$*NH_3$	$*NH_2$	*NH	*N	*H
E_{ads} (eV)	-	-0.96	-3.02	-5.38	-6.27	-2.86
Adsorption Site	-	T	B	HCP H	HCP H	FCC H
E_{eject} (eV)	8.38	8.62	8.27	9.54	8.99	8.39
d band center E (eV)	-1.34	-1.24	-1.12	-1.17	-1.31	-1.24

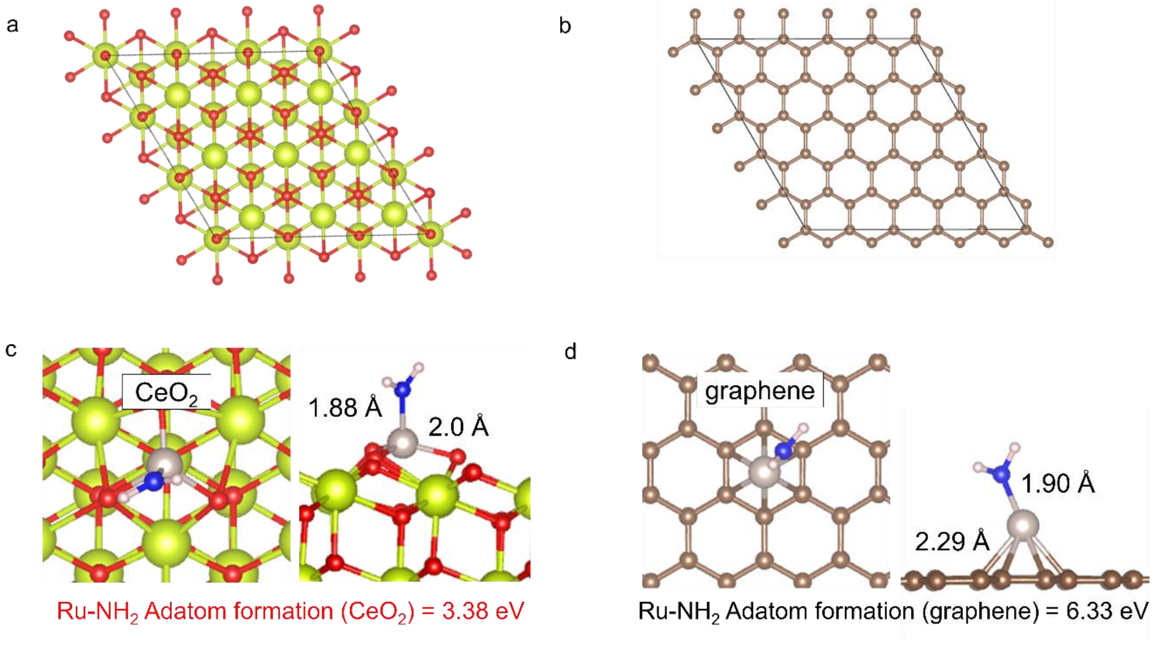

Supplementary Figure 22. (a, b) Schematics of CeO_2 and graphene supercell. (c, d) Side and Top view of the most stable adsorption structure of Ru-NH_2 on CeO_2 (111) and graphene. Distances between atoms are provided in Å. Adatom formation energies of Ru-NH_2 when it migrates from Ru (0001) to CeO_2 and graphene are written below the figure.

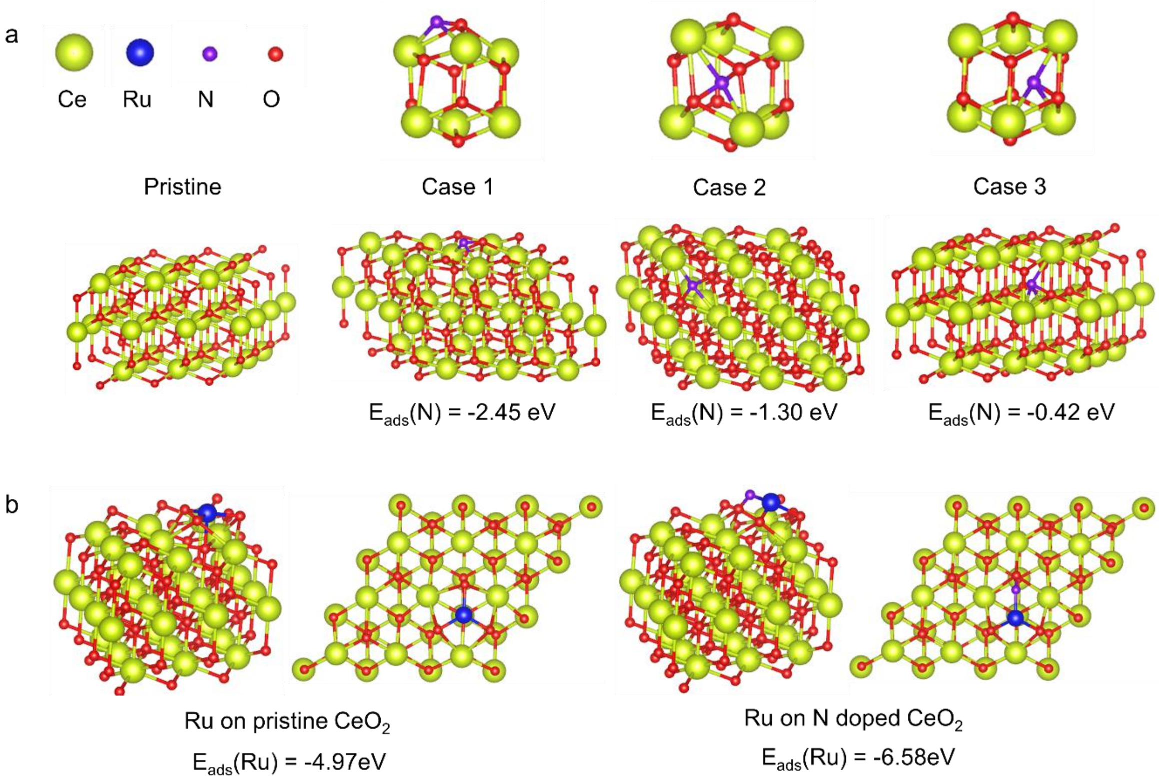

Supplementary Fig. 23 (a) The atomic configuration and binding energy of N atom for three potential sites for N-doping. Case 1 for surface interstitial N stabilization, case 2 and 3 for subsurface interstitial N stabilization. (b) Adsorption energy calculation of a single Ru atom on the surface pristine CeO_2 (111) and N-doped CeO_2 (111).

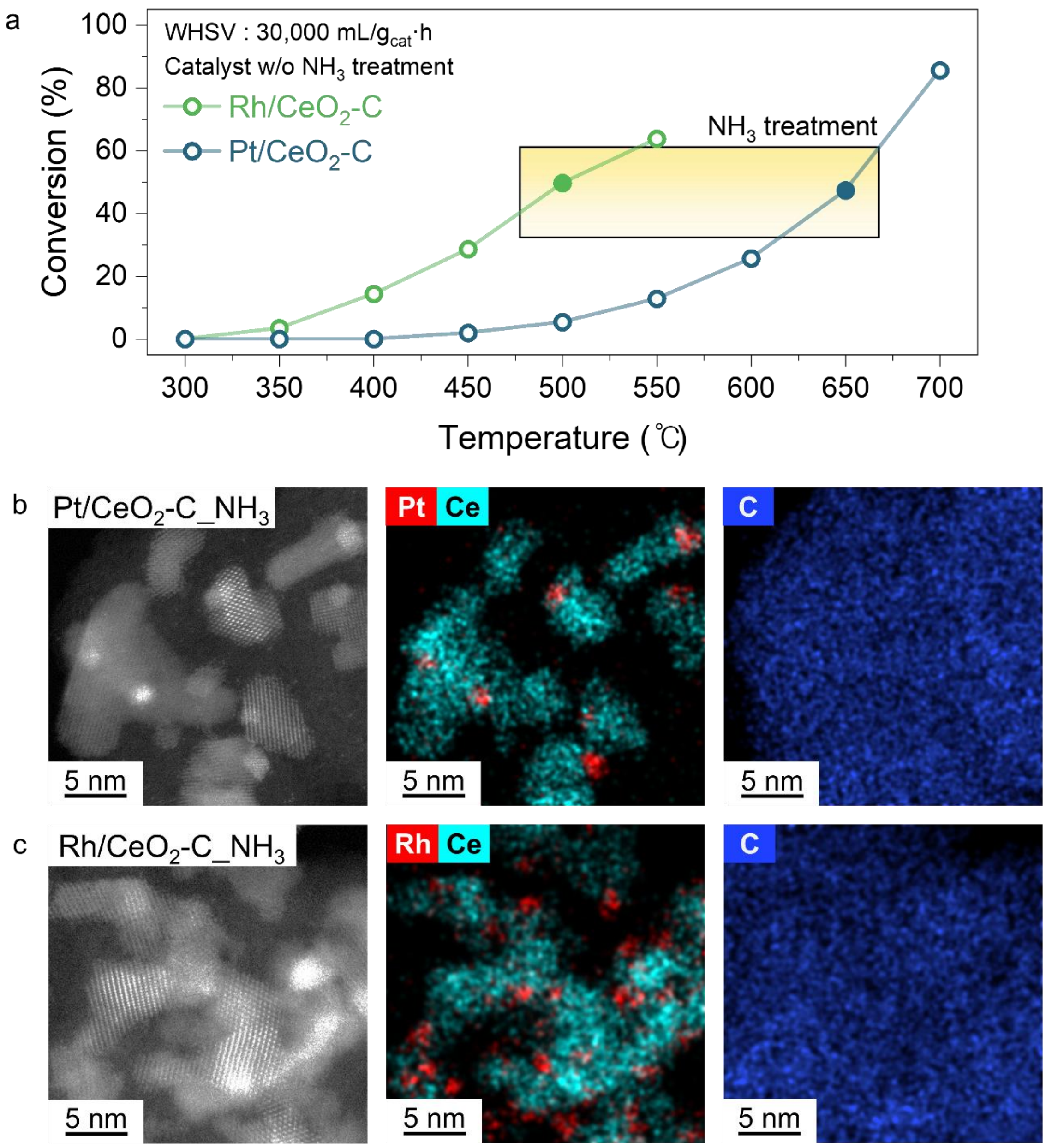

Supplementary Figure 24. Ammonia decomposition result of Pt/CeO_2-C and Rh/CeO_2-C pretreated in N_2. Ammonia decomposition was performed with a gas flow of 100 % NH_3 with the WHSV of 30,000 mL/g$_{cat}$·h. UHR-TEM and corresponding multi-EDS mapping images for (b) Pt/CeO_2-C and (c) Rh/CeO_2-C after NH_3 treatment at 500 °C for 1 h.

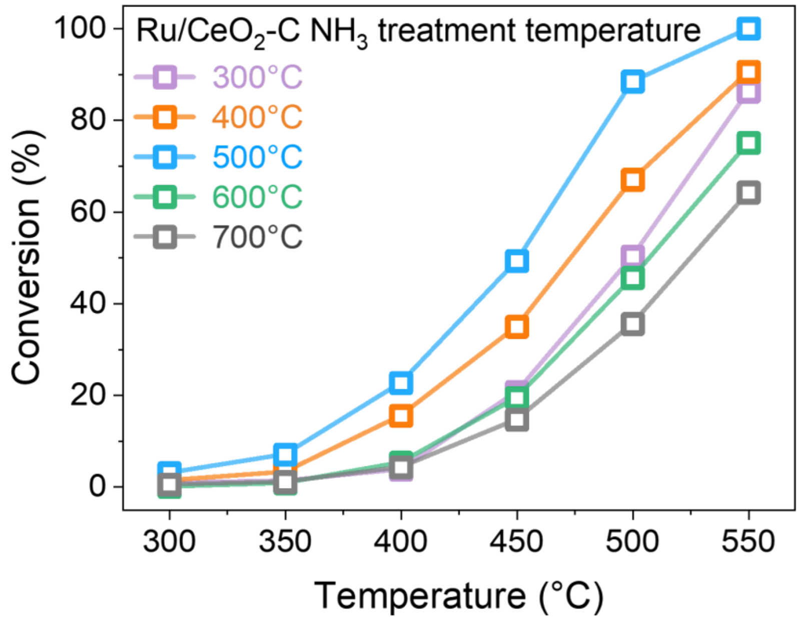

Supplementary Figure 25. Ammonia conversion for Ru/CeO_2-C with different NH_3 treatment temperature in ambient pressure. Each pretreatments was conducted for 1 hour. Ammonia decomposition was performed with a gas flow of 100 % NH_3 with the WHSV of 30,000 mL/g_{cat}·h.

Supplementary Table 4. Comparison of H_2 production rate of Ru/CeO_2-C_NH_3 with literature values at various reaction conditions.

Ref	Catalysts	Ru content (wt %)	Reaction temperature (°C)	Reaction pressure (bar)	H_2 production (mol/g_{Ru}·h)
This work	Ru/CeO_2-C_NH_3	1.6	400	1	26.1
			450		56.7
			500		101.7
			550		115.1
			400	9	23.1
			450		54.1
			500		96.4
			550		110.9
1	Ru-K/CaO	3	450	10	9.5
			500		14.8
			550		17.1
2	Ru/Al_2O_3	8.5	450	10	1.4
			500		1.6
			550		1.7
3	Ru/ZrO_2	5.6	400	1	8.5
4	Ru/BN	1.0	400	1	40.5
5	Ru/CeO_2/CNT	1.5	450	1	85.9
6	Ru/Ba-ZrO_2	3.0	450	1	2.3
7	Ru/MgO	5.0	450	1	31.4
8	Ru/BHA	3.0	450	1	25.8
9	Ru/SiO_2	5.0	450	1	22.1
10	Ru/Cr_2O_3	10.0	500	1	5.8
11	Ru/La-ZrO_2	3.0	500	1	46.2
12	K-Ru/Zeolite-Y	2.2	500	1	20.9
13	Ru/CNT	5.0	550	1	31.2
14	Ru/N-CNT	1.0	550	1	154.6

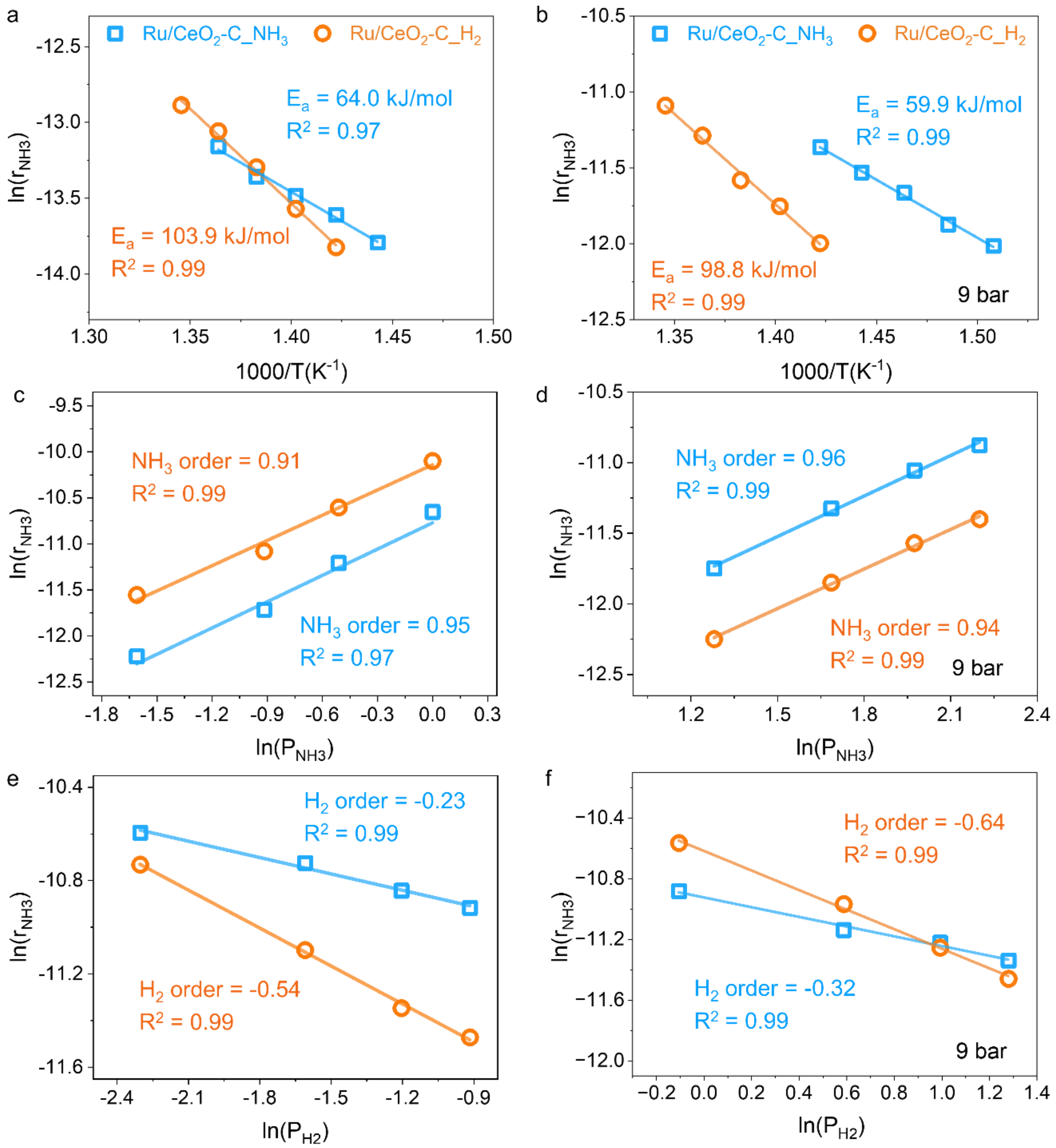

Supplementary Figure 26. (a, b) Activation energy, (c, d) reaction orders with respect to NH_3 partial pressure, and (e, f) reaction orders with respect to H_2 partial pressure of Ru/CeO_2-C_NH_3 and Ru/CeO_2-C_H_2. These experiments were performed at ambient pressure. (a, c, e) were obtained under ambient pressure, while (b, d, f) were measured under elevated pressure (9 bar).

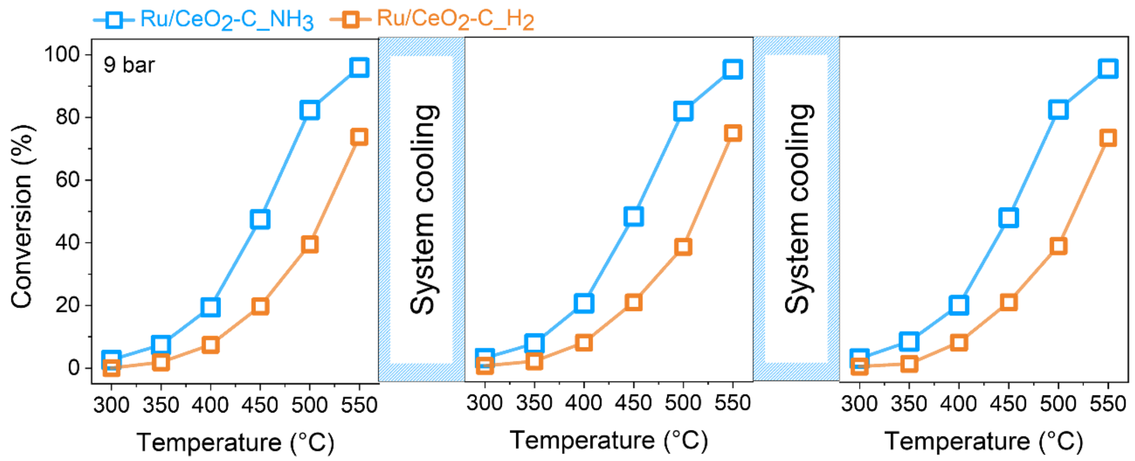

Supplementary Fig. 27 Catalytic performance of Ru/CeO_2-C_NH_3 and Ru/CeO_2-C_H_2 for high-pressure ammonia decomposition reaction for three repeated cycles. NH_3 decomposition was conducted using 100% NH_3 with a WHSV of 30,000 mL/g_{cat}·h.

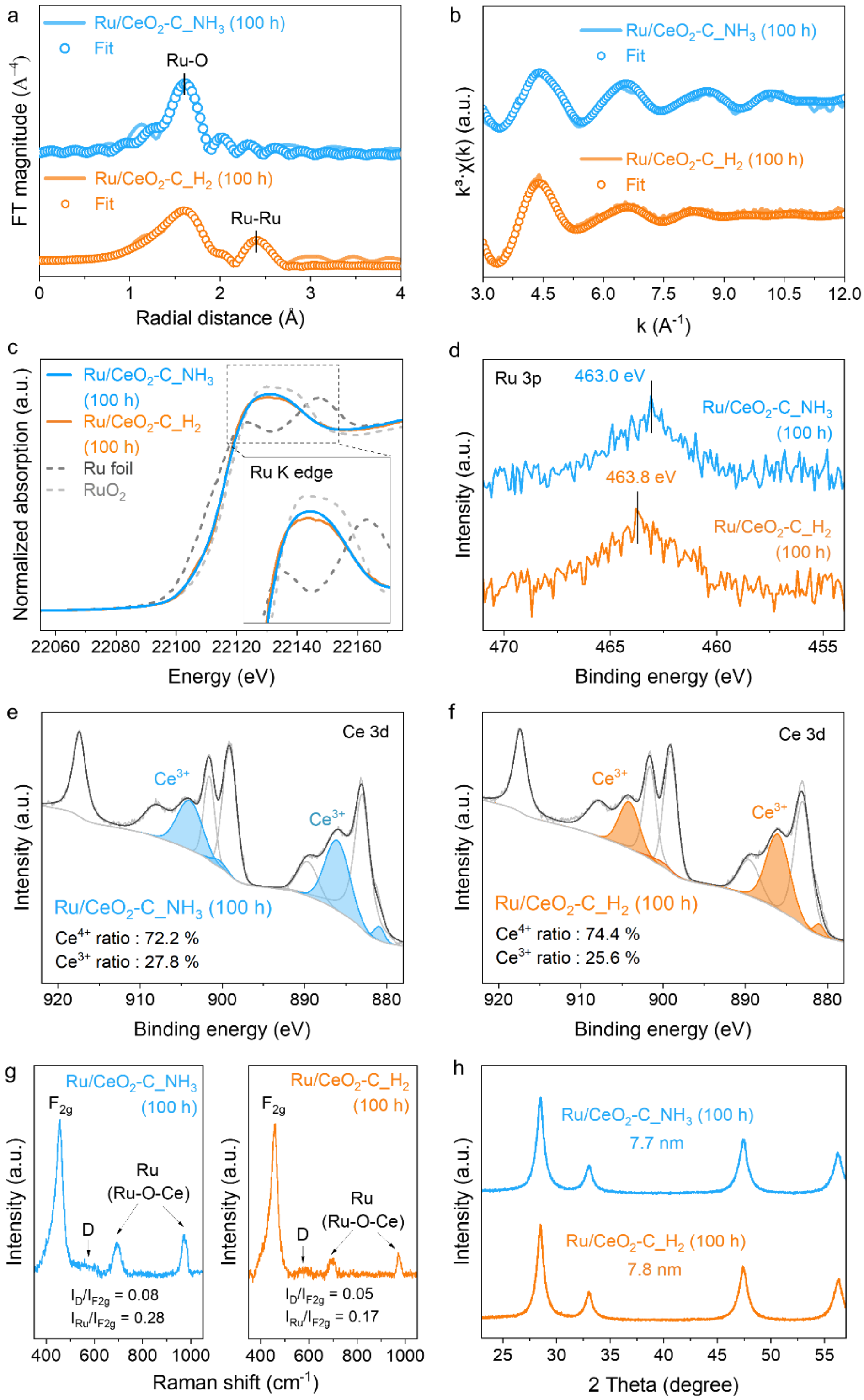

Supplementary Figure 28. EXAFS fitting results of Ru K edge for Ru/CeO_2-C_NH_3 and Ru/CeO_2-C_H_2 after high-pressure long term durability test in (a) R space, and (b) K space. The solid lines indicate experimental data, and the dots denote the fitting results. (c) XANES, (d-f) Ru 3p and Ce 3d XPS, (g) Raman, and (h) XRD results of Ru/CeO_2-C_NH_3 and Ru/CeO_2-C_H_2 after high-pressure long term durability test.

Supplementary Table 5. Best-fit values for the EXAFS results of Ru/CeO_2-C_NH_3 and Ru/CeO_2-C_H_2 after 100 h of long-term reaction at high pressure.

Sample	Path	Coordination number	Debye-Waller Factor [$\sigma^2 \cdot 10^{-3}$ / $Å^2$]	R [Å]	R-factor
Ru/CeO_2-C_NH_3 (100 h)	Ru-O	3.9 ± 0.6	5.6 ± 1.9	2.0 ± 0.1	0.028
	Ru-Ru	0.2 ± 0.2	3.0*	2.6 ± 0.1	
Ru/CeO_2-C_H_2 (100 h)	Ru-O	3.4 ± 0.4	6.5 ± 1.6	2.0 ± 0.1	0.024
	Ru-Ru	1.4 ± 0.5	6.8 ± 2.0	2.6 ± 0.1	

* This factor was fixed during the EXAFS fitting

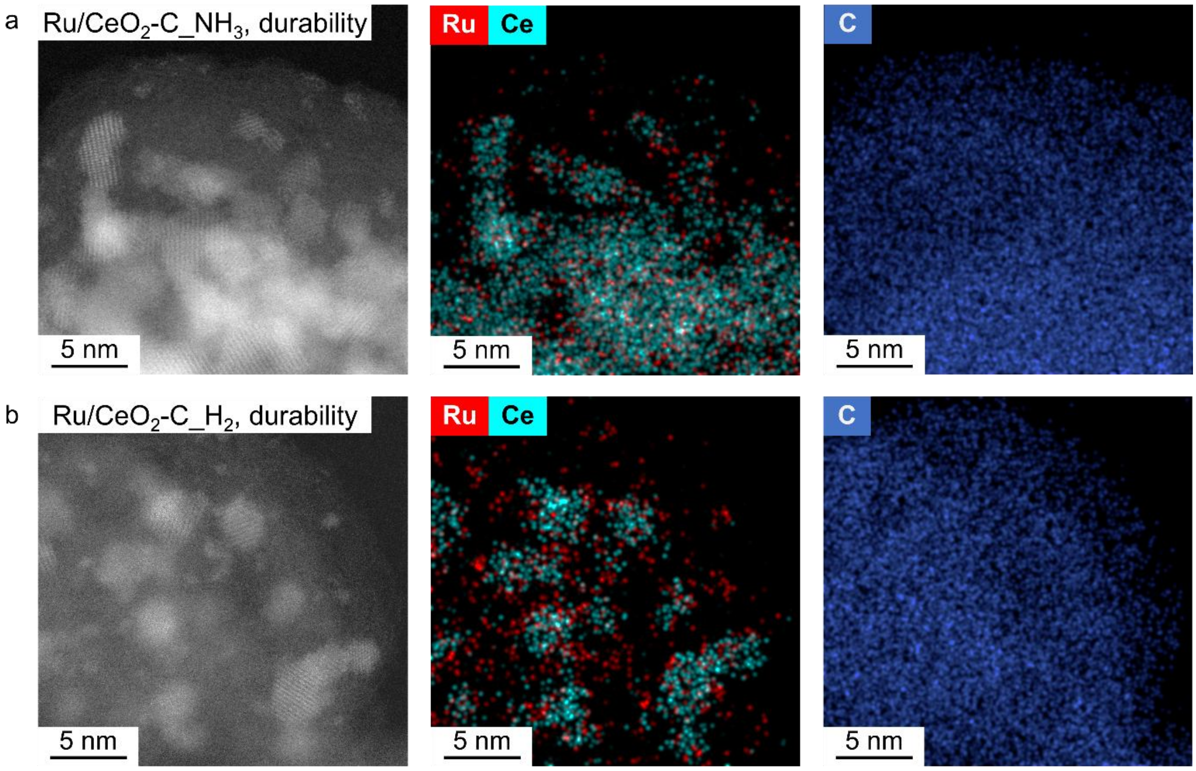

Supplementary Figure 29. UHR-TEM and corresponding multi-EDS mapping images for (a) Ru/CeO_2-C_NH_3 and (b) Ru/CeO_2-C_H_2 after high-pressure long term reaction.

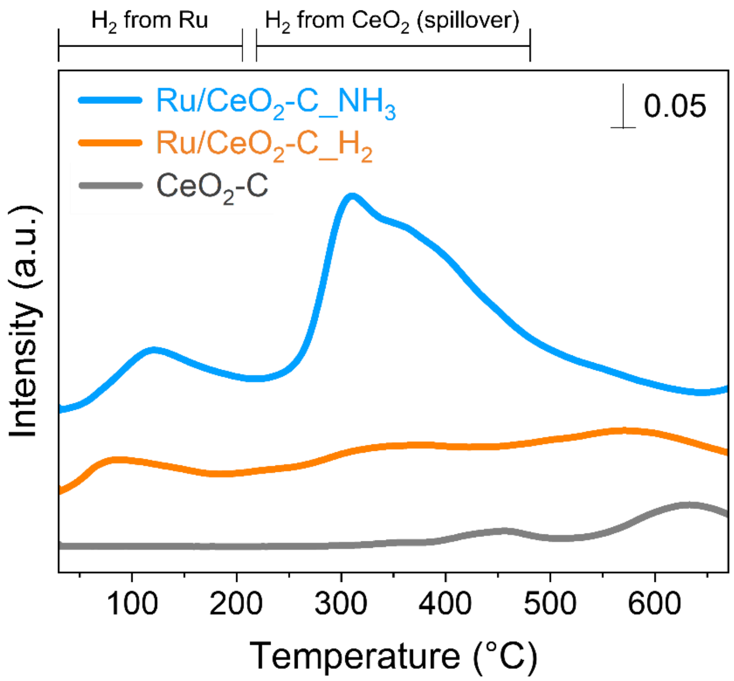

Supplementary Fig. 30 H_2-TPD results of CeO_2-C, Ru/CeO_2-C_H_2, and Ru/CeO_2-C_NH_3.

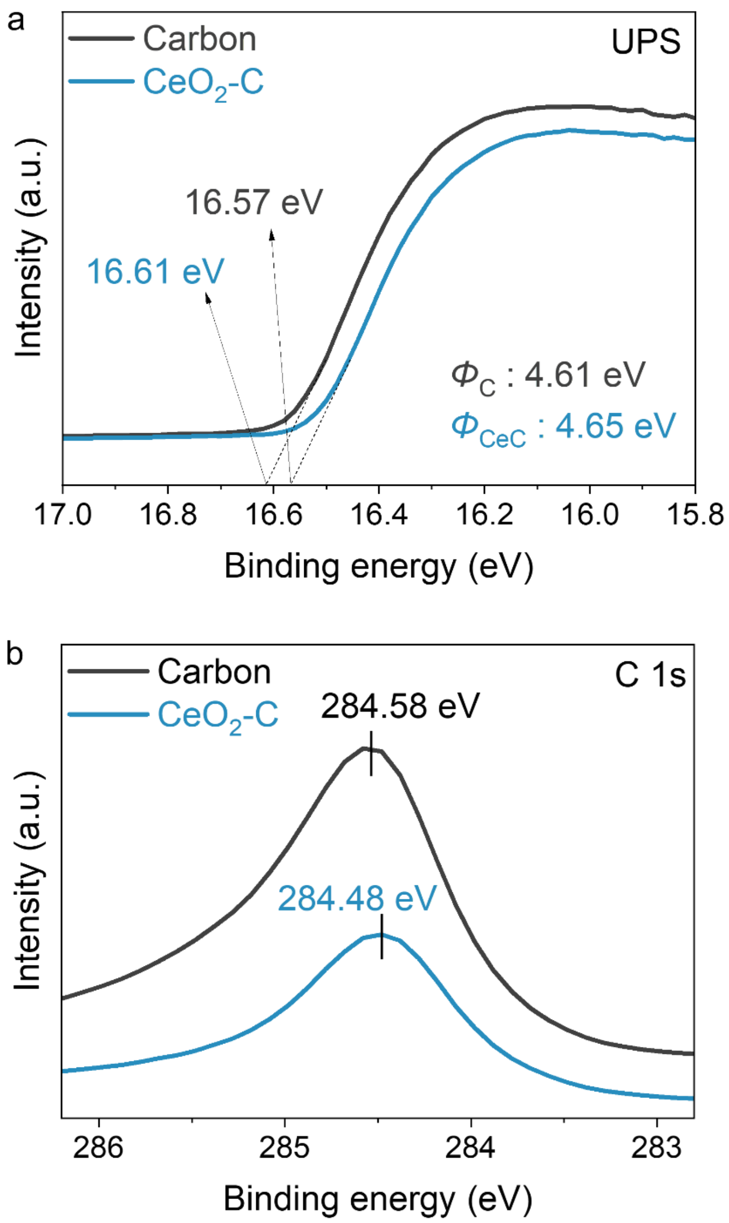

Supplementary Figure 31. (a) UPS spectra and (b) C 1s XPS of CeO_2-C and carbon support.

SUPPLEMENTARY REFERENCES

1 Sayas, S. *et al.* High pressure ammonia decomposition on Ru–K/CaO catalysts. *Catalysis Science & Technology* **10**, 5027-5035 (2020). https://doi.org/10.1039/D0CY00686F

2 Di Carlo, A., Vecchione, L. & Del Prete, Z. Ammonia decomposition over commercial Ru/Al2O3 catalyst: An experimental evaluation at different operative pressures and temperatures. *Int. J. Hydrogen Energy.* **39**, 808-814 (2014). https://doi.org/https://doi.org/10.1016/j.ijhydene.2013.10.110

3 Furusawa, T. *et al.* Preparation of Ru/ZrO2 Catalysts by NaBH4 Reduction and Their Catalytic Activity for NH3 Decomposition To Produce H2. *Ind. Eng. Chem. Res.* **55**, 12742-12749 (2016). https://doi.org/10.1021/acs.iecr.6b03265

4 Kang, S. *et al.* Heteroepitaxial Growth of B5-Site-Rich Ru Nanoparticles Guided by Hexagonal Boron Nitride for Low-Temperature Ammonia Dehydrogenation. *Advanced Materials* **35**, 2203364 (2023). https://doi.org/https://doi.org/10.1002/adma.202203364

5 Lv, L. *et al.* Carbon and Oxygen Double Defects-Enhanced Ru-Based Catalyst for Ammonia Decomposition. *Angew. Chem. Int. Ed.* **64**, e202501898 (2025). https://doi.org/https://doi.org/10.1002/anie.202501898

6 Wang, Z., Qu, Y., Shen, X. & Cai, Z. Ruthenium catalyst supported on Ba modified ZrO2 for ammonia decomposition to COx-free hydrogen. *Int. J. Hydrogen Energy.* **44**, 7300-7307 (2019). https://doi.org/https://doi.org/10.1016/j.ijhydene.2019.01.235

7 Ju, X. *et al.* Mesoporous Ru/MgO prepared by a deposition-precipitation method as highly active catalyst for producing COx-free hydrogen from ammonia decomposition. *Appl. Catal. B* **211**, 167-175 (2017). https://doi.org/https://doi.org/10.1016/j.apcatb.2017.04.043

8 Wang, Z., Cai, Z. & Wei, Z. Highly Active Ruthenium Catalyst Supported on Barium Hexaaluminate for Ammonia Decomposition to COx-Free Hydrogen. *ACS Sustain. Chem. Eng.* **7**, 8226-8235 (2019). https://doi.org/10.1021/acssuschemeng.8b06308

9 Wang, F. *et al.* The dispersed SiO2 microspheres supported Ru catalyst with enhanced activity for ammonia decomposition. *International Journal of Hydrogen Energy* **46**, 20815-20824 (2021). https://doi.org/https://doi.org/10.1016/j.ijhydene.2021.03.205

10 Li, L., Wang, Y., Xu, Z. P. & Zhu, Z. Catalytic ammonia decomposition for CO-free hydrogen generation over Ru/Cr2O3 catalysts. *Appl. Catal. A* **467**, 246-252 (2013). https://doi.org/https://doi.org/10.1016/j.apcata.2013.07.003

11 Lorenzut, B. *et al.* Embedded Ru@ZrO2 Catalysts for H2 Production by Ammonia Decomposition. *ChemCatChem* **2**, 1096-1106 (2010). https://doi.org/https://doi.org/10.1002/cctc.201000097

12 Cha, J. *et al.* Highly monodisperse sub-nanometer and nanometer Ru particles confined in alkali-exchanged zeolite Y for ammonia decomposition. *Appl. Catal. B* **283**, 119627 (2021). https://doi.org/https://doi.org/10.1016/j.apcatb.2020.119627

13 Li, L., Zhu, Z. H., Yan, Z. F., Lu, G. Q. & Rintoul, L. Catalytic ammonia decomposition over Ru/carbon catalysts: The importance of the structure of carbon support. *Appl. Catal. A* **320**, 166-172 (2007). https://doi.org/https://doi.org/10.1016/j.apcata.2007.01.029

14 Chen, J. *et al.* Effects of nitrogen doping on the structure of carbon nanotubes (CNTs) and activity of Ru/CNTs in ammonia decomposition. *Chem. Eng. J.* **156**, 404-410 (2010). https://doi.org/https://doi.org/10.1016/j.cej.2009.10.062
