# Supplementary material for: Active nitrogen mediated selective ruthenium migration on ceria for high pressure ammonia decomposition
Source: Nat Commun. 2026 Jun 15;17:7541. doi: 10.1038/s41467-026-74205-7 (PMC13408114; doi:10.1038/s41467-026-74205-7)
Supplement: Supplementary file 2 — Transparent Peer Review file [file 41467_2026_74205_MOESM2_ESM.pdf]

nature portfolio

# Peer Review File

## Active Nitrogen Mediated Selective Ruthenium Migration on Ceria for High Pressure Ammonia Decomposition

Corresponding Author: Dr Hyuntae Sohn

**This file contains all reviewer reports in order by version, followed by all author rebuttals in order by version.**

Version 0:

Reviewer comments:

Reviewer #1

(Remarks to the Author)
In the present work Ru on CeC together with different RuCeO2 and RuC catalysts were prepared and tested in the Ammonia Decomposition. The catalysts were extensively characterized by different techniques including TEM, EXAFS and XPS. In general the paper is well written, and the experimental data support the discussion. The main conclusion of this paper is that NH3 induces the selective migration of ruthenium (Ru) atoms onto CeO2, forming a durable system. My impression is that this paper might be suitable for publication after addressing the following points

In the introduction the choice of ceria-coated carbon support as support should be better motivated

Pag 7 line 137 "After the NH3 treatment, most of the ruthenium located on the carbon disappeared…)
Figure 1b is not enough to demonstrate that Ru is present only on CeO2. I still see Ru species on carbon. Moreover the real content of Ru after the treatment should be shown and better discussed

Reviewer #2

(Remarks to the Author)
In this manuscript, the authors prepared a catalyst for high-pressure ammonia decomposition reaction by constructing small-sized CeO2 nanoparticles on a carbon support and loading Ru. The authors found that NH3 treatment of the "Ru/CeC" catalyst induced a selective atomic rearrangement of Ru atoms on the CeO2 nanoparticles, resulting in the formation of well-defined and stable Ru single atoms on the CeO2 surface ("Ru/CeC_NH3"). This phenomenon is very interesting, but the current data provided in the manuscript are insufficient to support it, and there are several important problems that need to be discussed. Therefore, I believe this manuscript requires major revision.
1. Regarding the choice of the reaction system. According to the reaction equation 2NH3 = 3H2 + N2, low pressure favors the ammonia decomposition reaction. Why test reactions under high pressure? If it is due to practical process requirements, what is the cost assessment? Moreover, the scientific investigation of the Ru state in this manuscript did not employ high-pressure NH3 conditions, and research on the Ru state under high-pressure $NH_3$ conditions is lacking.
2. In terms of catalytic performance, the current catalytic activity achieved with 1.6 wt% Ru loading is not very competitive and is lower than that of most low-loading Ru-based catalysts using rare-earth oxides as supports.
3. Regarding the naming of the catalyst, "CeC" can easily be mistaken for cerium carbide (CeC2). It is recommended to change it to another naming convention.
4. Based on the characterization results, NH3 treatment enables Ru species to disperse more on CeO2 and remain highly dispersed. However, I believe it is currently not justified to claim it as single atoms, and it cannot be confirmed that Ru single atoms can remain stable under high-temperature ammonia decomposition conditions, as there is no in situ characterization to support this.
5. Why do Ru particles still exist on "Ru/CeC_H2" after 100 hours of catalyzing the ammonia decomposition reaction, and why have Ru species not completely migrated to CeO2? This seems inconsistent with the main argument of the manuscript. If "Ru/CeC_H2" is treated with air at low temperature (e.g., 300 °C) and then tested for ammonia decomposition, how would its activity and structure change?
6. Will "Ru/CeC_NH3" and "Ru/CeC_H2" yield the same catalyst structure when catalyzing the ammonia decomposition reaction? Will the second or third round of activity test results be the same?

7. Figure S3 needs to provide quantitative data on the proportions of the various species.
8. For the “CeC” supports calcined at different temperatures, the authors concluded via H2-TPR that the sample calcined at 550 °C is optimal. However, the size of CeO2 in the sample calcined at 300 °C is smaller, and I am curious about the activity of the catalyst prepared by loading Ru on the “CeC-300 °C”.
9. “Ru/CeC_H2” has already been reduced at 500 °C, so why does it still show reduction peaks at low temperatures in the result of H2-TPR? Similarly, the reduction peaks of “Ru/CeC_NH3” also needs to be confirmed.
10. The peak of D in Fig. 2d is very small. Generally, defect-rich CeO2 exhibits a significant D peak.
11. In Fig. 3b, the pure support "CeC" and C achieve 80-90% conversion at 700 °C. I believe this data is highly problematic. Please provide the activity results of a blank reactor test.
12. After treating "CeC" with $NH_3$ and then loading Ru, will Ru still deposit on the C?
13. The Ea in this manuscript was so low. The NH3 conversion was generally controlled below 15% during the test process. In addition, whether the test process was affected by diffusion effect?
14. In Fig. 5f, the binding energy ranges of C 1s and Ru 3d highly overlap, requiring comprehensive analysis.

Reviewer #3

(Remarks to the Author)
In the submitted manuscript, an ingenious concept that a novel strategy of "active nitrogen-mediated migration" is reported, which is different from the traditional pre-engineered carrier (bottom-up) or high-temperature physical migration (top-down) methods. The reactant (NH3) itself is used as a chemical medium to dynamically construct a stable active interface under reaction conditions. However, there are still several issues to be clarified before suitable for publication.
1. The article claims that "top-down syntheses using active nitrogen species remain largely unexplored", but the review of the existing literature in the introduction is not comprehensive and in-depth enough. It is not possible to clearly define the essential differences and comparative advantages between its "active nitrogen-mediated" strategy and the known "chemical media" (such as O2, CO, H2O)-guided metal migration strategies. This led to the innovation of the work being exaggerated, and it seemed that it was just a "replacement" of a known concept (changing the medium from O2/CO to NH3). The author should revise the introduction to provide a more comprehensive and critical review of existing top-down strategies (including those induced by chemical media), and on this basis, accurately and humbly explain what the unique contribution of this work is. Was it the discovery of a new medium? Or is it discovered that this medium has a unique dual function of building “in situ” and stabilizing the interface in a specific reaction (ammonia decomposition)? A stronger literature base or more experimental data should be needed to support its innovative claims.
2. The core of the article is the "selective" migration of Ru atoms to CeO2. However, TEM/EDS mapping (Figure 1), as the main evidence, has limited persuasiveness. Mapping shows that the Ru signal is enriched in the CeO2 region after NH3 treatment, but it cannot quantitatively prove that "Ru on carbon completely disappears", nor can it rule out the possibility of a small number of small clusters. This "selectivity" is more of a qualitative description and lacks statistical proof. More precise quantitative data must be provided, for example, line-scan EDS is used to quantitatively compare the Ru signal intensity in different areas (on CeO2 particles, pure carbon area).
3. The mentioned “(Ru) atoms onto ceria (CeO2) domains, forming a durable single atom structure” in the abstract and the subheading “Selective migration of Ru atoms onto CeO2 into single atom structure” under “RESULTS AND DISCUSSION” part, both illustrate the existence of Ru single atom in the CeO2, the single-atom dispersion of Ru on the CeO2 surface should be directly observed and counted through atomic resolution STEM-HAADF, and compared with the sample before treatment. Moreover, the difference on the state of catalytic materials in ammonia among that in hydrogen and nitrogen is originated from the participation in the process of ammonia decomposition reaction. Such migration and change process of ruthenium atoms is also highly probably caused by the dynamic reconstruction of catalyst during the ammonia decomposition reaction. Therefore, it is suggested that the in-situ transmission electron microscopy test of Ru/CeC under heated ammonia atmosphere should be supplied to intuitively prove the migration and change process of ruthenium atoms in ammonia, making the analysis more convincing.
4. The article claims that the catalyst is structurally stable after 100 hours of high-pressure testing, but the evidence provided is completely insufficient to support this key conclusion. The EXAFS fitting data of the sample after reaction must be provided, and the exact coordination number is used to prove that the Ru-Ru coordination has not increased. Otherwise, the conclusion of "structural preservation" is arbitrary. The Ru 3p XPS, Ce 3d XPS and Ru K-edge XANES data of the catalyst after the reaction must be supplemented and strictly compared with the data before the reaction.
5. Simply attributing the N 1s XPS peak (~398.7 eV) to "nitrogen incorporation into the CeO2 lattice" is hasty. This binding energy may also correspond to adsorbed nitrogen species (e.g. -NH2, -NH) or surface nitrides rather than bulk doping. The authors do not provide any higher-level spectroscopic evidence (such as N K-edge XANES) or structural evidence (such as changes in lattice parameters in XRD) to support the strong conclusion of "lattice doping".
6. The interpretation of the N 1s XPS peak should be more cautious, and it is recommended to change it to "nitrogen-containing species are preferentially incorporated/adsorbed on CeO2". It is strongly recommended to try to obtain N K-edge soft X-ray absorption spectrum, which can clearly distinguish the chemical environment of nitrogen (such as π bond, σ bond) and provide decisive evidence for the existence form of nitrogen. The DFT calculation only considers the adsorption of Ru-NH2. The adsorption energy of Ru atoms on the N-doped CeO2 surface should be additionally calculated and compared with the pure CeO2 surface to directly verify the hypothesis of "nitrogen doping guided anchoring".
7. The experimental conditions of the reaction series experiments (Supplementary Fig. 15b, c) are not clearly described. For example, when measuring the H2 reaction series, how do you keep the total flow rate constant while changing the H2 partial pressure? Is it balanced with Ar? This needs to be written clearly in the methods section or figure legend, otherwise the rigor of the experiment is called into question. The explanation for "mitigated hydrogen poisoning" is mainly based on the fact that the H2 reaction series is more negative. But this is only circumstantial evidence. If in situ DRIFTS can be performed to observe the coverage of adsorbed H species on different catalysts, or H2-TPD can be performed to compare the desorption

behavior of $H_2$, it will provide more direct experimental support for this conclusion. Specify the exact conditions for reaction series experiments. It is strongly recommended to supplement experiments such as $H_2$-TPD to strengthen the mechanism of resistance to hydrogen poisoning.
8. $H_2$-TPR (Fig. 2c): Only the peak temperature is discussed, but the quantitative analysis of the peak area is insufficient. The peak area is related to the number of reducible oxygen species. The peak area of Ru/CeC_NH3 is larger. Its association with more interface sites or defect sites should be discussed in depth instead of talking about "strong interactions" in general. Raman spectrum (Figure 2d): The assignment of peaks at ~690 and ~971 cm-1 (Ru-O-Ce) directly quoted from the literature, but did not consider the possible complex signals of the carrier itself (CeC). The Raman spectrum of the CeC_550 support needs to be provided as a baseline to prove that these peaks indeed originate from the introduction of Ru.
9. It should be mentioned that some of the conclusions drawn in this paper seem to be a little unconvincing. For example, it is slightly far-fetched to directly infer the migration kinetics of Ru from $CH_4$ generation experiments (Fig. 5a-c). The limitations of this circumstantial evidence need to be expressed more carefully.
10. It is observed that an apparent discrepancy in Figure 4c, where the ammonia decomposition rates at 450 °C appear to be higher than those at 500 °C for the catalyst, which is not match the ammonia decomposition performance test mentioned in Fig 4a. This seems counterintuitive, as reaction rates typically increase with temperature. Could the authors please verify if the temperature labels for the long-term stability tests are correct?
11. Grammatical errors and unclear expressions should be carefully checked and corrected.

Reviewer #4

(Remarks to the Author)
I co-reviewed this manuscript with one of the reviewers who provided the listed reports. This is part of the Nature Communications initiative to facilitate training in peer review and to provide appropriate recognition for Early Career Researchers who co-review manuscripts.

Version 1:

Reviewer comments:

Reviewer #1

(Remarks to the Author)
The revised paper can be accepted in the present form

Reviewer #2

(Remarks to the Author)
The authors have modified the manuscript with care, done some additional experiments and responded to the reviewers well. I recommend accepting it.

Reviewer #3

(Remarks to the Author)
I thank the author for their careful response, which addressed all my initial concerns, and I am satisfied with the current version.

Reviewer #4

(Remarks to the Author)
I co-reviewed this manuscript with one of the reviewers who provided the listed reports. This is part of the Nature Communications initiative to facilitate training in peer review and to provide appropriate recognition for Early Career Researchers who co-review manuscripts.

**Open Access** This Peer Review File is licensed under a Creative Commons Attribution 4.0 International License, which permits use, sharing, adaptation, distribution and reproduction in any medium or format, as long as you give appropriate credit to the original author(s) and the source, provide a link to the Creative Commons license, and indicate if changes were made.
In cases where reviewers are anonymous, credit should be given to 'Anonymous Referee' and the source.
The images or other third party material in this Peer Review File are included in the article's Creative Commons license, unless indicated otherwise in a credit line to the material. If material is not included in the article's Creative Commons license and your intended use is not permitted by statutory regulation or exceeds the permitted use, you will need to obtain permission directly from the copyright holder.
To view a copy of this license, visit https://creativecommons.org/licenses/by/4.0/

**[Response to Reviewers]**

We deeply appreciate the reviewers for their kind comments which were very helpful to improve our manuscript. Their comments were answered point by point as shown below.

**Reviewer: 1**

*In the present work Ru on CeC together with different $RuCeO_2$ and RuC catalysts were prepared and tested in the Ammonia Decomposition. The catalysts were extensively characterized by different techniques including TEM, EXAFS and XPS. In general the paper is well written, and the experimental data support the discussion. The main conclusion of this paper is that $NH_3$ induces the selective migration of ruthenium (Ru) atoms onto $CeO_2$, forming a durable system. My impression is that this paper might be suitable for publication after addressing the following points*

*1. In the introduction the choice of ceria-coated carbon support as support should be better motivated*

**Answer)**

We sincerely thank the reviewer for this valuable comment. We agree that the rationale for selecting a ceria-coated carbon support ($CeO_2$-C) was not sufficiently articulated in the original version of the introduction. To address this concern, we have revised the final paragraph of the Introduction to more clearly explain the design logic of the support. Specifically, we clarified that the ceria-carbon architecture was intentionally constructed by integrating bottom-up and top-down concepts. While our previous text emphasized the ammonia-induced top-down restructuring, we now also describe the bottom-up design principle underlying the support selection.

Ceria was chosen due to its redox flexibility ($Ce^{3+}$/$Ce^{4+}$), oxygen vacancy formation ability, and strong metal-support interactions. However, we recognized that these properties are strongly dependent on particle size and crystallinity and are not achieved in bulk ceria. Therefore, an amorphous carbon substrate with high surface area was employed as a dispersive framework to control ceria domain size, crystallinity, and defect density through annealing. This approach allows tuning of surface redox characteristics that are beneficial both for metal anchoring and catalytic performance.

Accordingly, the Introduction has been revised to reflect this integrated design strategy. The updated paragraphs are now as follows.

**[Revised manuscript, page 5]**

In this study, we integrated key elements of both bottom-up and top-down strategies to design a catalyst whose interfacial structure can be established and maintained under reaction conditions. Ceria was selected as the oxide support component due to its well-known redox flexibility ($Ce^{3+}$ / $Ce^{4+}$), ability to form oxygen vacancies, and to have strong metal-support interactions that can stabilize dispersed metal species. However, the intrinsic advantages are

not often fully realized when bulk ceria is used directly, as its redox properties and defect structure are strongly influenced by particle size and crystallinity[34,35]. One effective approach to modulate these properties is to control ceria domain size and structural order, which directly affect surface reducibility and vacancy concentration. To achieve this, we employed a high-surface-area carbon substrate as a dispersive framework for ceria domains. By controlling the annealing temperature, crystallinity, domain size, and surface defect density of ceria were tuned, enabling optimization of its surface redox characteristics. Ruthenium was deposited on the optimized support, and ammonia served as a chemically active nitrogen source to induce atomic-scale restructuring. Unlike conventional top-down approaches, reactive nitrogen intermediates generated during ammonia decomposition drove the selective migration of Ru toward $CeO_2$ domains under reaction conditions. This reaction-coupled restructuring not only enabled redistribution but also stabilized a catalytically favorable Ru-$CeO_2$ interface. In contrast, hydrogen treatment produced randomly dispersed Ru nanoparticles. The resulting Ru-$CeO_2$ configuration suppressed hydrogen poisoning and improved compatibility with ammonia decomposition, leading to high hydrogen production under high-pressure conditions while maintaining structural stability after 100 hours of continuous operation.

*2. Pag 7 line 137 "After the $NH_3$ treatment, most of the ruthenium located on the carbon disappeared…) Figure 1b is not enough to demonstrate that Ru is present only on $CeO_2$. I still see Ru species on carbon. Moreover, the real content of Ru after the treatment should be shown and better discussed.*

**Answer)**

We sincerely thank the reviewer for this careful observation. We agree that the **original Fig. 1b** alone is not sufficient to statistically demonstrate the redistribution of Ru species after ammonia treatment, nor to exclude the presence of residual Ru on carbon. To provide more quantitative evidence, we performed line-scan EDS analysis across multiple regions of the samples. These include (i) carbon-only regions, (ii) carbon-ceria boundary regions, and (iii) ceria domains. The corresponding results are provided in **Fig. R1-R3**.

For Ru/$CeO_2$-C_$NH_3$, the Ru and Ce signals predominantly overlapped in ceria domains (**Fig. R2**), indicating preferential interaction of Ru with $CeO_2$. In contrast, for Ru/$CeO_2$-C_$N_2$ and Ru/$CeO_2$-C_$H_2$, distinct Ru signals were observed in the regions without corresponding Ce signals (**Fig. R1** and **Fig. R3**), suggesting the presence of Ru species on carbon. To further address the reviewer's concern regarding bright spots observed on carbon in the ammonia treated sample, we performed additional line EDS analysis specifically on these regions (**Fig. R2c**). In these areas, both Ru and Ce signals were detected. However, the net intensities were significantly lower compared to those measured on well-defined ceria domains. We also note that Ce (Z=58) provides stronger Z-contrast than Ru (Z=44), and therefore bright dots on carbon regions may originate from trace Ce species or overlapping signals rather than exclusively from Ru atoms. Based on the analysis, we have written the manuscript text to clarify that "most of the ruthenium located on the carbon disappeared and selectively migrated toward $CeO_2$ domains," rather than implying complete removal. We also added that trace amount of Ru (and Ce) remains on the carbon substrate to improve transparency.

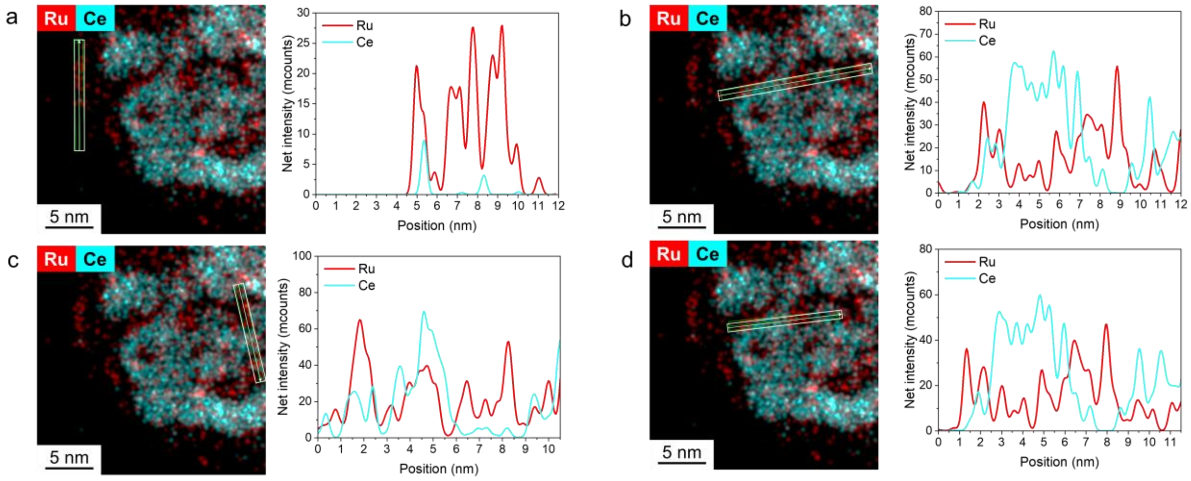


**Fig. R1** Line EDS mapping results for $Ru/CeO_2$-C_$N_2$.

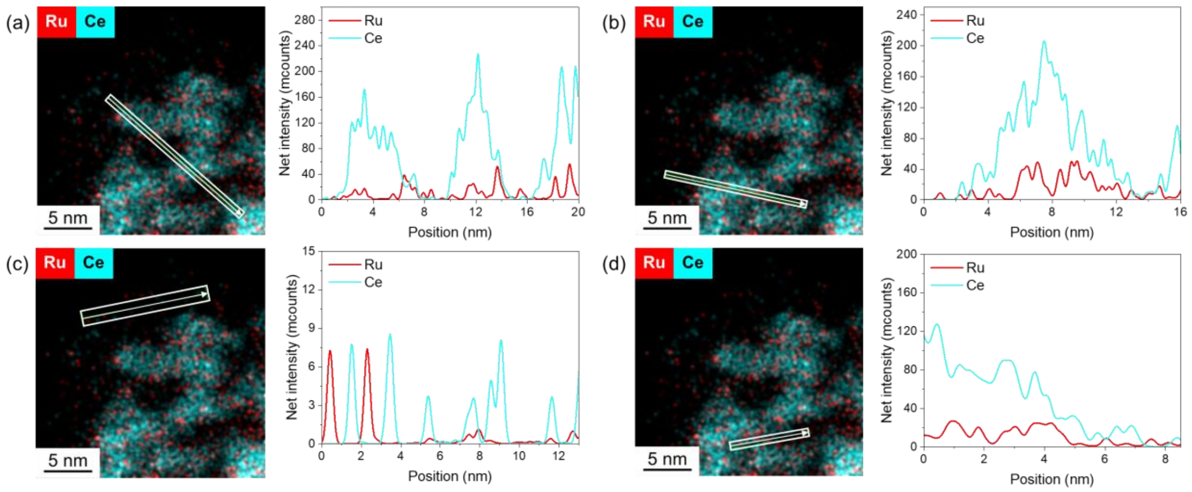


**Fig. R2** Line EDS mapping results for $Ru/CeO_2$-C_$NH_3$.

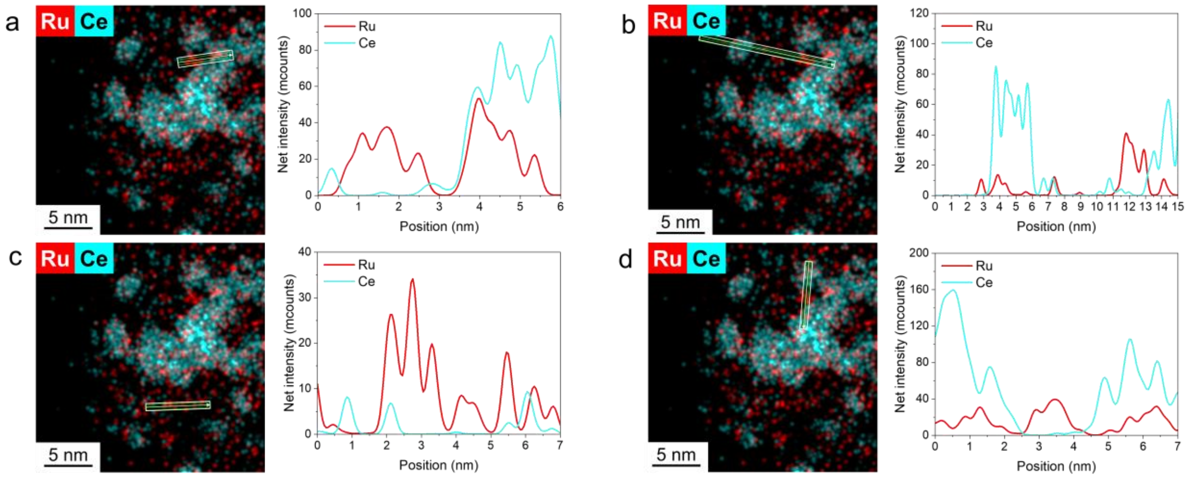


**Fig. R3** Line EDS mapping results for $Ru/CeO_2$-C_$H_2$.

Finally, to verify that ammonia treatment did not cause loss of Ru content, we conducted ICP-

OES analysis for Ru/$CeO_2$-C_$N_2$, Ru/$CeO_2$-C_$H_2$, and Ru/$CeO_2$-C_$NH_3$. The $CeO_2$ content was measured within a narrow range of 62.4 - 66.6 wt %, and the Ru loading remained consistently at ~1.6 wt % across all samples (**Table R1**). These results confirmed that the observed redistribution is due to migration rather than metal loss during treatment. We believe that these additional EDS analyses and ICP data substantially strengthened the evidence for selective Ru migration toward $CeO_2$ domains while maintaining overall metal loading. **Fig. R1-3** were included as **Supplementary Fig. 6-8**. **Table R1** was substituted with **Table S1** in original manuscript.

**Table R1.** ICP-OES results of Ru/$CeO_2$-C_$N_2$, Ru/$CeO_2$-C_$H_2$, and Ru/$CeO_2$-C_$NH_3$.

| Sample | Ru content (wt %) | $CeO_2$ content (wt %) | Surface area ($m^2$/g) |
|---|---|---|---|
| Ru/$CeO_2$-C_$N_2$ | 1.6 | 63.0 | 321.1 |
| Ru/$CeO_2$-C_$H_2$ | 1.6 | 62.4 | 280.6 |
| Ru/$CeO_2$-C_$NH_3$ | 1.6 | 66.6 | 283.1 |
| Ru/$CeO_2$ | 1.8 | - | 31.0 |
| Ru/C | 1.7 | - | 816.3 |

**[Revised manuscript, page 7]**

The spatial distribution and local structure of ruthenium were analyzed using ultrahigh-resolution double Cs-corrected TEM (UHR-TEM) with multichannel energy-dispersive X-ray spectroscopy (multi-EDS). The ruthenium species in Ru/$CeO_2$-C_$N_2$ were randomly distributed over both the $CeO_2$ and carbon domains. They existed either in an atomically dispersed form or as nanoparticles (Fig. 1a and Supplementary Fig. 6). After the $NH_3$ treatment, most of the ruthenium located on the carbon disappeared, and ruthenium selectively migrated toward the $CeO_2$ domains. Only a trace amount of Ru was still detected on the carbon regions together with Ce signals, although the corresponding intensities were significantly lower than those observed on ceria domains (Fig. 1b and Supplementary Fig. 7). In contrast, the $H_2$ treatment formed randomly located Ru nanoparticles on both carbon and $CeO_2$, with mixed sizes (Fig. 1c and Supplementary Fig. 8).

**Reviewer: 2**

*In this manuscript, the authors prepared a catalyst for high-pressure ammonia decomposition reaction by constructing small-sized $CeO_2$ nanoparticles on a carbon support and loading Ru. The authors found that $NH_3$ treatment of the "Ru/CeC" catalyst induced a selective atomic rearrangement of Ru atoms on the $CeO_2$ nanoparticles, resulting in the formation of well-defined and stable Ru single atoms on the $CeO_2$ surface ("Ru/CeC_NH3"). This phenomenon is very interesting, but the current data provided in the manuscript are insufficient to support it, and there are several important problems that need to be discussed. Therefore, I believe this manuscript requires major revision.*

*1. Regarding the choice of the reaction system. According to the reaction equation $2NH_3 = 3H_2 + N_2$, low pressure favors the ammonia decomposition reaction. Why test reactions under high pressure? If it is due to practical process requirements, what is the cost assessment? Moreover, the scientific investigation of the Ru state in this manuscript did not employ high-pressure $NH_3$ conditions, and research on the Ru state under high-pressure $NH_3$ conditions is lacking.*

**Answer)**

We thank the reviewer for this important comment. While it is thermodynamically true that the decomposition of ammonia is favored at low pressure due to the increase in gas moles, operating the reaction under high pressure is a strategic choice driven by the overall efficiency of the hydrogen production chain. While many fundamental studies on ammonia decomposition are performed under atmospheric pressure to maximize conversion, practical processes are often designed to operate under pressurized conditions to facilitate downstream hydrogen purification. In this context, investigating catalytic behavior under elevated pressure can provide complementary insight into catalyst performance under application-relevant environments. In practical ammonia cracking systems, as illustrated in the **Fig. R1**, the reactor effluent typically consists of a mixture of $H_2$, $N_2$, and residual unreacted $NH_3$.

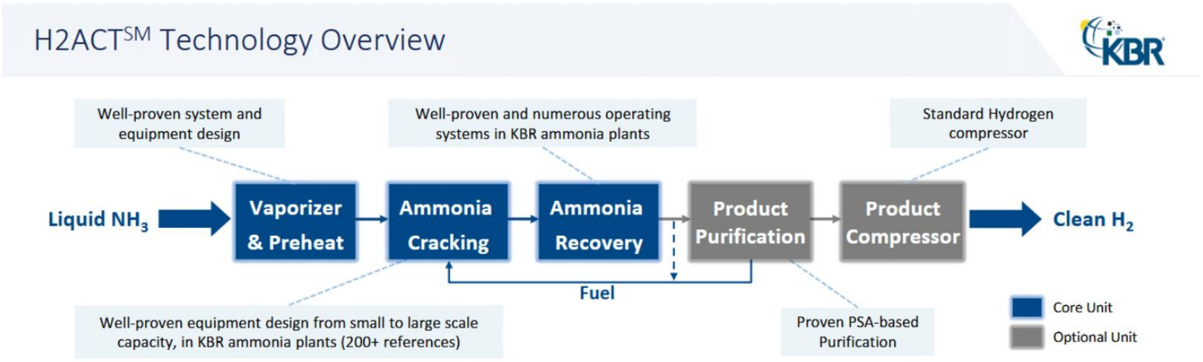


**Fig. R1** Schematic illustration of the KBR H2ACT$^{TM}$ ammonia cracking process integrated with PSA-based hydrogen purification[1].

The remaining ammonia is first removed through a condensation of scrubbing step, after which the resulting $H_2/N_2$ gas mixture is purified using pressure swing adsorption (PSA) to obtain high-purity hydrogen. Since PSA processes operate efficiently with pressurized feed streams (9~10 bar), performing ammonia cracking under elevated pressure enables direct integration with downstream PSA units. In contrast, when ammonia decomposition is conducted at low pressure, an additional $H_2/N_2$ compression step to 10 bar is required prior to PSA purification.

Hydrogen compression is widely recognized as an energy-intensive process, typically requiring on the order of 1-7 kWh/$kg_{H2}$ depending on the target pressure and compressor configuration[2]. This additional step increases both capital expenditure (e.g., compressors and intercoolers) and operational energy consumption, thereby raising the overall cost of hydrogen production. Based on the NREL compression model reported by Amos[3], compression to 200 bar requires approximately 2.2 kWh/$kg_{H2}$. Although direct literature values for relatively low pressures (e.g., ~10 bar) are limited, the required compression work can be estimated assuming a logarithmic dependence on the pressure ratio. Using this scaling relationship, compressing hydrogen from 1 bar to 10 bar requires approximately ~1 kWh/$kg_{H2}$, which corresponds to roughly 3 % of the lower heating value (LHV) of hydrogen. Such parasitic energy consumption represents a non-negligible process penalty and can reduce the overall efficiency of ammonia-to-hydrogen conversion systems. For example, on this basis, the additional cost associated with low-pressure cracking followed by hydrogen compression is estimated to be on the order of ~ 0.1-0.3 USD/$kg_{H2}$, depending on plant scale and electricity price. This corresponds to a meaningful increase in hydrogen production costs.

In addition, hydrogen compressors represent a substantial capital expenditure (CAPEX) in hydrogen processing systems. The installed cost of hydrogen compressors is typically estimated to be in the range of approximately 800-1500 USD/kW[4], depending on compressor type and operating pressure. This range reflects the economies of scale observed in MW-scale systems, where specific costs tend to converge compared with the higher unit costs (> 3000 USD/kW) for small-scale compressors below 100 kW. The electricity required for hydrogen compression also contributes directly to operational expenditure (OPEX), further increasing the overall cost of hydrogen production. Therefore, operating the ammonia cracking reactor under elevated pressure can eliminate the need for intermediate hydrogen compression prior to PSA.

Although pressurized operation may require somewhat higher reaction temperatures to compensate for reduced ammonia conversion, the associated thermal penalty is comparatively small. A simple heat-capacity estimate suggests that increasing the reactor temperature by 100 °C corresponds to an additional energy input of approximately 0.4 - 0.5 kWh/$kg_{H2}$, which remains lower than the typical energy requirement associated with hydrogen compression. Importantly, it should be noted that the experimental results obtained in this study show only a negligible difference in ammonia conversion between atmospheric and pressurized conditions. As shown in **Fig. R2** (**Fig. 4 in the original manuscript**), the catalytic performance of $Ru/CeO_2$-C_$NH_3$ under 9 bar is comparable to that observed at 1 bar across the investigated temperature range with long term high-pressure operation. This behavior is particularly advantageous from a process perspective because it indicates that pressurized operation can be implemented without a significant loss in catalytic activity, thereby enabling improved process

integration with downstream hydrogen purification while maintaining high ammonia conversion.

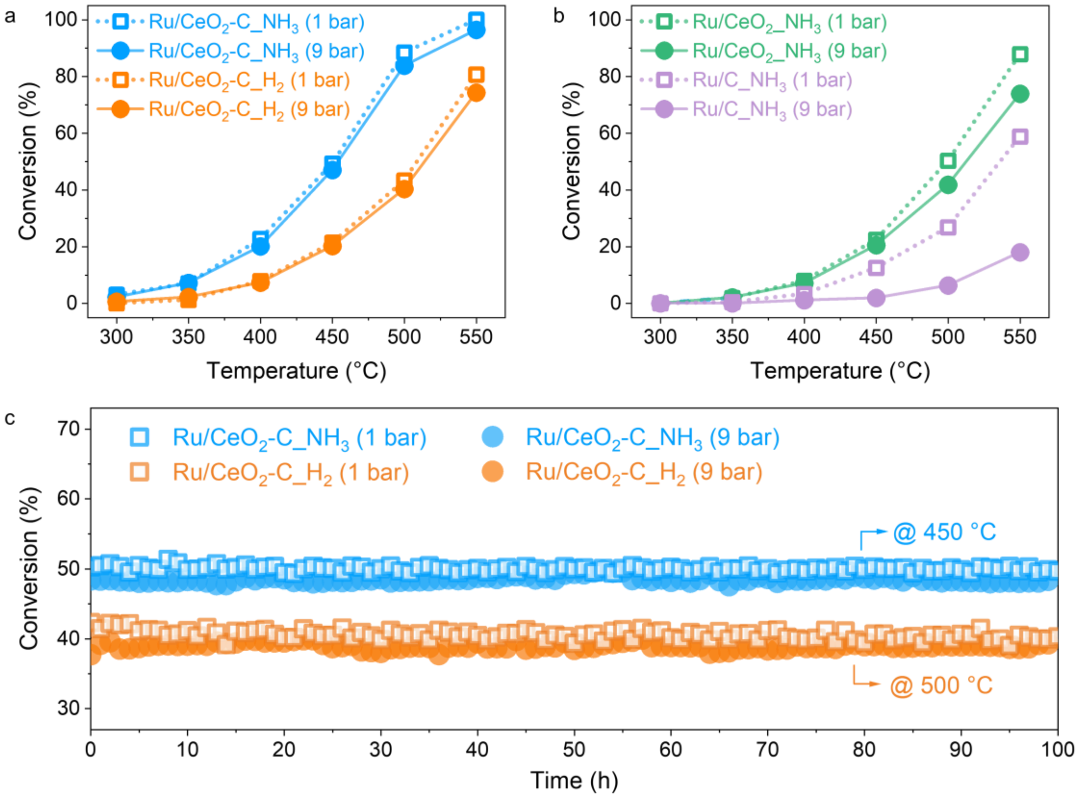


**Fig. R2 (Fig. 4 in original manuscript)** $NH_3$ conversion results for (a) Ru/$CeO_2$-C_$NH_3$, Ru/$CeO_2$-C_$H_2$, (b) Ru/$CeO_2$, and Ru/C at 1 and 9 bar. (c) Long-term stability test of Ru/$CeO_2$-C_$NH_3$ and Ru/$CeO_2$-C_$H_2$ for 100 h at 1 and 9 bar. The long-term reaction was conducted at 450 and 500 °C for Ru/$CeO_2$-C_$NH_3$ and Ru/$CeO_2$-C_$H_2$, respectively. $NH_3$ decomposition was conducted using 100% $NH_3$ with a WHSV of 30,000 mL/$g_{cat}$·h.

Secondly, we agree with the reviewer that investigating the Ru state under high-pressure $NH_3$ conditions would provide valuable insight into the catalyst structure under practical reaction environments. However, in-situ characterizations under pressurized ammonia environments remain experimentally challenging due to the corrosive and toxic nature of ammonia, particularly under elevated pressure. In this work, we therefore made every effort to systematically probe the structural evolution of Ru species with characterizations after high-pressure reactions. Nevertheless, following the reviewer's suggestion, we performed additional catalytic experiments under pressurized conditions within the capabilities of our laboratory. This includes activation energy measurements and reaction order determination of $NH_3$ and $H_2$ in pressurized conditions (**Fig. R3**)

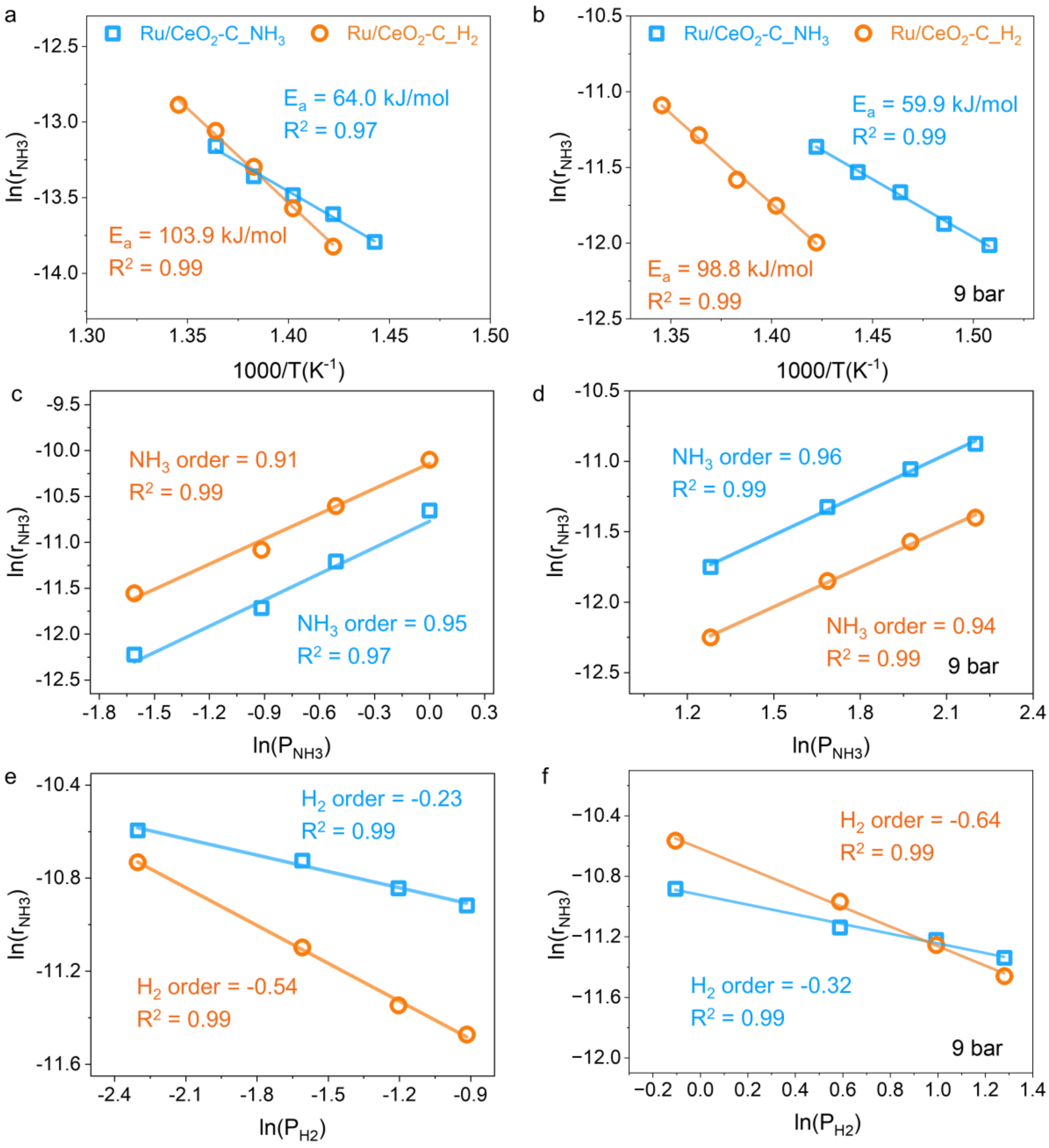


**Fig. R3** (a, b) Activation energy, (c, d) reaction orders with respect to $NH_3$ partial pressure, and (e, f) reaction orders with respect to $H_2$ partial pressure of Ru/$CeO_2$-C_$NH_3$ and Ru/$CeO_2$-C_$H_2$. These experiments were performed at ambient pressure. (a, c, e) were obtained under ambient pressure, while (b, d, f) were measured under elevated pressure (9 bar).

Activation energy obtained under pressurized conditions (9 bar) was slightly lower than that measured at ambient pressure, indicating increased availability of ammonia at elevated pressure. The reaction order with respect to $NH_3$ remained close to 1 under both pressure conditions, indicating that the dependence of the reaction rate on ammonia concentration was largely preserved. In contrast, a slight decrease in the reaction order with respect to $H_2$ was observed under pressurized conditions, indicating that hydrogen inhibition became less pronounced under elevated pressure. More importantly, the intrinsic differences between the

catalysts were consistently maintained across pressure conditions. Ru/$CeO_2$-C_$NH_3$ exhibited a lower activation energy and a higher reaction order with respect to $H_2$ compared to Ru/$CeO_2$-C_$H_2$, indicating weaker hydrogen inhibition on the former catalyst. These trends were observed under both ambient and pressurized conditions.

Overall, these additional results provided direct experimental evidence that the key kinetic trends remain valid under pressurized conditions. While the differences between ambient and elevated pressure were not pronounced, the preservation of activation energy trends and reaction orders confirmed that the intrinsic catalytic behaviors were maintained. This supports the robustness of the proposed structure-performance relationship and highlights the relevance of the present catalyst system under practically relevant pressurized operation. **Fig. R3** was added as **Supplementary Fig. 26**, and corresponding manuscript was revised.

**[References]**


1 Stylianou, E. H2ACT: Empowering Energy Mobility through Large Scale Ammonia Cracking. *Ammonia Energy Association Annual Conference* (2024). https://ammoniaenergy.org/presentations/h2act-empowering-energy-mobility-through-large-scale-ammonia-cracking/

2 Bossel, U. Does a hydrogen economy make sense? *Proc. IEEE 94*, 1826–1837 (2006).

3 Amos, W. A. Costs of storing and transporting hydrogen. *NREL/TP-570-25106* (1998).

4 James, B. D., Baum, G. N., Perez, J. & Baum, K. N. Techno-economic analysis of hydrogen compression, storage, and dispensing systems. *NREL Technical Report NREL/TP-5400-58564* (2013).


**[Revised manuscript, page 17]**

Ammonia decomposition at high pressure represents a key step in realizing efficient hydrogen production and storage, yet catalyst stability remains a major challenge under such harsh conditions. Ammonia is a promising hydrogen carrier because of its high hydrogen content, carbon-free composition, and ease of liquefied storage and transport[52-54]. Hydrogen extraction from ammonia typically requires thermal decomposition above 450 °C[55-57]. Especially, high-pressure operation is favored for practical applications. Although ammonia decomposition is thermodynamically favored at low pressure, high-pressure operation is advantageous from a process perspective as it enables direct integration with downstream hydrogen purification systems such as pressure swing adsorption (PSA)[58-60]. In low-pressure systems, an additional compression step is required prior to purification, which is energy-intensive and increases both capital and operational costs[61,62]. Hydrogen compression from 1 to 10 bar typically requires on the order of ~1 kWh/$kg_{H2}$, corresponding to a non-negligible energy penalty and additional cost[63]. Operating the reactor under elevated pressure eliminates the need for recompression, thereby improving overall process efficiency despite a modest thermal penalty associated with slightly higher reaction temperatures.

**[Revised manuscript, page 18]**

To understand the origin of this enhanced performance, kinetic and mechanistic analyses were conducted in both ambient and pressurized conditions, based on the activation energies and reaction orders. The activation energy determined from the Arrhenius plots showed that Ru/$CeO_2$-C_$NH_3$ exhibited a lower activation barrier of 64.0 kJ/mol (1 bar) and 59.9 kJ/mol (9 bar), as compared to 103.9 kJ/mol (1 bar) and 98.8 kJ/mol (9 bar) for Ru/$CeO_2$-C_$H_2$. This indicates that $NH_3$ activation occurred more easily over the Ru/$CeO_2$-C_$NH_3$ (Supplementary Fig. 26a, b). The reaction order was determined by varying the partial pressures of $H_2$ and $NH_3$. The order with respect to $NH_3$ was 0.95 (1 bar) and 0.96 (9 bar) for the Ru/$CeO_2$-C_$NH_3$, 0.91 (1 bar) and 0.94 (9 bar) for the Ru/$CeO_2$-C_$H_2$, suggesting that $NH_3$ activation proceeded through a similar kinetic regime for both catalysts (Supplementary Fig. 26c, d). In contrast, the reaction order with respect to hydrogen was -0.54 (1 bar), and -0.64 (9 bar) for Ru/$CeO_2$-C_$H_2$, -0.23 (1 bar), and -0.32 (9 bar) for Ru/$CeO_2$-C_$NH_3$, confirming that the Ru/$CeO_2$-C_$NH_3$ had less hydrogen poisoning on Ru sites (Supplementary Fig. 26e, f). These results indicate that the selectively formed Ru-$CeO_2$ interfaces via $NH_3$ treatment facilitated efficient $NH_3$ activation while mitigating hydrogen inhibition, thereby accelerating the overall decomposition process. As the differences between ambient and elevated pressures were not pronounced, the consistent activation energy trends and reaction orders confirmed that the intrinsic catalytic behavior is maintained.

*2. In terms of catalytic performance, the current catalytic activity achieved with 1.6 wt% Ru loading is not very competitive and is lower than that of most low-loading Ru-based catalysts using rare-earth oxides as supports.*

**Answer)**

We appreciate the reviewer's comment regarding the catalytic performance. It should be noted that most Ru-based ammonia decomposition catalysts reported in the literature are typically evaluated under atmospheric pressure conditions. As discussed in our response to "Reviewer Comment 1", practical ammonia cracking systems are often designed to operate under elevated pressure in order to facilitate integration with downstream hydrogen purification processes such as PSA. Therefore, catalytic performance evaluated under pressurized conditions is particularly relevant from a practical process perspective.

In this study, the catalyst was intentionally designed to operate under pressurized conditions. Because ammonia decomposition becomes thermodynamically less favorable at elevated pressure, a slightly higher Ru loading (1.6 wt%) was adopted to ensure sufficient catalytic activity under these conditions. Despite this moderate Ru loading, the catalyst exhibits competitive performance under pressurized operation. Moreover, relatively few studies have reported ammonia decomposition performance under elevated pressure conditions. When compared under similar pressurized conditions, the catalytic activity observed in this work shows competitive performance as shown in **Supplementary Table 4.** Importantly, the catalytic activity in this work does not deteriorate under 9 bar operation, demonstrating robustness under thermodynamically unfavorable conditions. This highlights that the catalyst is not only active, but also practically relevant for integration into pressurized ammonia

cracking systems. Therefore, while the catalytic activity may appear less competitive when compared directly with atmospheric-pressure studies, under practically relevant pressurized conditions, the catalyst developed in this study shows competitive, and in some cases superior, performance relative to existing reports.

Importantly, the primary objective of this study is not solely to maximize catalytic activity but to elucidate the reaction-driven structural evolution of Ru species during ammonia decomposition. We demonstrate that ammonia acts as a chemically active nitrogen source that promotes the migration of Ru atoms onto $CeO_2$ domains supported on carbon, leading to the in-situ formation of atomically dispersed Ru-$CeO_2$ interfacial sites. This reaction-driven restructuring enhances the redox properties of ceria and contributes to stable catalytic performance for ammonia decomposition under pressurized conditions.

*3. Regarding the naming of the catalyst, "CeC" can easily be mistaken for cerium carbide ($CeC_2$). It is recommended to change it to another naming convention.*

**Answer)**

We thank the reviewer for this important comment. We agree that the abbreviation "CeC" could be confused with cerium carbide ($CeC_2$), potentially leading to misunderstanding regarding the chemical nature of the support. To eliminate this ambiguity, we have revised the catalyst naming throughout the manuscript. Specifically, the abbreviations Ru/CeC_$N_2$, Ru/CeC_$H_2$, and Ru/CeC_$NH_3$ have been changed to Ru/$CeO_2$-C_$N_2$, Ru/$CeO_2$-C_$H_2$, and Ru/$CeO_2$-C_$NH_3$, respectively. This revised naming indicates that the catalyst consists of ceria ($CeO_2$) dispersed on carbon, rather than forming a cerium carbide phase. The updated naming has been applied consistently in the main text, figures, captions, and Supplementary Information to ensure clarity and avoid potential confusion.

*4. Based on the characterization results, $NH_3$ treatment enables Ru species to disperse more on $CeO_2$ and remain highly dispersed. However, I believe it is currently not justified to claim it as single atoms, and it cannot be confirmed that Ru single atoms can remain stable under high-temperature ammonia decomposition conditions, as there is no in situ characterization to support this.*

**Answer)**

We thank the reviewer for this important comment regarding the structural assignment of Ru species and their stability under ammonia decomposition conditions. We agree that while the present characterization results clearly indicate a highly dispersed Ru structure, the current level of evidence may not be sufficient to clearly justify the use of the term "single atoms". In this work, the dispersion of Ru species was determined based on the convergence of multiple complementary analysis, including ultrahigh-resolution HAADF-STEM, multi-EDS mapping, XAFS, and Raman spectra. These results consistently indicated that Ru species on Ru/$CeO_2$-C_$NH_3$ were atomically dispersed, predominantly stabilized on $CeO_2$ domains, and did not form detectable nanoparticles. Nevertheless, we acknowledge that the term "single atoms" may be considered overly specific in the absence of direct identification of individual Ru atoms.

Therefore, to more precisely reflect the level over structural evidence obtained in this study, we have revised the description throughout the manuscript to "atomically dispersed Ru species on $CeO_2$ domains". This refinement reflects a more precise description of the experimentally supported structure. Also, we measured additional characterizations to clarify Ru structure before and after ammonia decomposition.

First of all, we carefully re-examined the catalyst structure using ultrahigh-resolution HAADF-STEM imaging at the highest attainable magnification. The updated images, including additional high-resolution data at the ~2 nm scale, have been incorporated into the revised manuscript. Under these imaging conditions, the $CeO_2$ lattice was clearly resolved with high crystallinity, confirming that atomic-scale structural features of the support could be reliably observed. Importantly, no Ru nanoparticles or sub-nanometer clusters were observed on the $CeO_2$ surface in the Ru/$CeO_2$-C_$NH_3$. If Ru were present as nanoparticles or small clusters, they would appear as distinct high-contrast features separated from the ceria lattice (**Supplementary Fig. 9**). Instead, only isolated bright features were observed in close association with the $CeO_2$ domains. To guide interpretation, representative features for atomically dispersed Ru species have been highlighted with yellow circles in the revised UHR-TEM images (**Fig. R4**).

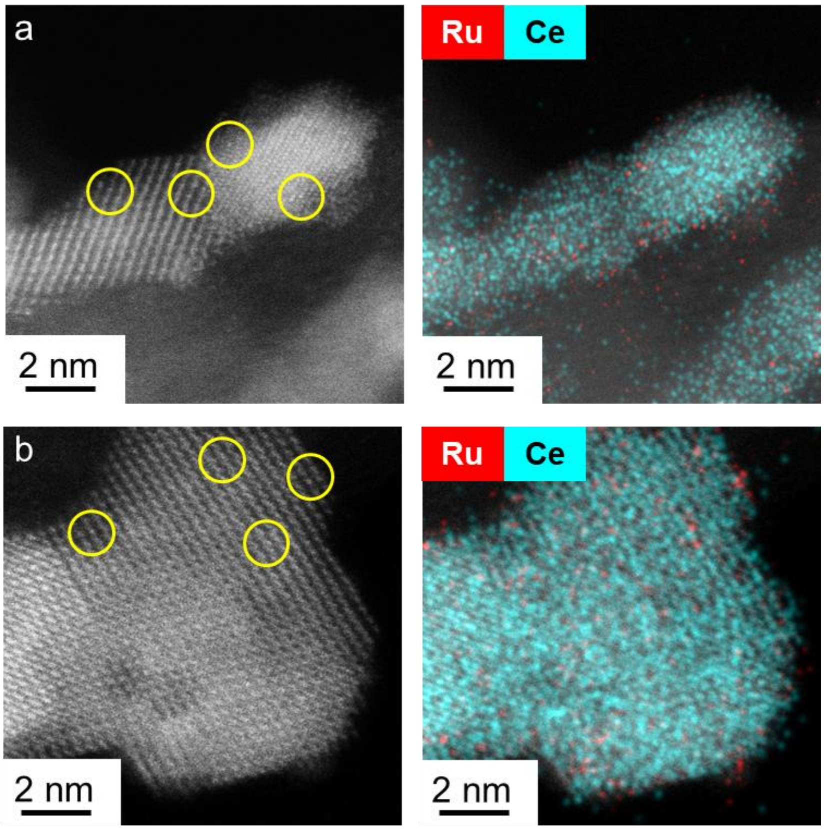


**Fig. R4** UHR-STEM images of Ru/$CeO_2$-C_$NH_3$ in 2 nm scale resolution. Sites for atomic Ru were highlighted with yellow circles.

We note that direct identification of individual Ru atoms on $CeO_2$ using HAADF-STEM is intrinsically challenging due to the limited Z-contrast difference between Ru (Z = 44) and Ce

(Z = 58). Because HAADF intensity scales approximately with atomic number, the heavier cerium atoms in the ceria lattice generate significantly stronger contrast than Ru. As a result, isolated Ru atoms on $CeO_2$ could appear only as subtle bright features within the already bright ceria lattice, making their identification considerably more difficult than in systems where metals are supported on lighter substrates. **Fig. R4** was added in revised manuscript as **Supplementary Fig. 10**.

To further support the spatial distribution of Ru species, we performed additional line-scan EDS analysis across multiple regions, including $CeO_2$ domains, carbon regions, and interfacial areas (**Fig. R5-7**). The results consistently showed that the Ru signal was strongly correlated with the Ce signal and predominantly localized within the ceria domains, while negligible Ru signal was detected in pure carbon regions. These results provide quantitative support for the preferential association of Ru species with $CeO_2$ domains after $NH_3$ treatment. **Fig. R5-7** were added in revised manuscript as **Supplementary Fig. 6-8**.

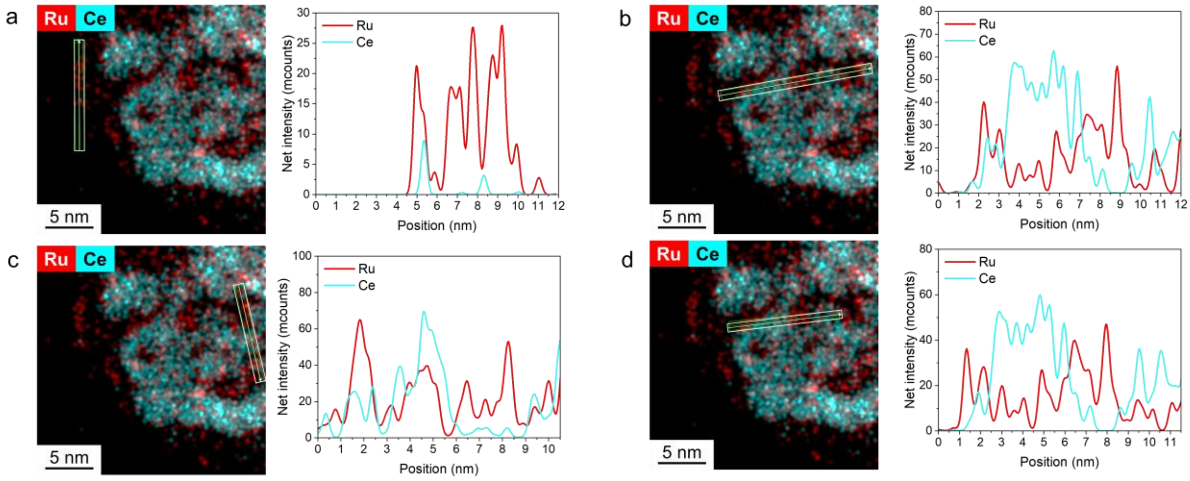


**Fig. R1** Line EDS mapping results for Ru/$CeO_2$-C_$N_2$.

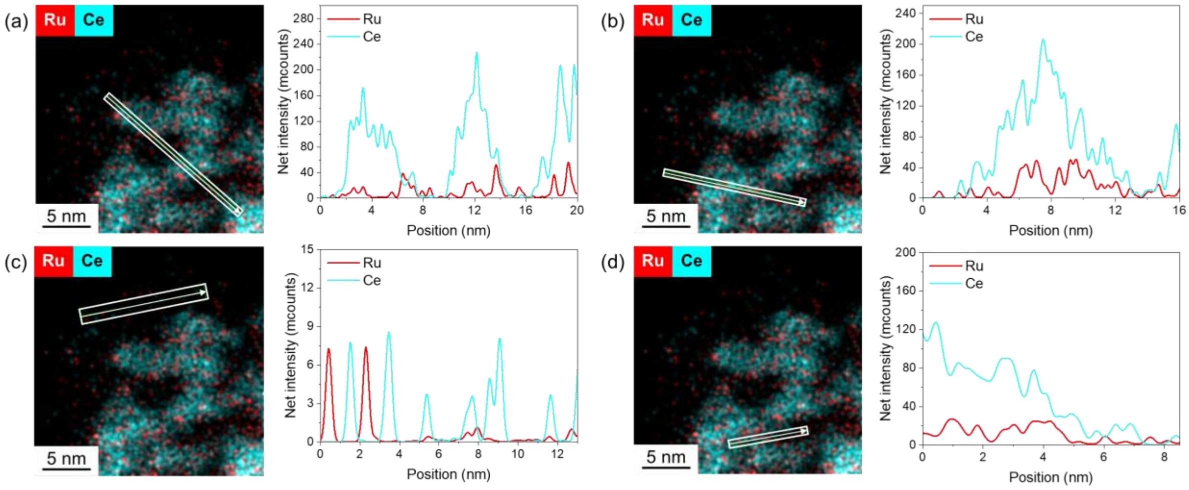


**Fig. R2** Line EDS mapping results for Ru/$CeO_2$-C_$NH_3$.

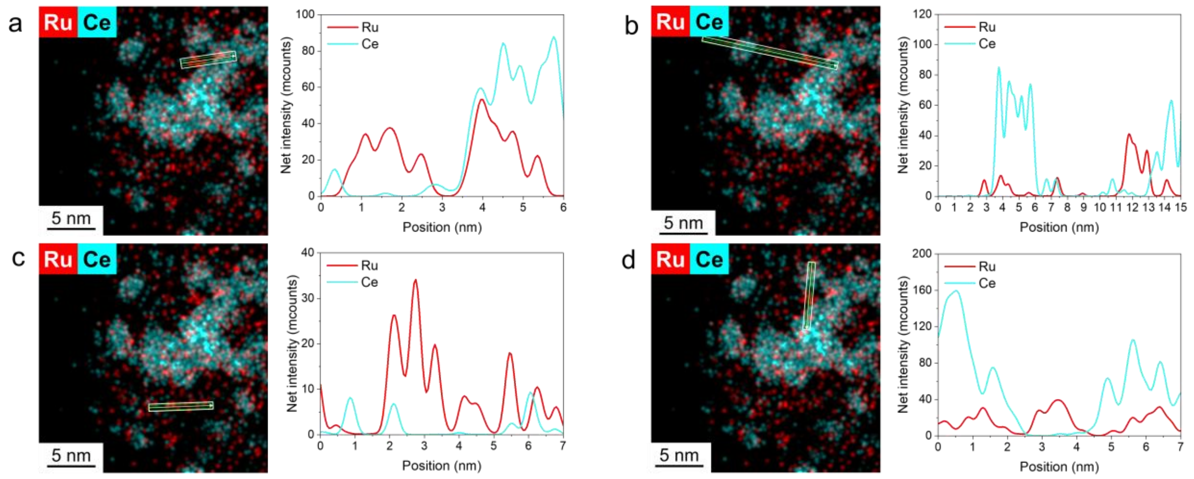


**Fig. R3** Line EDS mapping results for $Ru/CeO_2$-C_$H_2$.

In addition to electron microscopy analysis, XAFS provided complementary, element-specific structural information. EXAFS fitting of the $Ru/CeO_2$-C_$NH_3$ showed that Ru-O coordination was dominant (3.7), while the Ru-Ru coordination number was very low (0.3), indicating the absence of metallic Ru particles. This result was fully consistent with the microscopy observations and supports the presence of highly dispersed Ru species on the oxide support (**Fig. 2a, Supplementary Fig. 12,** and **Supplementary Table 2**).

We also acknowledge that in-situ characterization under ammonia decomposition conditions would provide direct insight into the structural evolution of Ru species. However, such measurements were experimentally constrained due to the corrosive and toxic nature of $NH_3$, particularly under high-temperature and pressurized conditions, which limits the applicability of techniques such as in-situ TEM or XAFS under these environments. To address the stability of the Ru structure under reaction conditions, we performed extensive post-reaction characterizations after prolonged ammonia decomposition. Notably, the catalysts were subjected to 100 hours of reaction under high-pressure conditions (9 bar), representing a severe and application-relevant environment. After the reaction, UHR-TEM and EDS mapping of the catalyst showed that Ru species remained highly dispersed on $CeO_2$ domains, without nanoparticle formation or aggregation (**Supplementary Figure 29**).

More importantly, EXAFS analysis of the $Ru/CeO_2$-C_$NH_3$ after durability test showed that the Ru-O coordination remains high (3.9), while the Ru-Ru coordination remains negligible (0.2), indicating that no significant aggregation of Ru species occurs during reaction. In contrast, the $Ru/CeO_2$-C_$H_2$ after durability test exhibited a substantially higher Ru-Ru coordination (1.4), consistent with the presence of Ru nanoparticles. These results demonstrated that the atomic Ru structure formed after $NH_3$ treatment was preserved even under prolonged high-pressure ammonia decomposition (**Fig. R8** and **Table R1**). Furthermore, Ru K-edge XANES spectra showed negligible changes before and after reaction, indicating that the oxidation state of Ru is well maintained. Ru 3p XPS analysis exhibited slight shift to higher binding energy (~0.4 eV), without any evidence of metallic Ru formation, while preserving the relative difference between catalysts. In addition, Ce 3d XPS and Raman analysis confirmed that the ceria structure and the Ru-$CeO_2$ interaction remain largely

unchanged after reaction. **Fig. R8** and **Table R1** were added in revised manuscript as **Supplementary Fig. 28** and **Supplementary Table 5**.

Taken together, the absence of detectable Ru clusters in high-resolution HAADF-STEM, the preferential interaction of Ru on $CeO_2$ domains confirmed by line-EDS analysis, the negligible Ru-Ru coordination revealed by EXAFS, and the consistent structural and electronic features observed after long-term high-pressure reaction collectively support that atomically dispersed Ru species on $CeO_2$ domains are formed and remain stable under ammonia decomposition conditions. While direct in-situ observation remains challenging under these reaction environments, the strong agreement between high-resolution microscopy, spectroscopy, and post-reaction analyses provides consistent evidence for the stability of the atomically dispersed Ru species. Accordingly, these results were added into revised manuscript as shown below.

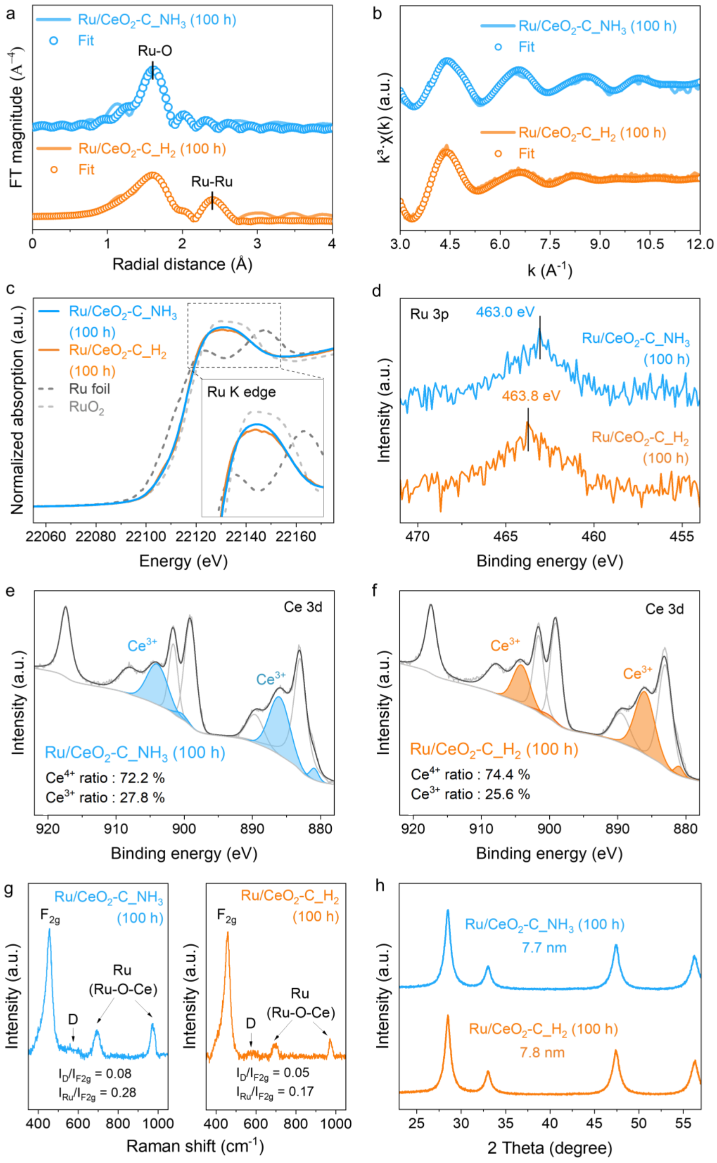


**Fig. R8** EXAFS fitting results of Ru K edge for $Ru/CeO_2$-C_$NH_3$ and $Ru/CeO_2$-C_$H_2$ after durability test in (a) R space, and (b) K space. (c) XANES, (d) Ru 3p, (e, f) Ce 3d, (g) Raman, and (h) XRD results of $Ru/CeO_2$-C_$NH_3$ and $Ru/CeO_2$-C_$H_2$ after high-pressure long term durability test.

**Table R1** Best-fit values for the EXAFS results of Ru/$CeO_2$-C_$NH_3$ and Ru/$CeO_2$-C_$H_2$ after high-pressure long term durability test.

| Sample | Path | Coordination number | Debye-Waller Factor [$\sigma^2 \cdot 10^{-3}$ / Å$^2$] | R [Å] | R-factor |
|---|---|---|---|---|---|
| Ru/$CeO_2$-C_$NH_3$ (100 h) | Ru-O | 3.9 ± 0.6 | 5.6 ± 1.9 | 2.0 ± 0.1 | 0.028 |
| | Ru-Ru | 0.2 ± 0.2 | 3.0* | 2.6 ± 0.1 | |
| Ru/$CeO_2$-C_$H_2$ (100 h) | Ru-O | 3.4 ± 0.4 | 6.5 ± 1.6 | 2.0 ± 0.1 | 0.024 |
| | Ru-Ru | 1.4 ± 0.5 | 6.8 ± 2.0 | 2.6 ± 0.1 | |

* This factor was fixed during the EXAFS fitting

**[Revised manuscript, page 7]**

The spatial distribution and local structure of ruthenium were analyzed using ultrahigh-resolution double Cs-corrected TEM (UHR-TEM) with multichannel energy-dispersive X-ray spectroscopy (multi-EDS). The ruthenium species in Ru/$CeO_2$-C_$N_2$ were randomly distributed over both the $CeO_2$ and carbon domains. They existed either in an atomically dispersed form or as nanoparticles (Fig. 1a and Supplementary Fig. 6). After the $NH_3$ treatment, most of the ruthenium located on the carbon disappeared, and ruthenium selectively migrated toward the $CeO_2$ domains. Only a trace amount of Ru was still detected on the carbon regions together with Ce signals, although the corresponding intensities were significantly lower than those observed on ceria domains (Fig. 1b and Supplementary Fig. 7). In contrast, the $H_2$ treatment formed randomly located Ru nanoparticles on both carbon and $CeO_2$, with mixed sizes (Fig. 1c and Supplementary Fig. 8). Supplementary Fig. 9 shows additional TEM images. To further examine the structure of Ru species on $CeO_2$, UHR-TEM images were taken at higher magnification (Supplementary Fig. 10). The $CeO_2$ lattice was clearly resolved, and no Ru nanoparticles or sub-nanometer clusters were observed. Instead, some isolated bright features were detected along with the ceria domains, consistent with atomically dispersed Ru species. These results suggest that $NH_3$ treatment enables selective migration with atomic dispersion of ruthenium onto $CeO_2$, whereas $H_2$ treatment leads to aggregation into small clusters or nanoparticles at random locations. The deposition of ruthenium on $CeO_2$ and carbon and treatment with $NH_3$ caused it to exist as nanoparticles (Supplementary Fig. 11).

**[Revised manuscript, page 19]**

The catalysts were investigated after long term reaction at high pressure to confirm structure stability. The EXAFS analysis of the Ru/$CeO_2$-C_$NH_3$ after durability test showed that the Ru-O coordination remains high (3.9), while the Ru-Ru coordination remains negligible (0.2), indicating that no significant aggregation of Ru species occurred during reaction. In contrast,

the $Ru/CeO_2$-C_$H_2$ after durability test exhibited a substantially higher Ru-Ru coordination (1.4), consistent with the presence of Ru nanoparticles. These results demonstrated that the atomic Ru structure formed after $NH_3$ treatment was preserved even under prolonged high-pressure ammonia decomposition (Supplementary Fig. 28a, b, and Supplementary Table 5). Furthermore, Ru K-edge XANES spectra showed negligible changes before and after reaction, indicating that the oxidation state of Ru was well maintained (Supplementary Fig. 28c). Ru 3p XPS analysis exhibited slight shift to higher binding energy (~0.4 eV), without any evidence of metallic Ru formation, while preserving the relative difference between catalysts (Supplementary Fig. 28d). In addition, Ce 3d XPS and Raman analysis confirmed that the ceria structure and the Ru-$CeO_2$ interaction remain largely unchanged after reaction (Supplementary Fig. 28e-g). XRD results for the $Ru/CeO_2$-C-based catalysts after 100 h of reaction showed that the $CeO_2$ domains did not aggregate or sinter during the operation (Supplementary Fig. 28h). Finally, the UHR-TEM and multi-EDS results confirmed that the ruthenium in $Ru/CeO_2$-C_$NH_3$ remained atomically dispersed on $CeO_2$ domains even after the long-term high-pressure reaction (Supplementary Fig. 29a). In contrast, the ruthenium in $Ru/CeO_2$-C_$H_2$ still existed as nanoparticles randomly distributed across the support without preferential interaction with $CeO_2$ (Supplementary Fig. 29b).

*5. Why do Ru particles still exist on "Ru/CeC_$H_2$" after 100 hours of catalyzing the ammonia decomposition reaction, and why have Ru species not completely migrated to $CeO_2$? This seems inconsistent with the main argument of the manuscript. If "Ru/CeC_$H_2$" is treated with air at low temperature (e.g., 300 °C) and then tested for ammonia decomposition, how would its activity and structure change?*

**Answer)**

We thank the reviewer for this insightful question. The reviewer correctly points out a potential inconsistency between the proposed ammonia-driven migration mechanism and the observation that Ru nanoparticles remain on the $Ru/CeO_2$-C_$H_2$ catalyst even after prolonged ammonia decomposition. To clarify this point, we performed additional control experiments and carefully reconsidered the mechanism of Ru redistribution.

Our results in manuscript indicate that ammonia-driven restructuring is not a continuous process occurring throughout the entire reaction, but rather a kinetically limited restructuring event that occurs during the early state of catalyst evolution when Ru species remain sufficiently mobile. Specifically, during $NH_3$ pretreatment, the generation of active nitrogen species promotes the mobility of Ru species and enables their preferential migration toward $CeO_2$ domains, where they form stabilized Ru-ceria interfacial structures.

In contrast, when the catalyst is initially treated in $H_2$, metallic Ru nanoparticles are formed prior to ammonia exposure. These particles exhibited strong Ru-Ru metallic bonding and can additionally become anchored at defect sites on the carbon support, as evidenced by the EXAFS results (**Fig. 2a**), together with generation of $CH_4$ observed during the reduction process (**Fig. 5b,c**). As a result, the detachment of Ru atoms from these stabilized nanoparticles becomes kinetically hindered, preventing complete redistribution toward $CeO_2$ during subsequent ammonia decomposition.

To experimentally examine whether the Ru/$CeO_2$-C_$H_2$ structure could be restructured after oxidation, as suggested by the reviewer, we performed additional oxidation treatments. First, low-temperature air oxidation at 300 °C followed by ammonia treatment at 500 °C was done on Ru/$CeO_2$-C_$H_2$, but did not lead to any measurable change in catalytic activity. Oxidation temperature was set to 300 °C from suggestion of the Reviewer, but also from the temperature programmed oxidation (TPO) result of $CeO_2$-C support, indicating possible carbon oxidation over 300 °C (**Fig. R9**).

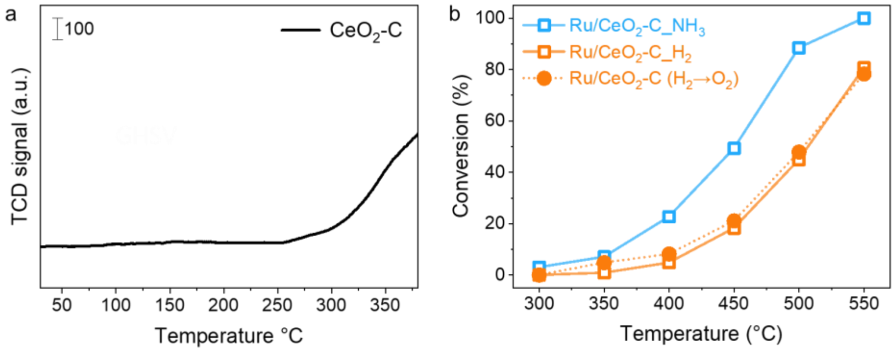


**Fig. R9** (a) Temperature-programmed oxidation (TPO) result of $CeO_2$-C. Oxidation was performed with 5 % $O_2$/He. (b) Catalytic performance comparison of Ru/$CeO_2$-C catalysts under different pretreatment conditions. The activity of Ru/$CeO_2$-C_$NH_3$, Ru/$CeO_2$-C_$H_2$, and Ru/$CeO_2$-C ($H_2 \rightarrow O_2$) catalysts are shown as a function of reaction temperature. The Ru/$CeO_2$-C ($H_2 \rightarrow O_2$) sample corresponds to Ru/$CeO_2$-C_$H_2$ that was mildly oxidized in air at 300 °C, then underwent ammonia treatment prior to testing. $NH_3$ decomposition was conducted using 100% $NH_3$ with a WHSV of 30,000 mL/$g_{cat}$·h.

Secondly, ammonia treatment was done at higher temperature on Ru/$CeO_2$-C_$H_2$. Ammonia treatment at 600 °C resulted in a modest increase in catalytic activity, accompanied by partial redistribution of Ru species toward $CeO_2$ domains as observed by UHR-TEM (**Fig. R10**). This observation suggests that some degree of restructuring remains possible when sufficiently high driving force of Ru species is induced. However, ammonia treatment at 700 °C led to decreased catalytic activity.

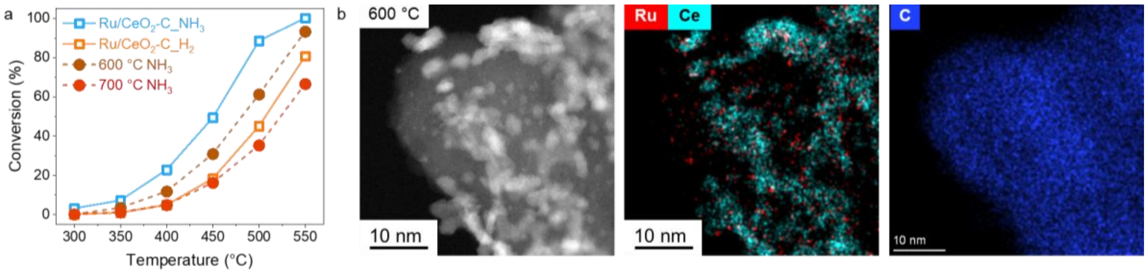


**Fig. R10** (a) Catalytic performance comparison of Ru/$CeO_2$-C_$H_2$ catalysts under different pretreatment conditions. The activity of Ru/$CeO_2$-C_$NH_3$, Ru/$CeO_2$-C_$H_2$, and Ru/$CeO_2$-C_$H_2$ catalysts after 600 °C and 700 °C of ammonia treatments are shown. $NH_3$ decomposition was conducted using 100% $NH_3$ with a WHSV of 30,000 mL/$g_{cat}$·h. (b) UHR-TEM and corresponding EDS mapping results of Ru/$CeO_2$-C_$H_2$ after 600 °C ammonia treatment.

These results collectively suggest that ammonia-driven migration occurs within a limited kinetic window before Ru nanoparticles become fully stabilized. Once strong Ru-Ru bonding and anchoring interactions are established, further redistribution becomes increasingly difficult even under ammonia decomposition conditions, highlighting the critical role of the initial chemical environment in determining the final catalyst structure. At the same time, the partial redistribution observed after the 600 °C ammonia treatment indicated that limited restructuring can still occur under sufficient energetic conditions, although the extent of redistribution remains restricted once Ru nanoparticles are strongly stabilized.

Therefore, the persistence of Ru nanoparticles in Ru/$CeO_2$-C_$H_2$ after long-term reaction does not contradict the ammonia-driven migration mechanism proposed in this work. Instead, it highlights the importance of the early-stage reaction environment in directing catalyst evolution, where initial exposure to ammonia enables controlled Ru migration and the formation of stable Ru-ceria interfacial structures. The corresponding discussion have been incorporated into the revised manuscript.

**[Revised manuscript, page 20]**

Finally, the UHR-TEM and multi-EDS results confirmed that the ruthenium in Ru/$CeO_2$-C_$NH_3$ remained atomically dispersed on $CeO_2$ domains even after the long-term high-pressure reaction (Supplementary Fig. 29a). In contrast, the ruthenium in Ru/$CeO_2$-C_$H_2$ still existed as nanoparticles randomly distributed across the support without preferential interaction with $CeO_2$ (Supplementary Fig. 29b). This structural conservation suggests that ammonia-driven restructuring occurs primarily before the formation of stabilized Ru nanoparticles. Once strong Ru-Ru bonding and anchoring interactions are established, large-scale redistribution became kinetically hindered. Consequently, initial $NH_3$ treatment directed the formation of energetically stabilized and structurally robust Ru-$CeO_2$ interfaces, which preserve atomic dispersion and catalytic performance under high-pressure $NH_3$ decomposition.

*6. Will "Ru/CeC_NH3" and "Ru/CeC_H2" yield the same catalyst structure when catalyzing the ammonia decomposition reaction? Will the second or third round of activity test results be the same?*

**Answer)**

We thank the reviewer for the question regarding the structural stability and reproducibility of the catalysts. First, we note that the structural evolution of both catalysts after prolonged reaction has already been carefully examined. The catalysts subjected to 100 hours of high-pressure ammonia decomposition were characterized using UHR-TEM, EXAFS, XPS, Raman spectroscopy, and XRD analysis. These results consistently indicated that the catalyst structures after reaction remain essentially identical to those before reaction. Specifically, Ru/$CeO_2$-C_$NH_3$ maintained atomically dispersed Ru species on $CeO_2$ domains, while Ru/$CeO_2$-C_$H_2$ retained Ru nanoparticles randomly distributed on the support without preferential interaction with $CeO_2$. These observations confirmed that the catalyst structures remain stable during long-term high-pressure operation.

The reviewer also raised a valid point regarding the reproducibility of catalytic performance in repeated reaction cycles. To address this, we additionally performed high-pressure cycling experiments. After completing one reaction cycle, the reactor was cooled under a continuous $NH_3$ flow and subsequently reheated for the next cycle. The catalytic performance in the following cycles remained unchanged, indicating that the catalysts maintain their activity during repeated reaction-cooling cycles (**Fig. R11**).

These results further support that both Ru/$CeO_2$-C_$NH_3$ and Ru/$CeO_2$-C_$H_2$ preserve their structural characteristics and catalytic performance under repeated ammonia decomposition conditions. The additional cycling results have been included in the revised manuscript as **Supplementary Fig. 27**.

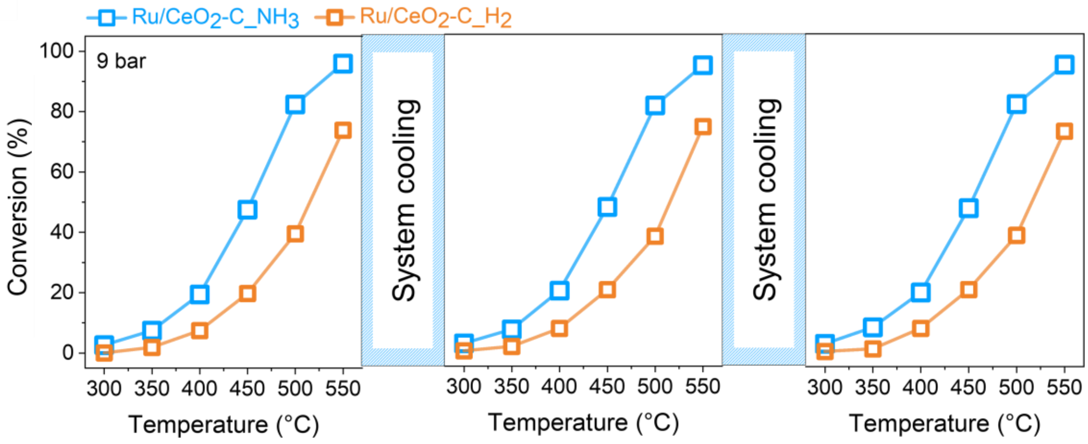


**Fig. R11** Catalytic performance of Ru/$CeO_2$-C_$NH_3$ and Ru/$CeO_2$-C_$H_2$ for high-pressure ammonia decomposition reaction for three repeated cycles. $NH_3$ decomposition was conducted using 100% $NH_3$ with a WHSV of 30,000 mL/$g_{cat}$·h.

**[Revised manuscript, page 19]**

The structural stability of the catalysts was assessed through a long-term durability test for 100 h at 1 and 9 bar. Both Ru/$CeO_2$-C_$NH_3$ and Ru/$CeO_2$-C_$H_2$ maintained their initial catalytic activities throughout the reactions without noticeable deactivation, indicating their high operational durability under these reaction conditions (Fig. 4c). In addition, the catalytic performance was maintained over three consecutive reaction cycles under high-pressure conditions (Supplementary Fig. 27).

*7. Figure S3 needs to provide quantitative data on the proportions of the various species.*

**Answer)**

We thank the reviewer for this helpful suggestion. Quantitative information on the proportions of the different species has now been added to **Supplementary Fig. 3.** In addition, the relative proportions derived from the XPS peak deconvolution have been consistently included for all relevant XPS spectra throughout the manuscript and the Supplementary Information.

*8. For the "CeC" supports calcined at different temperatures, the authors concluded via $H_2$-TPR that the sample calcined at 550 °C is optimal. However, the size of $CeO_2$ in the sample calcined at 300 °C is smaller, and I am curious about the activity of the catalyst prepared by loading Ru on the "CeC-300 °C".*

**Answer)**

We thank the reviewer for this comment. Indeed, the $CeO_2$-C support calcined at 300 °C exhibited smaller $CeO_2$ domains and a higher concentration of surface defects, as evidenced by the XPS O 1s and Ce 3d spectra (**Supplementary Fig. 3**). However, for ammonia decomposition, we considered the dynamic redox properties of the support to be more critical than particle size or intrinsic defect concentration. Based on the $H_2$-TPR results, the $CeO_2$-C support calcined at 550 °C showed the most favorable reducibility, which led us to select this sample as the optimal support (**Supplementary Fig. 4**).

To address the reviewer's suggestion, we additionally prepared Ru catalysts supported on $CeO_2$-C calcined at different temperatures (300 °C and 700 °C). After Ru loading, the catalysts were pretreated in $NH_3$ at 500 °C for 1 h and evaluated for ammonia decomposition under identical conditions (**Fig. R12**).

The catalyst prepared on the support calcined at 550 °C exhibited the highest catalytic activity. The catalyst based on the 300 °C support showed lower activity, while the catalyst prepared on the support calcined at 700 °C exhibited the lowest activity. These results confirm that the $CeO_2$-C support calcined at 550 °C provides the most favorable balance of structural and redox properties for ammonia decomposition, consistent with our original selection based on the $H_2$-TPR analysis.

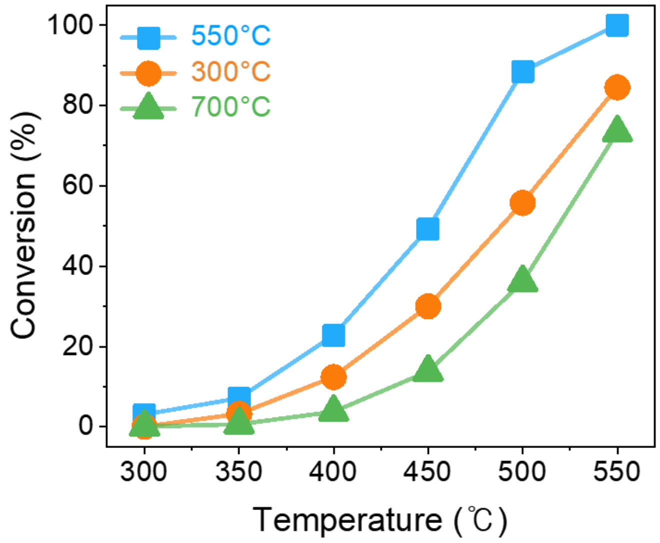


**Fig. R12.** Catalytic performance of Ru catalysts on $CeO_2$-C support with different calcination temperature. All catalysts were pretreated with ammonia at 500 °C for 1 hour, and $NH_3$ decomposition was conducted using 100% $NH_3$ with a WHSV of 30,000 mL/$g_{cat}$·h.

*9. “Ru/CeC_$H_2$” has already been reduced at 500 °C, so why does it still show reduction peaks at low temperatures in the result of $H_2$-TPR? Similarly, the reduction peaks of “Ru/CeC_$NH_3$” also needs to be confirmed.*

**Answer)**

We thank the reviewer for this comment. We agree that the presence of low-temperature reduction peaks in $H_2$-TPR may appear inconsistent with the prior reduction treatment at 500 °C. To clarify this point, we performed additional cryo-temperature programmed oxidation (cryo-TPO) experiments to directly examine the oxidation behavior of the catalysts.

In the cryo-TPO measurements, the samples were first mildly reduced at 200 °C under 5 % $H_2$/Ar, followed by cooling to -95 °C under He. Subsequently, the gas was switched to 5 % $O_2$/He, and after stabilization, the temperature increased to 400 °C while monitoring oxygen consumption. As shown in **Fig. R13**, both Ru/$CeO_2$-C_$NH_3$ and Ru/$CeO_2$-C_$H_2$ exhibited clear oxygen uptake starting already at -95 °C, indicating that the Ru-containing surface can be readily oxidized even at very low temperatures. This observation provides a direct explanation for the low-temperature reduction peaks observed in $H_2$-TPR. Although the catalysts were initially treated under reducing conditions during synthesis, the surface of Ru species can be readily re-oxidized upon exposure to air prior to the TPR measurement. Therefore, the low-temperature $H_2$-TPR features are attributed to the reduction of these re-oxidized surface species, rather than incomplete reduction during the initial pretreatment.

Importantly, the cryo-TPO results also revealed a clear difference between the two catalysts. Ru/$CeO_2$-C_$H_2$ showed a relatively small and sharp low-temperature oxidation peak, whereas Ru/$CeO_2$-C_$NH_3$ exhibited a larger oxygen uptake with an additional shoulder feature at low temperature. Furthermore, both catalysts displayed a broader oxidation feature around ~200 °C, which was more pronounced for Ru/$CeO_2$-C_$NH_3$. At higher temperatures above 300 °C, a dominant peak appeared for both samples, which was attributed to oxidation of the carbon support. These results suggested that Ru/$CeO_2$-C_$NH_3$ contains a greater amount of oxygen-associated Ru species compared to Ru/$CeO_2$-C_$H_2$. This behavior was consistent with the formation of highly dispersed Ru species strongly interacting with the $CeO_2$ support, leading to a more oxygen-rich interfacial environment. In contrast, Ru/$CeO_2$-C_$H_2$ exhibited comparatively lower oxygen uptake, indicating less extensive interaction between Ru and the oxide support. Overall, the combined $H_2$-TPR and cryo-TPO results consistently indicated that Ru/$CeO_2$-C_$NH_3$ possesses a higher density of redox-active oxygen species. Accordingly, **Fig. R13** was added as a **Supplementary Fig. 13**, and we have revised the manuscript to include it.

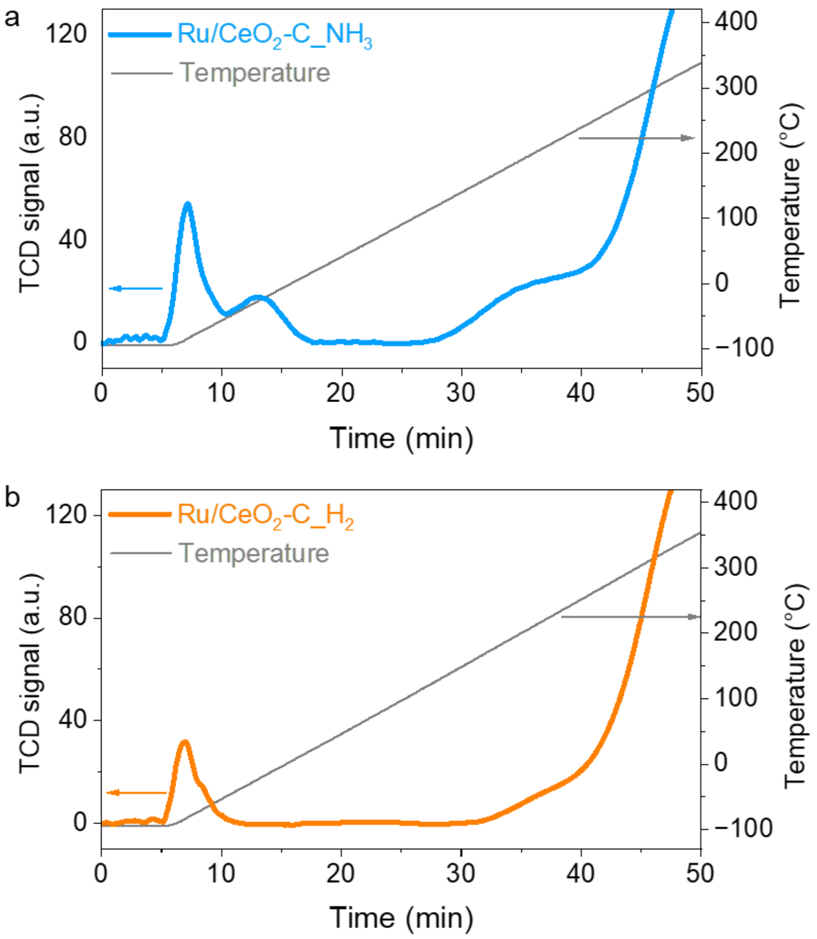


**Fig. R13** Cryo-TPO results of (a) Ru/$CeO_2$-C_$NH_3$ and (b) Ru/$CeO_2$-C_$H_2$.

**[Revised manuscript, page 9]**

In addition to the XAS results, $H_2$-TPR analyses were performed to gain deeper insight into the surface reducibility of the catalysts and their metal-support interactions (Fig. 2c). Ru/$CeO_2$ exhibited a small reduction peak at 123 °C, while Ru/C exhibited no reduction peak. Ru/$CeO_2$-C_$NH_3$ exhibited a single broad reduction peak at 157 °C, whereas Ru/$CeO_2$-C_$H_2$ exhibited two distinct peaks at 89 and 112 °C. The higher reduction temperature of Ru/$CeO_2$-C_$NH_3$ indicates that Ru forms strong interactions with $CeO_2$, likely as Ru-O-Ce species. In contrast, the peaks at lower temperatures for Ru/$CeO_2$-C_$H_2$ suggest the presence of weakly bound ruthenium nanoparticles or clusters on $CeO_2$. Notably, Ru/$CeO_2$-C_$NH_3$ also exhibited a larger reduction peak area, indicating a greater amount of reducible oxygen species. This feature suggests the presence of a higher density of redox-active interfaces in the catalyst, which are associated with surface defect concentration. Such an increase in reducible oxygen is expected to enhance redox cycling at the Ru-$CeO_2$ interface, potentially facilitating hydrogen adsorption-desorption dynamics and mitigating hydrogen accumulation on Ru sites. To further probe the oxygen-related surface properties, cryo-TPO were conducted (Supplementary Fig. 13). Both catalysts exhibited oxygen uptake starting from -95 °C, indicating that the Ru surface can be readily oxidized even at very low temperatures. Notably, Ru/$CeO_2$-C_$NH_3$ showed a larger oxygen uptake with an additional low-temperature shoulder feature compared to

$Ru/CeO_2$-C_$H_2$, suggesting a higher density of oxygen-taking Ru species. This result is consistent with the formation of strongly interacting and highly dispersed Ru species on $CeO_2$, leading to a more oxygen-rich interfacial environment. Overall, the combined $H_2$-TPR and cryo-TPO results support that $Ru/CeO_2$-C_$NH_3$ possesses a higher density of redox-active interfacial oxygen species.

**[Revised Supplementary Information, Characterization]**

Temperature-programmed reduction using hydrogen ($H_2$-TPR) was conducted with a BELCAT M instrument (MicrotracBEL) equipped with a thermal conductivity detector (TCD). Prior to measurement, the catalysts were pretreated under argon at 150 °C for 1 h and then cooled to room temperature. $CO_2$ temperature-programmed desorption ($CO_2$-TPD) was carried out on a BELCAT II (MicrotracBEL) with a mass spectrometer (BELMASS; MicrotracBEL) as a detector. Catalysts were pretreated under helium at 200 °C for 1 h and then cooled to room temperature. $CO_2$ was purged for 30 min, and He was injected to eliminate gaseous $CO_2$. Temperature was ramped up with 10 °C, observing $CO_2$ emission with mass spectrometer. Cryo-TPO and $H_2$-TPD were conducted with AutoChem III (Micromeritics) equipped with a thermal conductivity detector (TCD). In case of cryo-TPO, the samples were first mildly reduced at 200 °C under 5% $H_2$/Ar, followed by cooling to -95 °C under He. Subsequently, the gas was switched to 5% $O_2$/He, and after stabilization, the temperature was increased to 400 °C while monitoring oxygen consumption.

*10. The peak of D in Fig. 2d is very small. Generally, defect-rich $CeO_2$ exhibits a significant D peak.*

**Answer)**

We thank the reviewer for this comment. We have also carefully examined the defect-related Raman features of the $CeO_2$-C support. In our system, however, the $CeO_2$ domains are dispersed on a carbon matrix, which makes it challenging to selectively probe $CeO_2$-rich regions during Raman measurements. Also, as the Raman spectra had strong carbon signals, the defect-related features of the $CeO_2$ remained relatively weak.

To further investigate this point, we measured Raman spectra of the $CeO_2$-C supports thermally treated at different temperatures (300, 550, and 700 °C). In all cases, the intensity of the defect-related band showed only minor differences among the samples (**Fig. R14**)

In addition, in-situ Raman measurements were performed during thermal reduction under 4 % $H_2$/Ar at temperature ranging from 100 to 500 °C. Results from these measurements also did not reveal significant changes in the defect-related peak intensity (**Fig. R15**). These results suggest that in $CeO_2$-C system, Raman spectroscopy has limited sensitivity for quantitatively comparing defect concentrations due to the strong contribution of the carbon matrix and the heterogeneous distribution of $CeO_2$ domains. Therefore, we primarily relied on XPS analysis (Ce 3 and O 1s spectra) to evaluate the relative defect concentration of the supports, which provided clearer and more consistent information on the defect structure. In the manuscript, Raman analysis is mainly used to identify changes in the Ru-$CeO_2$ interaction, where the spectral features are clearly distinguishable in that case.

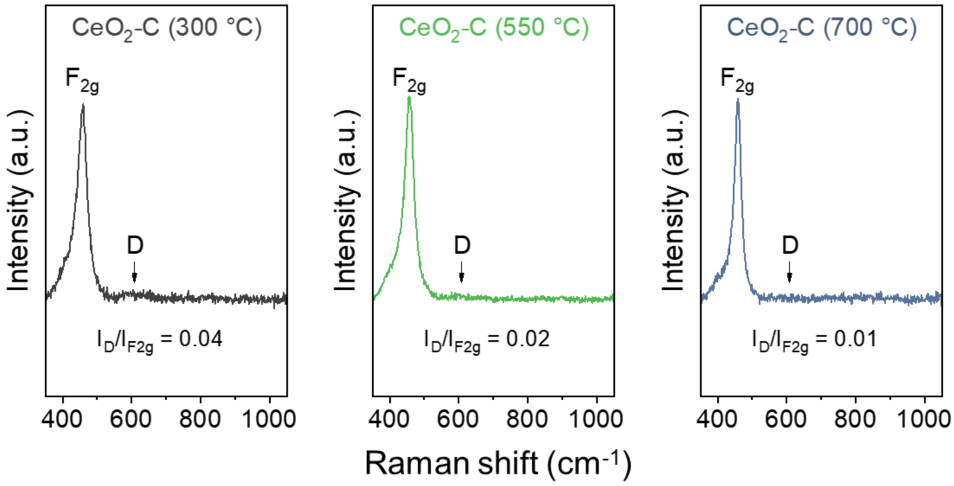


**Fig. R14** Raman spectra results of $CeO_2$-C calcined at 300, 550, and 700 °C under $N_2$.

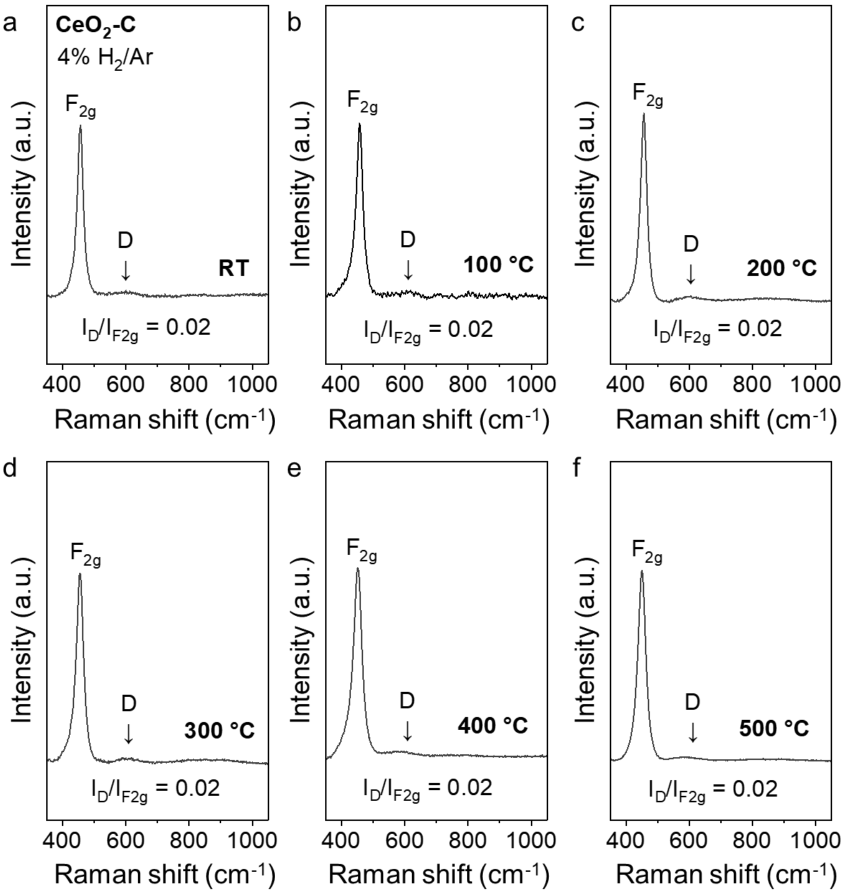


**Fig. R15** In-situ Raman spectra of $CeO_2$-C support treated at 550 °C. Experiments were conducted under 4 % $H_2$/Ar gas conditions, with raising temperature from room temperature to 500 °C.

*11. In Fig. 3b, the pure support "CeC" and C achieve 80-90% conversion at 700 °C. I believe this data is highly problematic. Please provide the activity results of a blank reactor test.*

**Answer)**

We thank the reviewer for this important comment. We acknowledge that ammonia decomposition is an endothermic reaction, so it is thermally promoted at high temperatures. To address this point, blank reactor tests were performed using the same quartz reactor (I.D. ½ inch) up to 700 °C (**Fig. R16**). The blank test showed that $NH_3$ decomposition is negligible up to approximately 500 °C, where the observed conversion is comparable to that of the $CeO_2$-C and C supports. At higher temperatures (e.g., 700 °C), a significant contribution from non-catalytic thermal decomposition is observed in the blank reactor (~ 65 %). Therefore, we agree that the apparent $NH_3$ conversion observed for $CeO_2$-C and carbon at 700 °C cannot be attributed solely to the intrinsic catalytic activity of the supports.

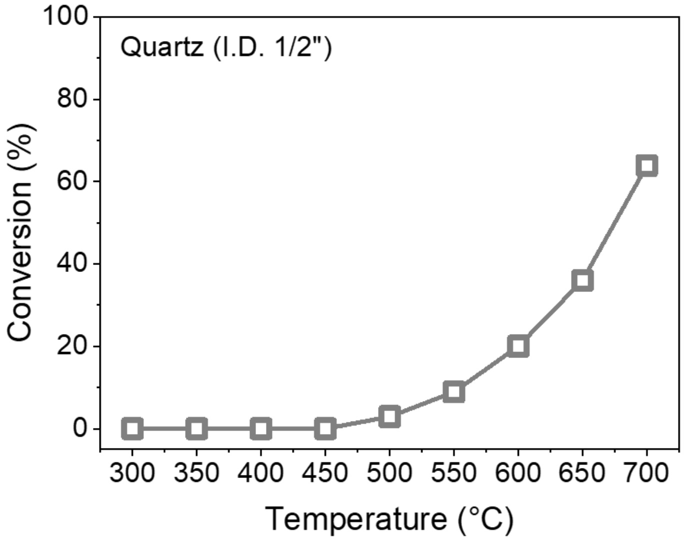


**Fig. R16** $NH_3$ conversion results for blank test using quartz reactor with inner diameter of ½ inch. Ammonia flow was 40 mL/min, which corresponds to WHSV of 30,000 mL/$g_{cat}$·h with catalysts.

At the same time, we would like to clarify the purpose of the experiment shown in **Fig. 3b**. The intention was not to directly compare the intrinsic ammonia decomposition activity of the supports, but rather to generate comparable $NH_x$ species under controlled $NH_3$ treatment conditions and examine their interaction with different materials. In this context, although ammonia decomposition at elevated temperature is largely dominated by thermal cracking in the quartz reactor, the quartz itself does not act as a sink for the generated $NH_x$ species. Instead, these nitrogen-containing intermediates are more likely to interact with and be stabilized on the introduced support materials ($CeO_2$-C and carbon). Therefore, the experimental approach remains valid for probing the interaction and stabilization of $NH_x$ species on different supports.

Accordingly, the overall interpretation of **Fig. 3b**, focusing on the stabilization of $NH_x$ species on the supports, is not significantly affected. To avoid potential misinterpretation, we have added the blank data to the **Supplementary Fig. 17** and revised the manuscript to clarify that

the support-only conversion at high temperature includes a significant contribution from thermal decomposition and should not be interpreted as intrinsic catalytic activity.

**[Revised Supplementary Information, Ammonia decomposition]**

Ammonia decomposition for hydrogen production was carried out in a fixed-bed quartz reactor with ½-inch diameter under a flow of pure $NH_3$. For high-pressure experiments, a ½-inch SUS reactor was used.

**[Revised manuscript, page 12]**

As ruthenium migration is likely driven by its interaction with nitrogen-based intermediates, it was hypothesized that the stabilization sites of these nitrogen species would correlate with the anchoring sites of the mobilized ruthenium. To test this hypothesis, various ruthenium catalysts and their corresponding supports were treated under similar conditions with comparable $NH_3$ decomposition rates (Fig. 3b). Blank test result using the quartz reactor is shown in Supplementary Fig. 17.

*12. After treating "CeC" with $NH_3$ and then loading Ru, will Ru still deposit on the C?*

**Answer)**

We thank the reviewer for this insightful question. To address this point, we prepared a control sample in which the $CeO_2$-C support was first treated with $NH_3$, followed by Ru loading (Ru/$NH_3$-$CeO_2$-C). In this case, the support was pretreated at 650 °C to generate a comparable level of $NH_x$-related surface species, consistent with the conditions used in the original manuscript (**Fig. 3b**).

The resulting catalyst exhibited catalytic performance comparable to that of Ru/$CeO_2$-C_$N_2$, indicating that $NH_3$ pretreatment of the support alone does not lead to a significant enhancement in activity (**Fig. R17**). This observation suggests that Ru species are not selectively anchored exclusively on $CeO_2$ domains during the post-loading step. In addition, given the similar catalytic behavior of Ru/$NH_3$-$CeO_2$-C with Ru/$CeO_2$-C_$N_2$, it is reasonable to infer that the overall surface configuration of this sample is also similar. From this perspective, it is likely that a fraction of Ru species remains distributed on the carbon phase, although this conclusion is drawn indirectly based on activity-structure correlation.

These results further imply that the initial nitrogen incorporation into the support is not sufficient to induce selective anchoring of Ru species. Instead, $NH_3$ treatment on the Ru catalysts appears to play a more critical role. Taken together, these findings indicate that the preferential localization of Ru on $CeO_2$ domains observed in this study is not simply achieved by pre-treating the support with $NH_3$, but is driven by the reaction environment during ammonia decomposition. This supports our conclusion that reaction-induced restructuring of Ru species plays a key role in forming the active Ru-$CeO_2$ interfacial configuration.

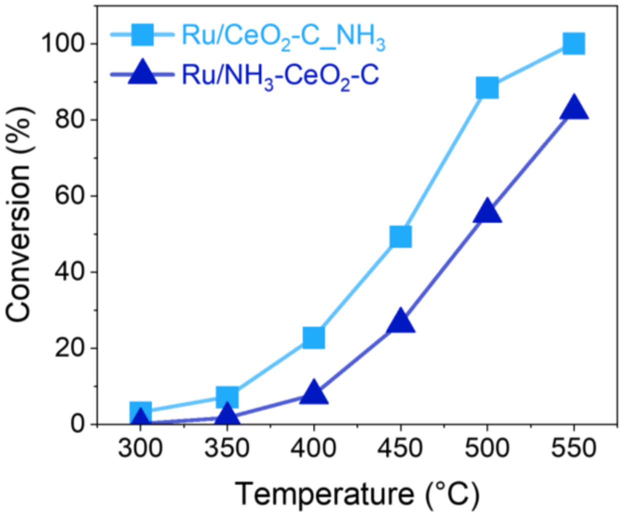


**Fig. R17** Catalytic performance of $Ru/CeO_2$-C_$NH_3$ and $Ru/NH_3$-$CeO_2$-C. $Ru/NH_3$-$CeO_2$-C indicates a catalyst synthesized by depositing Ru on ammonia treated $CeO_2$-C. $NH_3$ decomposition was conducted using 100% $NH_3$ with a WHSV of 30,000 mL/$g_{cat}$·h.

Instead, to obtain insight into the structural evolution during ammonia treatment, we performed a time-resolved analysis using UHR-TEM and multi-EDS detectors (**Fig. R18**). In this approach, $Ru/CeO_2$-C_$N_2$ was exposed to $NH_3$ at 500 °C for different times (1, 5, 10, 20, and 40 minutes), followed by rapid quenching and sampling. This method allowed us to capture intermediate structural states during the ammonia-induced restructuring process.

The results revealed a clear temporal evolution of Ru species. At short treatment times (1 and 5 min), Ru species remain widely distributed, with a significant fraction still located on the carbon phase. A notable transition occurred between 5 and 10 min, where the number of Ru particles observed on carbon decreases, accompanied by a substantial reduction in the Ru signal detected from carbon regions in EDS analysis. With further treatment, this trend became more pronounced. After 40 min, Ru signals from EDS mapping were predominantly localized around $CeO_2$ domains. Although weak residual signals of Ru and Ce are occasionally detected in carbon regions, these signals were likely associated with trace amount of Ru, background or projection effects rather than discrete Ru species (**Fig. R2**). Finally, after 1 hour of ammonia treatment, the structure appeared to be stabilized, with most of Ru making interaction with $CeO_2$, as shown in **Fig. 1b** of the original manuscript.

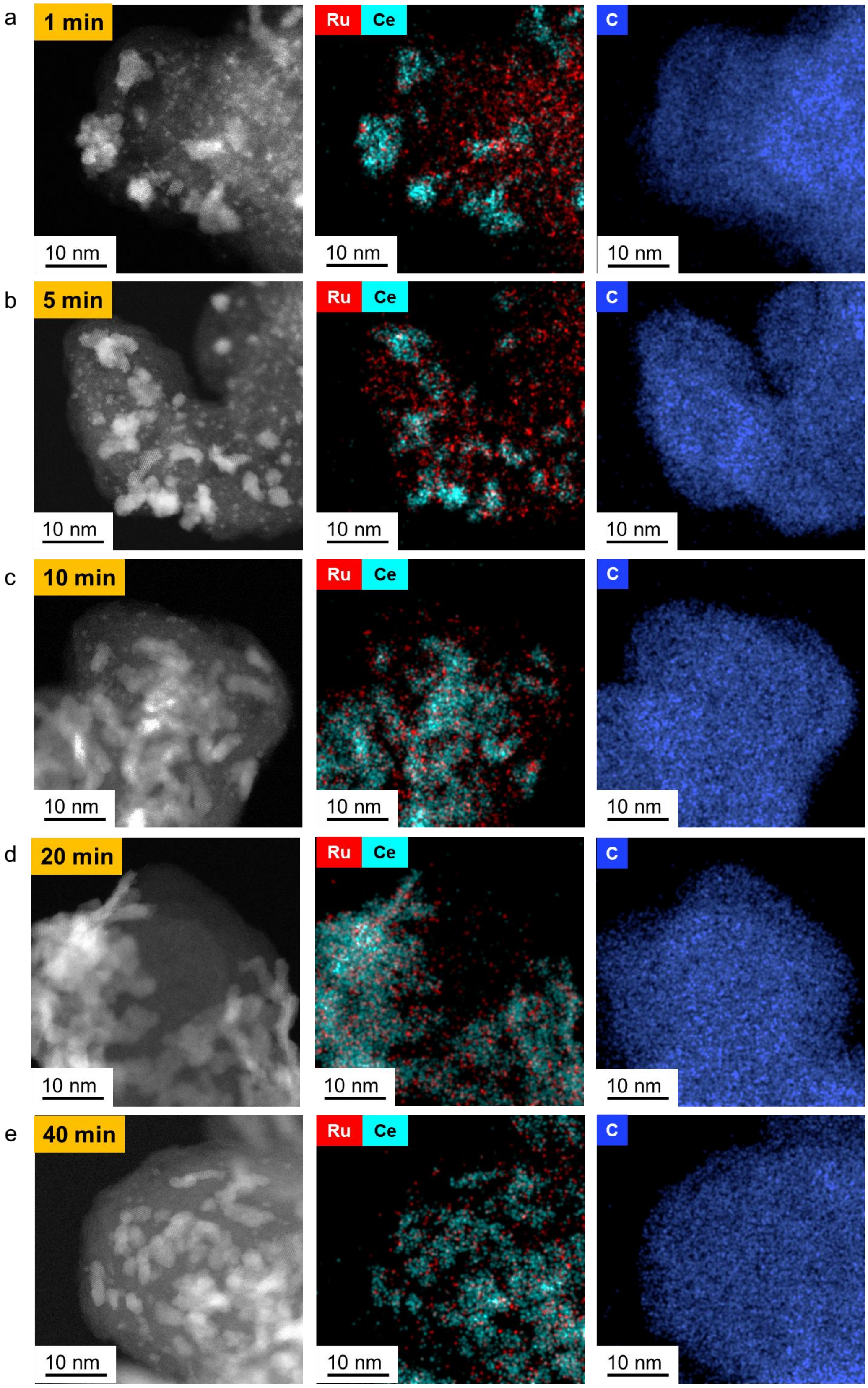


**Fig. R18** Time-resolved UHR-TEM images and corresponding multi-EDS results. Images were taken after $NH_3$ treatment on Ru/$CeO_2$-C_$N_2$ for 1 min, 5 min, 10 min, 20 min, and 40 min at 500 °C

*13. The $E_a$ in this manuscript was so low. The $NH_3$ conversion was generally controlled below 15% during the test process. In addition, whether the test process was affected by diffusion effect?*

**Answer)**

We thank the reviewer for this comment, regarding the possible influence of diffusion limitations. Following the reviewer's suggestion, we performed additional kinetic measurements under conditions designed to further minimize mass-transfer effects. Specifically, the space velocity was increased to 120,000 mL/$g_{cat}$·h, and the activation energies were re-evaluated under this higher space-velocity condition. Under these conditions, the $NH_3$ conversion was maintained below approximately 17 %, ensuring that the measurements were performed within a low-conversion regime while further suppressing possible diffusion limitations.

The catalytic activities at WHSV of 120,000 mL/$g_{cat}$·h are shown in **Fig. R19a**. For Ru/$CeO_2$-C_$NH_3$, the activation energy was determined from measurements at 420, 430, 440, 450, and 460 °C, whereas for Ru/$CeO_2$-C_$H_2$, measurements were performed at 430, 440, 450, 460, and 470 °C. The corresponding Arrhenius plots are presented in **Fig. R19b**. From these measurements, the activation energy of Ru/$CeO_2$-C_$NH_3$ was determined to be 64.0 kJ/mol, while that of Ru/$CeO_2$-C_$H_2$ was 103.9 kJ/mol. These values are higher than those obtained from the original measurements (61.8 and 74.5 kJ/mol, respectively), indicating that the influence of mass-transfer effects is further reduced under the higher space velocity condition.

Importantly, the updated activation energies fall within the range commonly reported for Ru-based ammonia decomposition catalysts. For example, Yamazaki et al. reported an activation energy for 87.6 kJ/mol for Ru/$CeO_2$ catalysts[1], while Fang et al. reported 71.3 kJ/mol for Ru/MgO catalysts[2]. Accordingly, the updated Arrhenius plots replaced the previous data shown in **Supplementary Fig. S26a**.

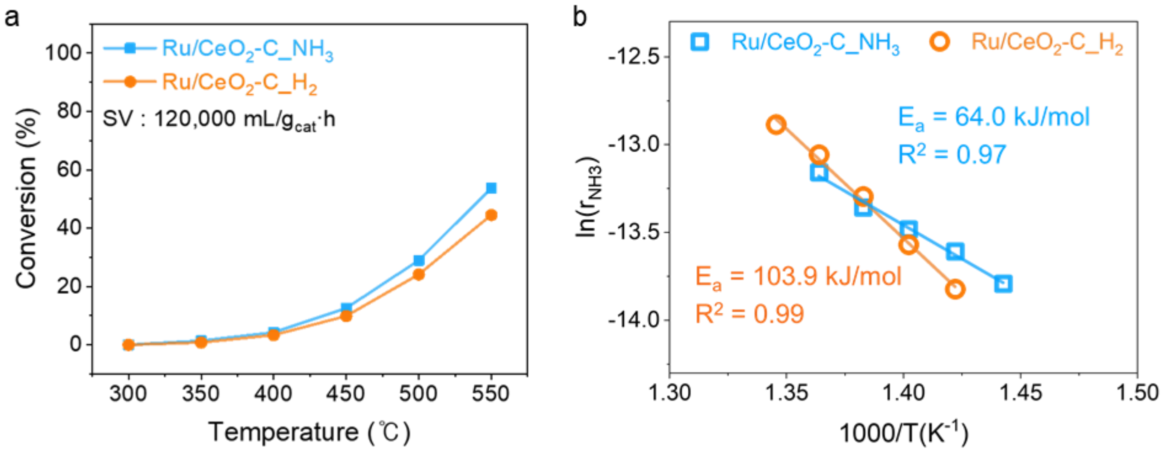


**Fig. R19.** (a) Catalytic activity of Ru/$CeO_2$-C_$NH_3$ and Ru/$CeO_2$-C_$H_2$ for ammonia decomposition in space velocity of 120,000 mL/$g_{cat}$·h. (b) Arrhenius plot of Ru/$CeO_2$-C_$NH_3$ and Ru/$CeO_2$-C_$H_2$ with activation energy values.

**[Revised Supplementary Information, Ammonia decomposition]**

For activation barrier, the weight hourly space velocity (WHSV) was maintained at 120,000 mL/$g_{cat}$·h to ensure sufficient $NH_3$ feed during the reaction. For reaction order experiments with $H_2$ and $NH_3$, the weight hourly space velocity (WHSV) was maintained at 60,000 mL/$g_{cat}$·h. Activation barrier measurement under ambient pressure for Ru/$CeO_2$-C_$NH_3$ was conducted at 420, 430, 440, 450, and 460 °C. At each temperature point, the reaction was allowed to reach equilibrium before the next step. For Ru/$CeO_2$-C_$H_2$, experiment was performed at 430 °C, 440 °C, 450 °C, 460 °C, and 470 °C.

**[References]**


1. Yamazaki, K., Matsumoto, M., Ishikawa, M., & Sato, A., $NH_3$ decomposition over Ru/$CeO_2$-$PrO_x$ catalyst under high space velocity conditions for an on-site $H_2$ fueling station. *Applied Catalysis B: Environmental*, **325**, 122352, (2023).
2. Fang, H., Wu, S., Ayvali, T., Zheng, J., Fellowes, J., Ho, P. L., ... & Tsang, S. C. E. Dispersed surface Ru ensembles on MgO (111) for catalytic ammonia decomposition. *Nature Communications*, **14(1)**, 647, (2023).


*14. In Fig. 5f, the binding energy ranges of C 1s and Ru 3d highly overlap, requiring comprehensive analysis.*

**Answer)**

We thank the reviewer for pointing out the potential overlap between C 1s and Ru 3d binding energy regions in **Fig. 5f**. Indeed, the Ru 3d doublet consists of Ru $3d_{5/2}$ at approximately 279.7-280.0 eV and Ru $3d_{3/2}$ at 284.0-284.5 eV, the latter overlapping with the main C 1s peak typically centered near 284.0-284.8 eV[1, 2]. For this reason, in carbon-supported catalysts the Ru $3d_{3/2}$ component is generally difficult to resolve, and the chemical-state analysis of Ru is commonly based on the Ru $3d_{5/2}$ peak at 279.8 eV, which is less affected by carbon-related contributions.

In the present work, the C 1s spectra were carefully examined down to 279 eV, and no additional features attributable to Ru 3d were observed within the C 1s region (**Fig. R20a**). The spectra were further inspected in the 279-282 eV range under magnified conditions, where no distinct Ru-related contributions could be identified (**Fig. R20b**). In addition, we should note that relative low Ru loading (1.6 wt %) may result in a weak Ru signal compared to the strong carbon signal from the support, which further masks the Ru $3d_{3/2}$ contribution around 284 eV. Therefore, although partial overlap between the C 1s and Ru 3d regions is acknowledged, the experimental results indicate that the C 1s spectra and their fitting are not significantly influenced by Ru-related contributions in this system.

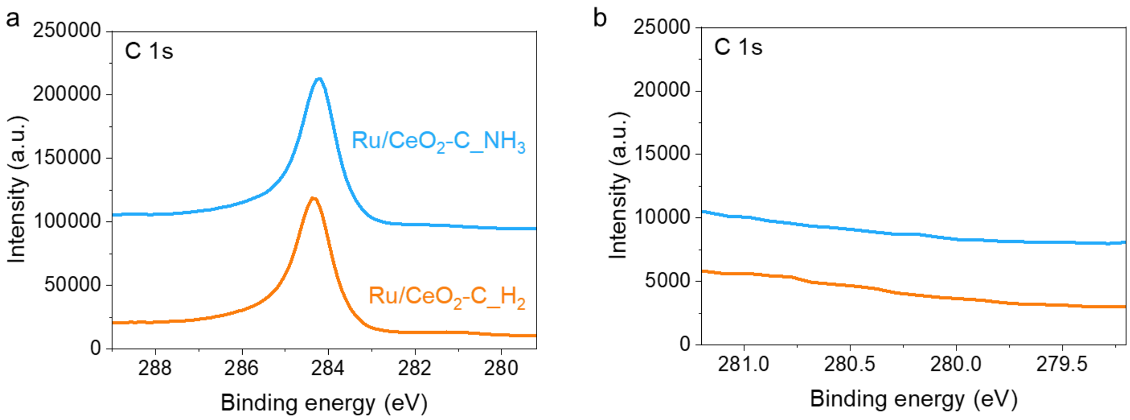


**Fig. R20.** C 1s XPS spectra of Ru/$CeO_2$-C_$NH_3$ and Ru/$CeO_2$-C_$H_2$.

**[References]**


1 Bavand, R., Yelon, A., & Sacher, E., X-ray photoelectron spectroscopic and morphologic studies of Ru nanoparticles deposited onto highly oriented pyrolytic graphite. *Applied Surface Science*, **355**, 279-289, (2015).

2 Li, M., Yan, W., Liu, M., & Liu, C. J., Improved Activity of Ru/$CeO_2$ Catalyst for $CO_2$ Methanation by Enhanced Electronic Metal–Support Interaction. *Energy & Fuels*, **39(1)**, 604-613, (2024).


## Reviewer: 3

*In the submitted manuscript, an ingenious concept that a novel strategy of "active nitrogen-mediated migration" is reported, which is different from the traditional pre-engineered carrier (bottom-up) or high-temperature physical migration (top-down) methods. The reactant ($NH_3$) itself is used as a chemical medium to dynamically construct a stable active interface under reaction conditions. However, there are still several issues to be clarified before suitable for publication.*

*1. The article claims that "top-down syntheses using active nitrogen species remain largely unexplored", but the review of the existing literature in the introduction is not comprehensive and in-depth enough. It is not possible to clearly define the essential differences and comparative advantages between its "active nitrogen-mediated" strategy and the known "chemical media" (such as $O_2$, CO, $H_2O$)-guided metal migration strategies. This led to the innovation of the work being exaggerated, and it seemed that it was just a "replacement" of a known concept (changing the medium from $O_2$/CO to $NH_3$). The author should revise the introduction to provide a more comprehensive and critical review of existing top-down strategies (including those induced by chemical media), and on this basis, accurately and humbly explain what the unique contribution of this work is. Was it the discovery of a new medium? Or is it discovered that this medium has a unique dual function of building "in situ" and stabilizing the interface in a specific reaction (ammonia decomposition)? A stronger literature base or more experimental data should be needed to support its innovative claims.*

**Answer)**

We thank the reviewer for this insightful comment regarding the positioning of our work within the broader context of top-down catalyst restructuring strategies. We humbly agree that a clearer discussion of previously reported chemically mediated metal migration processes is important for properly defining the novelty of this study. Accordingly, the introduction has been substantially revised to provide a more comprehensive overview of existing top-down strategies. Also, additional experimental data were collected, which will be shown in other following comments.

In the revised manuscript, we now discuss several representative chemically mediated restructuring approaches, including oxygen-induced atomic restructuring[1], hydrothermal water treatments enabling reversible metal dispersion[2], CO-induced strengthening of metal-support interactions[3], and $PH_3$ assisted conversion of nanoparticles into atomically dispersed species on g-$C_3N_4$[4]. In addition, previous studies employing ammonia-containing environments for metal dispersion such as ammonia treatment at 900 °C to mobilize Cu as $Cu(NH_3)_x$ and deposit on ZIF-8[5], or using dicyandiamide (DCD) to be decomposed to $NH_3$, leading to re-dispersion of metal particles into atomic structures[6, 7].

Importantly, we have clarified that the novelty of the present work does not lie simply in replacing previously used chemical media (e.g. $O_2$ or CO) with $NH_3$. Rather, the key distinction of this work is that the restructuring process is directly coupled to the catalytic reaction environment itself. In conventional top-down strategies, chemically mediated migration is typically performed as a separate pre-treatment step during catalyst synthesis. In contrast, in our system, the reactant ($NH_3$) continuously generates active nitrogen intermediates during ammonia decomposition, which promotes selective Ru migration toward $CeO_2$ domains under operating conditions.

As a result, the restructuring process simultaneously constructs and stabilizes a reaction-compatible Ru-$CeO_2$ interface during catalysis, rather than merely forming atomically dispersed metals during synthesis. We have revised the introduction accordingly to more clearly position our approach as a reaction-coupled restructuring strategy that integrates elements of both top-down and bottom-up catalyst design.

**[References]**


1 Jones, J. et al. Thermally stable single-atom platinum-on-ceria catalysts via atom trapping. *Science* **353**, 150-154 (2016).

2 Ryu, S.-H., Kim, S., Lee, H., Choi, J.-H. & Jeong, H. Robust palladium oxide nano-cluster catalysts using atomic ions and strong interactions for high-performance methane oxidation. *Nature Communications* **15**, 8348 (2024).

3 Jeong, H. et al. Fully dispersed Rh ensemble catalyst to enhance low-temperature activity. *Journal of the American Chemical Society* **140**, 9558-9565 (2018).

4 Zhou, P. et al. Thermolysis of noble metal nanoparticles into electron-rich phosphorus-coordinated noble metal single atoms at low temperature. *Angewandte Chemie International Edition*, **58**, 14184-14188 (2019).

5 Qu, Y. et al. Direct transformation of bulk copper into copper single sites via emitting

and trapping of atoms. *Nature Catalysis* **1**, 781-786 (2018).
6 Qu, Y. et al. Thermal emitting strategy to synthesize atomically dispersed Pt metal sites from bulk Pt metal. *Journal of the American Chemical Society* **141**, 4505-4509 (2019).
7 Zhang, E. et al. Bismuth single atoms resulting from transformation of metal–organic frameworks and their use as electrocatalysts for $CO_2$ reduction. *Journal of the American Chemical Society* **141**, 16569-16573 (2019).

**[Revised manuscript, page 4]**

In top-down strategies, metal species are initially deposited at random locations, followed by treatments that induce their migration and restructuring. Through such processes, the metal atoms relocate and stabilize into new, well-defined configurations under controlled conditions[22-25]. Wei *et al*. applied ultrahigh temperatures (~1000 °C) in an inert atmosphere to drive metal migration[26]. Several studies have demonstrated that specific chemicals can promote this transformation. For instance, Jones *et al*. used oxygen as a chemical mediator to direct atomic restructuring[27]. Hydrothermal treatment with water enabled temperature-dependent control of rhodium dispersion between atomic and clustered forms, while CO treatment induces mild reduction that strengthens palladium-support interactions[28,29]. $PH_3$ treatment was also applied to transform noble metal nanoparticles into a thermally stabilized metal single atoms on g-$C_3N_4$[30].

Similarly, ammonia has been explored as an effective medium for inducing atomic dispersion. Qu et al. treated Cu foam and ZIF-8 with ammonia at 900 °C to mobilize Cu as $Cu(NH_3)_x$ and deposited on ZIF-8 as single atom species[31]. Also, thermal annealing of the material with dicyandiamide (DCD) can induce similar effect, as DCD is decomposed into $NH_3$, leading to re-dispersion of metal particles into atomic structures[32,33]. While these approaches effectively enable atomic dispersion during catalyst synthesis, the restructuring processes are generally conducted as pre-treatment or fabrication steps and are not directly coupled with the catalytic reaction environment. Extending such chemically mediated migration strategies into systems where the reactant itself continuously generates active intermediates during operation may provide a more directly aligned approach for constructing and stabilizing reaction-compatible metal-support interfaces under working conditions.

In this study, we integrated key elements of both bottom-up and top-down strategies to design a catalyst whose interfacial structure can be established and maintained under reaction conditions. Ceria was selected as the oxide support component due to its well-known redox flexibility ($Ce^{3+}$ / $Ce^{4+}$), ability to form oxygen vacancies, and to have strong metal-support interactions that can stabilize dispersed metal species. However, the intrinsic advantages are not often fully realized when bulk ceria is used directly, as its redox properties and defect structure are strongly influenced by particle size and crystallinity[34,35]. One effective approach to modulate these properties is to control ceria domain size and structural order, which directly affect surface reducibility and vacancy concentration. To achieve this, we employed a high-surface-area carbon substrate as a dispersive framework for ceria domains. By controlling the annealing temperature, crystallinity, domain size, and surface defect density of ceria were tuned, enabling optimization of its surface redox characteristics. Ruthenium was deposited on the optimized support, and ammonia served as a chemically active nitrogen source to induce

atomic-scale restructuring. Unlike conventional top-down approaches, reactive nitrogen intermediates generated during ammonia decomposition drove the selective migration of Ru toward $CeO_2$ domains under reaction conditions. This reaction-coupled restructuring not only enabled redistribution but also stabilized a catalytically favorable Ru-$CeO_2$ interface. In contrast, hydrogen treatment produced randomly dispersed Ru nanoparticles. The resulting Ru-$CeO_2$ configuration suppressed hydrogen poisoning and improved compatibility with ammonia decomposition, leading to high hydrogen production under high-pressure conditions while maintaining structural stability after 100 hours of continuous operation.

*2. The core of the article is the "selective" migration of Ru atoms to $CeO_2$. However, TEM/EDS mapping (Figure 1), as the main evidence, has limited persuasiveness. Mapping shows that the Ru signal is enriched in the $CeO_2$ region after $NH_3$ treatment, but it cannot quantitatively prove that "Ru on carbon completely disappears", nor can it rule out the possibility of a small number of small clusters. This "selectivity" is more of a qualitative description and lacks statistical proof. More precise quantitative data must be provided, for example, line-scan EDS is used to quantitatively compare the Ru signal intensity in different areas (on $CeO_2$ particles, pure carbon area).*

**Answer)**

We sincerely thank the reviewer for this careful and constructive comment. We agree that the original HAADF-STEM/EDS mapping shown in **Fig. 1** mainly provides qualitative spatial information and, by itself, may not be sufficient to quantitatively demonstrate the redistribution of Ru species after $NH_3$ treatment. We also appreciate the reviewer's suggestion to provide additional analysis to more clearly evaluate the spatial distribution of Ru. To address this point, we performed additional line-scan EDS analysis across multiple representative regions of the catalysts, including (i) ceria-rich domains, (ii) carbon-ceria boundary regions, and (iii) carbon regions where bright contrast features were observed. The corresponding results are provided in **Fig. R1-R3**.

For Ru/$CeO_2$-C_$NH_3$, line-scan EDS performed across several ceria-containing regions consistently showed that the Ru and Ce signals appear together, indicating a strong spatial correlation between Ru and Ce in these areas. This observation suggested that, after $NH_3$ treatment, a substantial fraction of Ru species was migrated onto the $CeO_2$ domains. The same trend was observed in multiple analyzed regions, which provided additional support for the preferential interaction between Ru and $CeO_2$ inferred from the original mapping results.

At the same time, as noted by the reviewer, Ru-related signal was also detected in some carbon regions of the Ru/$CeO_2$-C_$NH_3$ sample. We also observed this during our analysis. However, several factors should be considered when interpreting these signals. The line-scan EDS measurements were conducted at an approximate 5 nm spatial scale, and therefore a certain level of signal fluctuation or background noise can occur, particularly for elements with relatively low loading such as Ru. To further examine the bright features occasionally observed in carbon regions of the ammonia-treated sample, we performed additional line-scan EDS analyses across these specific regions (**Fig. R2c**). In these areas, both Ru and Ce signals were detected, although the signal intensities were significantly lower than those observed on well-

defined ceria domains. It is also worth noting that Ce (Z = 58) produces stronger Z-contrast in HAADF-STEM than Ru (Z = 44). Therefore, bright contrast features observed on carbon regions may arise from trace Ce-containing species or overlapping Ce/Ru signals rather than exclusively from Ru atoms. In other words, in these regions the detected signals do not correspond to Ru alone. Ce-related signals are also present, making it difficult to unambiguously assign the bright features to Ru species solely based on HAADF contrast.

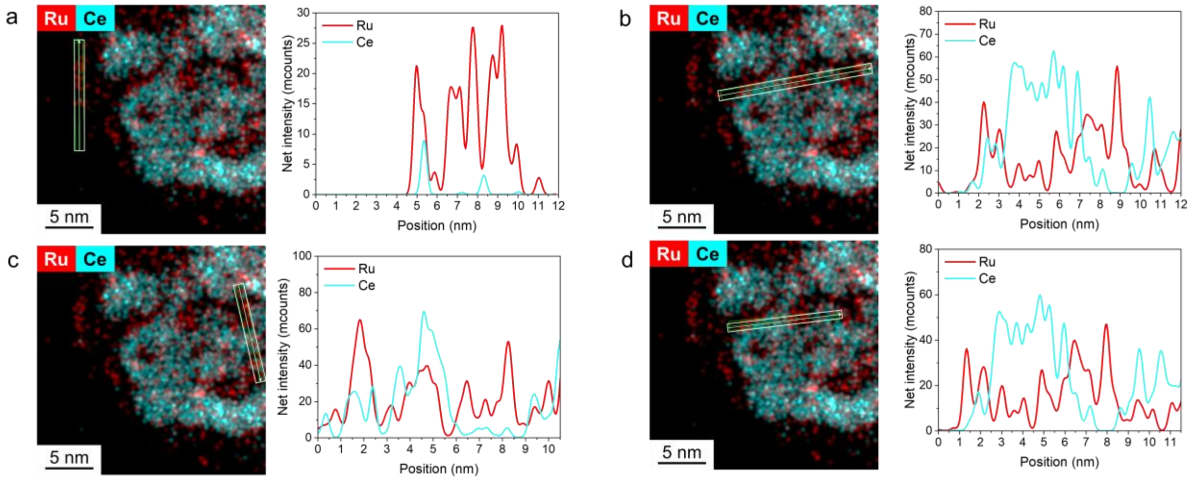


**Fig. R1** Line EDS mapping results for Ru/$CeO_2$-C_$N_2$.

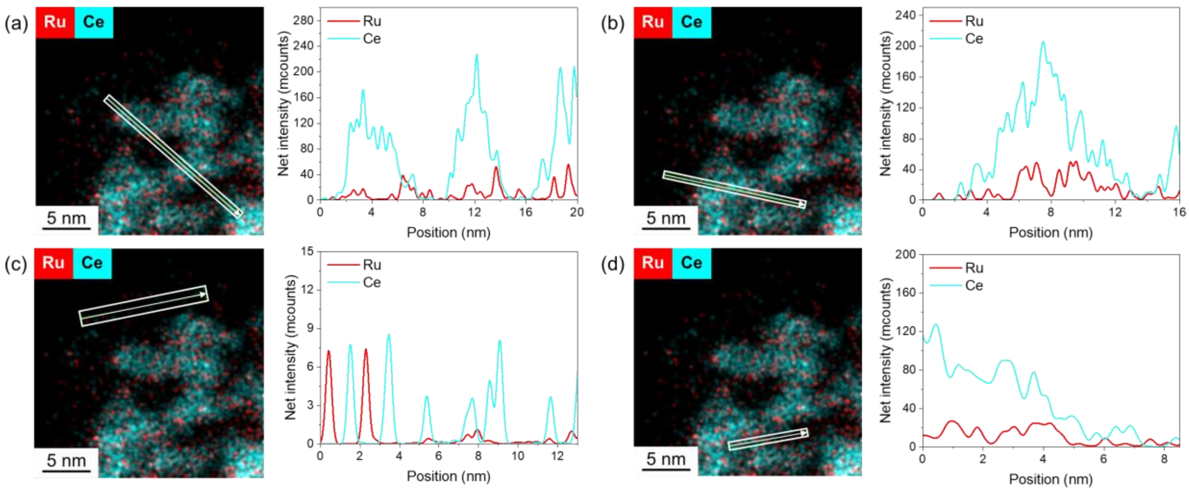


**Fig. R2** Line EDS mapping results for Ru/$CeO_2$-C_$NH_3$.

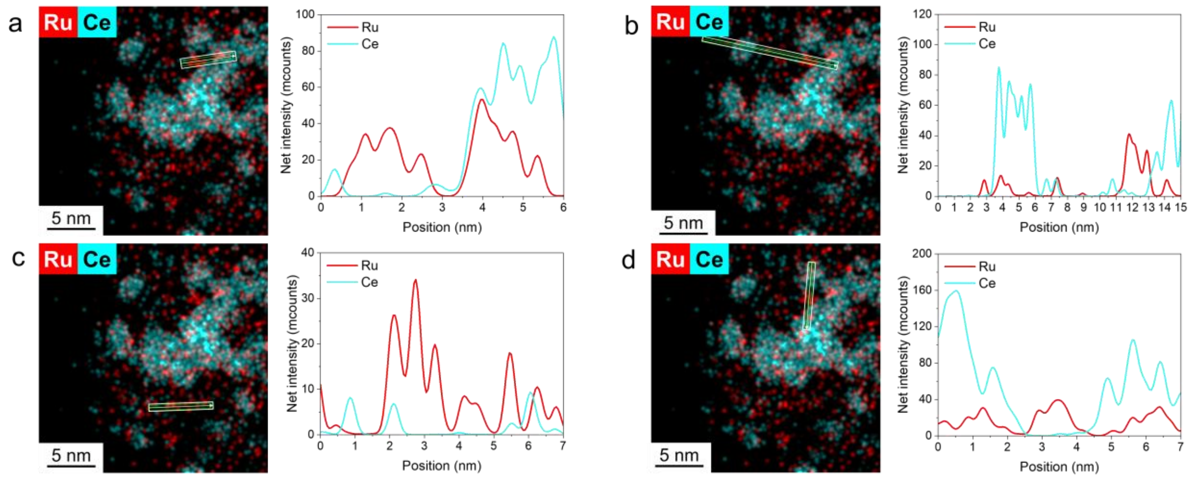


**Fig. R3** Line EDS mapping results for $Ru/CeO_2$-C_$H_2$.

In contrast, for $Ru/CeO_2$-C_$N_2$ and $Ru/CeO_2$-C_$H_2$, the line-scan EDS analyses frequently revealed regions where Ru signal appears without corresponding Ce signal, indicating that a significant portion of Ru species remained on the carbon substrate in these samples. Compared with these samples, the ammonia-treated catalyst exhibited a much stronger spatial correlation between Ru and Ce signals.

Based on these additional analyses, we agree with the reviewer that the original wording in the manuscript could be interpreted as implying complete removal of Ru from carbon. To improve the clarity and accuracy of the description, we revised the manuscript text to “After the $NH_3$ treatment, most of the ruthenium located on the carbon disappeared, and ruthenium selectively migrated toward the $CeO_2$ domains.”, rather than implying complete removal of Ru from carbon. We also stated that trace amounts of Ru, together with trace Ce species, may still remain on the carbon substrate, to provide a more balanced and transparent description of the observations.

Overall, while the redistribution should not be interpreted as absolute migration, the additional line-scan EDS analysis consistently indicate that $NH_3$ treatment leads to a much stronger preferential interaction of Ru with $CeO_2$ domains compared with $N_2$- and $H_2$- treated samples. These results were added in revised manuscript to clarify the selective immobilization of Ru on $CeO_2$. **Fig. R1-R3** were added as **Supplementary Fig. 6-8**, and corresponding manuscript was revised as shown below.

**[Revised manuscript, page 7]**

The spatial distribution and local structure of ruthenium were analyzed using ultrahigh-resolution double Cs-corrected TEM (UHR-TEM) with multichannel energy-dispersive X-ray spectroscopy (multi-EDS). The ruthenium species in $Ru/CeO_2$-C_$N_2$ were randomly distributed over both the $CeO_2$ and carbon domains. They existed either in an atomically dispersed form or as nanoparticles (Fig. 1a and Supplementary Fig. 6). After the $NH_3$ treatment, most of the ruthenium located on the carbon disappeared, and ruthenium selectively migrated toward the $CeO_2$ domains. Only a trace amount of Ru was still detected on the carbon regions together with Ce signals, although the corresponding intensities were significantly lower than

those observed on ceria domains (Fig. 1b and Supplementary Fig. 7). In contrast, the $H_2$ treatment formed randomly located Ru nanoparticles on both carbon and $CeO_2$, with mixed sizes (Fig. 1c and Supplementary Fig. 8). Supplementary Fig. 9 shows additional TEM images. To further examine the structure of Ru species on $CeO_2$, UHR-TEM images were taken at higher magnification (Supplementary Fig. 10). The $CeO_2$ lattice was clearly resolved, and no Ru nanoparticles or sub-nanometer clusters were observed. Instead, some isolated bright features were detected along with the ceria domains, consistent with atomically dispersed Ru species. These results suggest that $NH_3$ treatment enables selective migration with atomic dispersion of ruthenium onto $CeO_2$, whereas $H_2$ treatment leads to aggregation into small clusters or nanoparticles at random locations. The deposition of ruthenium on $CeO_2$ and carbon and treatment with $NH_3$ caused it to exist as nanoparticles (Supplementary Fig. 11).

*3. The mentioned "(Ru) atoms onto ceria ($CeO_2$) domains, forming a durable single atom structure" in the abstract and the subheading "Selective migration of Ru atoms onto $CeO_2$ into single atom structure" under "RESULTS AND DISCUSSION" part, both illustrate the existence of Ru single atom in the $CeO_2$, the single-atom dispersion of Ru on the $CeO_2$ surface should be directly observed and counted through atomic resolution STEM-HAADF, and compared with the sample before treatment. Moreover, the difference on the state of catalytic materials in ammonia among that in hydrogen and nitrogen is originated from the participation in the process of ammonia decomposition reaction. Such migration and change process of ruthenium atoms is also highly probably caused by the dynamic reconstruction of catalyst during the ammonia decomposition reaction. Therefore, it is suggested that the in-situ transmission electron microscopy test of Ru/CeC under heated ammonia atmosphere should be supplied to intuitively prove the migration and change process of ruthenium atoms in ammonia, making the analysis more convincing.*

**Answer)**

We sincerely thank the reviewer for this important comment regarding the direct visualization of atomically dispersed Ru species. Following the reviewer's suggestion, we carefully re-examined the catalyst using ultrahigh-resolution HAADF-STEM imaging at the highest attainable magnification (**Fig. R4**). The updated images have been added to the revised manuscript as **Supplementary Fig. 10**. Under these imaging conditions, the ceria lattice was clearly resolved with high crystallinity, indicating that atomic-scale structural features of the $CeO_2$ support could be reliably observed.

Importantly, no Ru nanoparticles or sub-nanometer clusters were observed on the $CeO_2$ surface in the ammonia-treated sample. If Ru were present as nanoparticles or small clusters, they would appear as distinct high-contrast features outside the ceria lattice. Instead, several isolated bright contrast features located on or within the ceria lattice can be identified. These features are highlighted with yellow circles in the revised HAADF-STEM images to guide the reader. We note that direct identification of individual Ru atoms on $CeO_2$ using HAADF-STEM is intrinsically challenging due to the limited Z-contrast difference between Ru (Z = 44) and Ce (Z = 58). Because HAADF intensity scales approximately with atomic number, the heavier cerium atoms in the ceria lattice generate significantly stronger contrast than Ru. As a result,

isolated Ru atoms on $CeO_2$ can appear only as subtle bright features within the already bright ceria lattice, making their identification considerably more difficult than in systems where metals are supported on lighter substrates.

Despite this intrinsic limitation, the combination of ultrahigh-resolution HAADF-STEM imaging and EDS mapping provided consistent evidence that Ru species are highly dispersed and preferentially associated with the $CeO_2$ domains. In particular, the corresponding EDS maps showed that the Ru signal was strongly localized in the ceria regions, while no Ru clusters were observed. Furthermore, the XAFS analysis presented in **Fig. 2a, Supplementary Fig. 12,** and **Supplementary Table 2** supported this interpretation. The EXAFS fitting results showed the dominant Ru-O coordination, which was consistent with atomically dispersed Ru structure on the oxide support.

Taken together, the absence of detectable Ru clusters in HAADF-STEM, the spatial correlation of Ru and Ce signals in EDS mapping, and the Ru-O coordination revealed by XAFS collectively support the presence of atomic Ru species on $CeO_2$ domains after ammonia treatment.

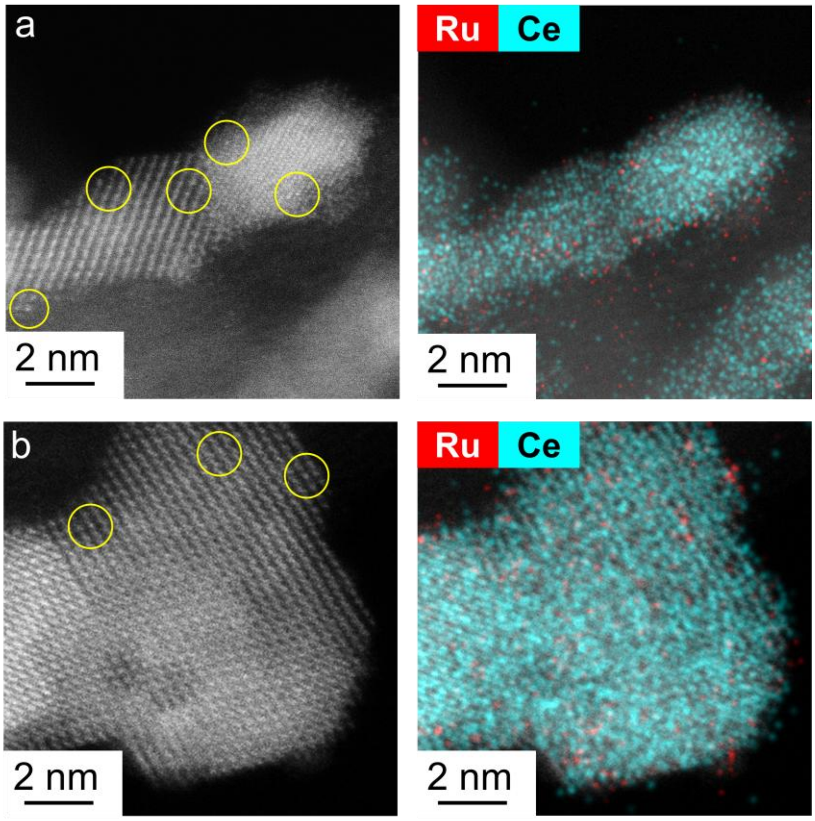


**Fig. R4** UHR-STEM images of Ru/$CeO_2$-C_$NH_3$ in 2 nm scale resolution. Sites for atomic Ru were highlighted with yellow circles.

We also appreciate the reviewer's suggestion to directly observe the migration of Ru species under ammonia atmosphere using in-situ TEM. Such an approach could indeed provide valuable insight into the dynamic restructuring process. However, performing in-situ TEM under flowing $NH_3$ at elevated temperatures presented significant experimental limitations. The introduction of $NH_3$ into the TEM column may substantially reduce imaging resolution due to

strong electron scattering and contamination effects. In addition, we consulted several open in-situ TEM facilities in South Korea regarding the feasibility of this experiment, and were informed that introducing ammonia gas into the TEM system is not permitted due to safety and instrument contamination issue. Therefore, although we carefully considered the reviewer's suggestion, direct in-situ TEM observation under $NH_3$ could not be implemented in the present study.

Instead, to obtain insight into the structural evolution during ammonia treatment, we performed a time-resolved ex-situ TEM analysis using UHR-TEM and multi-EDS detectors (**Fig. R5**). In this approach, catalysts were exposed to $NH_3$ at 500 °C for different times (1, 5, 10, 20, and 40 minutes), followed by rapid quenching and sampling. This method allowed us to capture intermediate structural states during the ammonia-induced restructuring process. The time-resolved TEM results revealed a clear temporal evolution of Ru species. At short treatment times (1 and 5 min), Ru species remain widely distributed, with a significant fraction still located on the carbon phase. A notable transition occurred between 5 and 10 min, where the number of Ru particles observed on carbon decreased, accompanied by a substantial reduction in the Ru signal detected from carbon regions in EDS analysis. With further treatment, this trend became more pronounced. After 40 min, Ru signals from EDS mapping were predominantly localized around $CeO_2$ domains. Although weak residual signals of Ru and Ce were occasionally detected in carbon regions, these signals were likely associated with trace amount of Ru, background or projection effects rather than discrete Ru species (**Fig. R2**). Finally, after 1 hour of ammonia treatment, the structure appeared to be stabilized, with most of Ru making interaction with $CeO_2$, as shown in **Fig. 1b** of the original manuscript.

Taken together, these time-resolved results provided consistent evidence for ammonia-induced migration of Ru species to $CeO_2$ domains. While this approach did not constitute "in-situ" TEM observation, this time-resolved analysis enabled us to capture the progressive structural evolution during ammonia treatment within the experimental constraints of the system. These findings support our conclusion that the dynamic restructuring of Ru species under ammonia is a key factor governing the formation of the active Ru-$CeO_2$ interfacial configuration. Accordingly, **Fig. R5** was added as **Supplementary Fig. 18** in revised manuscript with corresponding description.

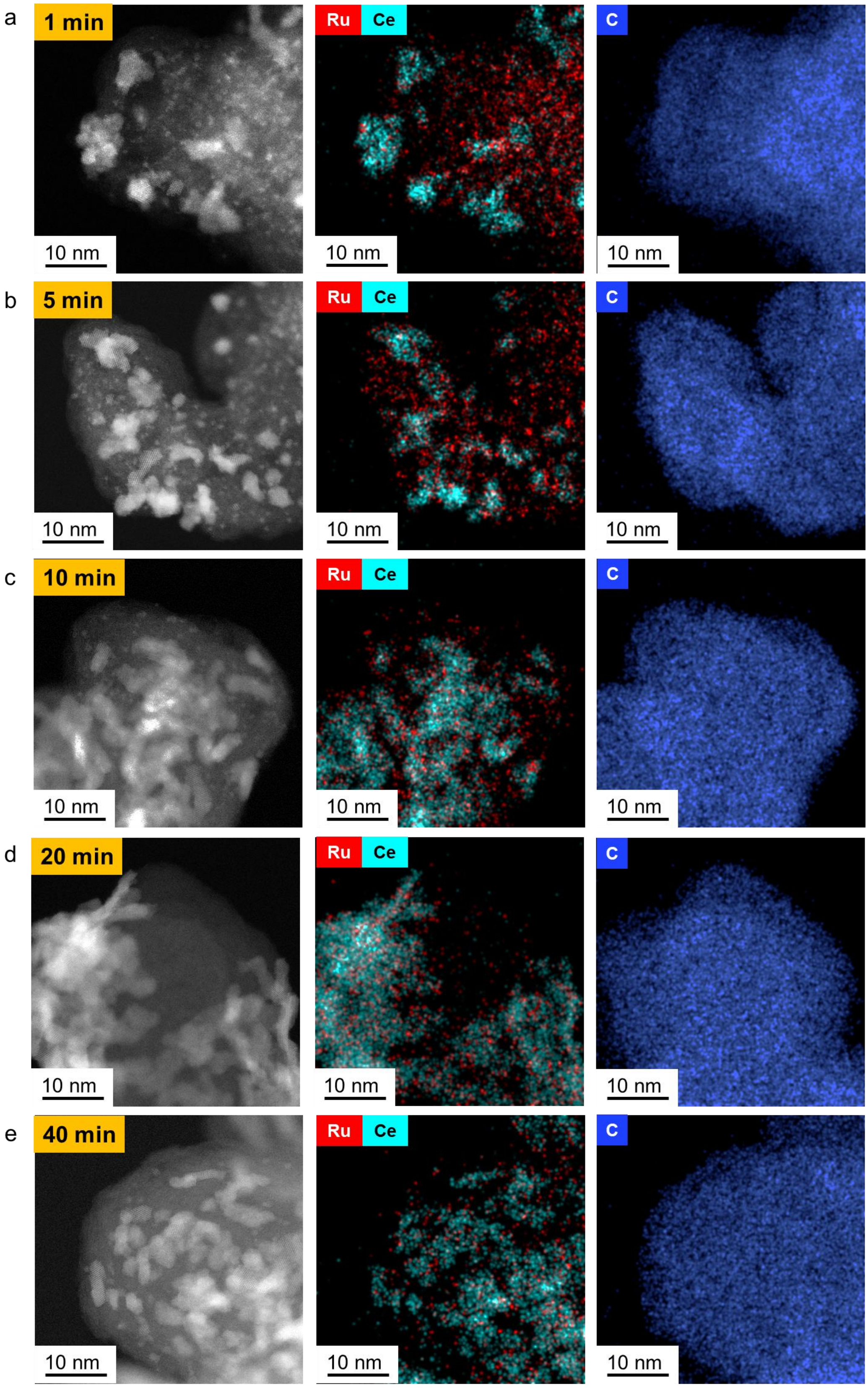


**Fig. R5** Time-resolved UHR-TEM images and corresponding multi-EDS results. Images were taken after $NH_3$ treatment on Ru/$CeO_2$-C_$N_2$ for 1 min, 5 min, 10 min, 20 min, and 40 min at 500 °C.

**[Revised manuscript, page 13]**

To further resolve the temporal evolution of this process, time-resolved UHR-TEM and N 1s XPS analyses were conducted. Ru/$CeO_2$-C_$N_2$ was exposed to $NH_3$ at 500 °C for varying durations (1, 5, 10, 20, and 40 min), followed by rapid quenching (Supplementary Fig. 18). At early stages (1 and 5 min), Ru species remained broadly distributed, including on carbon domains. A pronounced redistribution occurred between 5 and 10 min, where Ru signals on carbon decreased and became increasingly localized on $CeO_2$ domains. With prolonged treatment, Ru species were predominantly associated with $CeO_2$, indicating structural stabilization. Consistently, time-resolved N 1s XPS results showed that nitrogen species were not detected at early stages but emerged from ~10 min onward, coinciding with the onset of Ru migration (Supplementary Fig. 19). Collectively, these findings demonstrate that nitrogen-containing intermediates generated during $NH_3$ decomposition not only mobilize ruthenium, but also direct its relocation to the $CeO_2$ domains, thereby promoting the formation of Ru-$CeO_2$ interfaces.

*4. The article claims that the catalyst is structurally stable after 100 hours of high-pressure testing, but the evidence provided is completely insufficient to support this key conclusion. The EXAFS fitting data of the sample after reaction must be provided, and the exact coordination number is used to prove that the Ru-Ru coordination has not increased. Otherwise, the conclusion of "structural preservation" is arbitrary. The Ru 3p XPS, Ce 3d XPS and Ru K-edge XANES data of the catalyst after the reaction must be supplemented and strictly compared with the data before the reaction.*

**Answer)**

We thank the reviewer for this important comment. We agree that the structural stability of the catalyst after long-term high-pressure reaction must be supported by more structural and spectroscopic evidence. In response, we included additional post-reaction characterizations, including EXAFS fitting, Ru K-edge XANES, Ru 3p XPS. The results consistently supported the structural preservation of the catalyst after 100 h of high-pressure ammonia decomposition (**Fig. R6**).

First, EXAFS fitting analysis was conducted for both catalysts after the long-term high-pressure reaction. For Ru/$CeO_2$-C_$NH_3$, the coordination numbers were determined to be Ru-O 3.9 and Ru-Ru of 0.2, whereas for Ru/$CeO_2$-C_$H_2$, Ru-O coordination number was 3.4 and Ru-Ru was 1.4 (**Fig. R6a, b,** and **Table R1**). These results clearly showed that the ammonia-treated catalyst maintains a dominant Ru-O coordination with negligible Ru-Ru coordination even after 100 h reaction. Importantly, the Ru-Ru coordination number remained extremely low, indicating that no significant aggregation or nanoparticle growth occurred during the reaction. In contrast, the $H_2$-treated catalyst retained a noticeable Ru-Ru coordination, consistent with its nanoparticle nature. While a slight increase in Ru-O coordination number is observed compared to the fresh catalyst, the overall coordination trend remains unchanged, confirming that the fundamental local structure of Ru species is preserved.

Second, Ru K-edge XANES spectra after reaction showed negligible changes compared to

the fresh catalysts (**Fig. R6c** and **Fig. 2**), indicating that the oxidation state of Ru is well maintained during long-term operation. Notably, both catalysts retained their original spectral features without significant modification after the reaction. Third, Ru 3p XPS analysis was additionally performed. After the long term reaction, a slight positive shift (~ 0.4 eV) in binding energy was observed, suggesting a marginal increase in oxidation state. However, the relative difference between catalysts was preserved, and no evidence of metallic Ru formation or significant reduction was detected (**Fig. R6d**). Fourth, Ce 3d XPS analysis revealed that the $CeO_2$ support structure was also largely preserved (**Fig. R6e, f**). Notably, Ru/$CeO_2$-C_$NH_3$ maintained its initial defect structure, whereas Ru/$CeO_2$-C_$H_2$ showed partial generation of $Ce^{3+}$ species after reaction, indicating some degree of support restructuring only in the $H_2$-treated system. These observations suggest that ammonia treatment promotes a redox-active $CeO_2$ surface without degrading the support structure.

Taken together with previously obtained UHR-TEM, Raman and XRD results (**Supplementary Fig. 29** and **Fig. R6g, h**), these findings provide consistent and quantitative evidence that (i) Ru remains atomically dispersed, (ii) the oxidation state of Ru is preserved, and (iii) the Ru-$CeO_2$ interfacial structure is maintained after long-term reaction of Ru/$CeO_2$-C_$NH_3$. These observations were fully consistent with the stable catalytic performance observed during the 100 h test and confirmed that no significant structural degradation occurred under high-pressure ammonia decomposition conditions.

Accordingly, we have added the EXAFS fitting results, post-reaction XANES spectra, and XPS analyses (Ru 3p and Ce 3d) to the revised manuscript and Supplementary Information to provide direct and quantitative support for the structural durability of the catalyst. Especially, **Fig. R6** was added as **Supplementary Fig. 28** and **Table R1** was added as **Supplementary Table 5**.

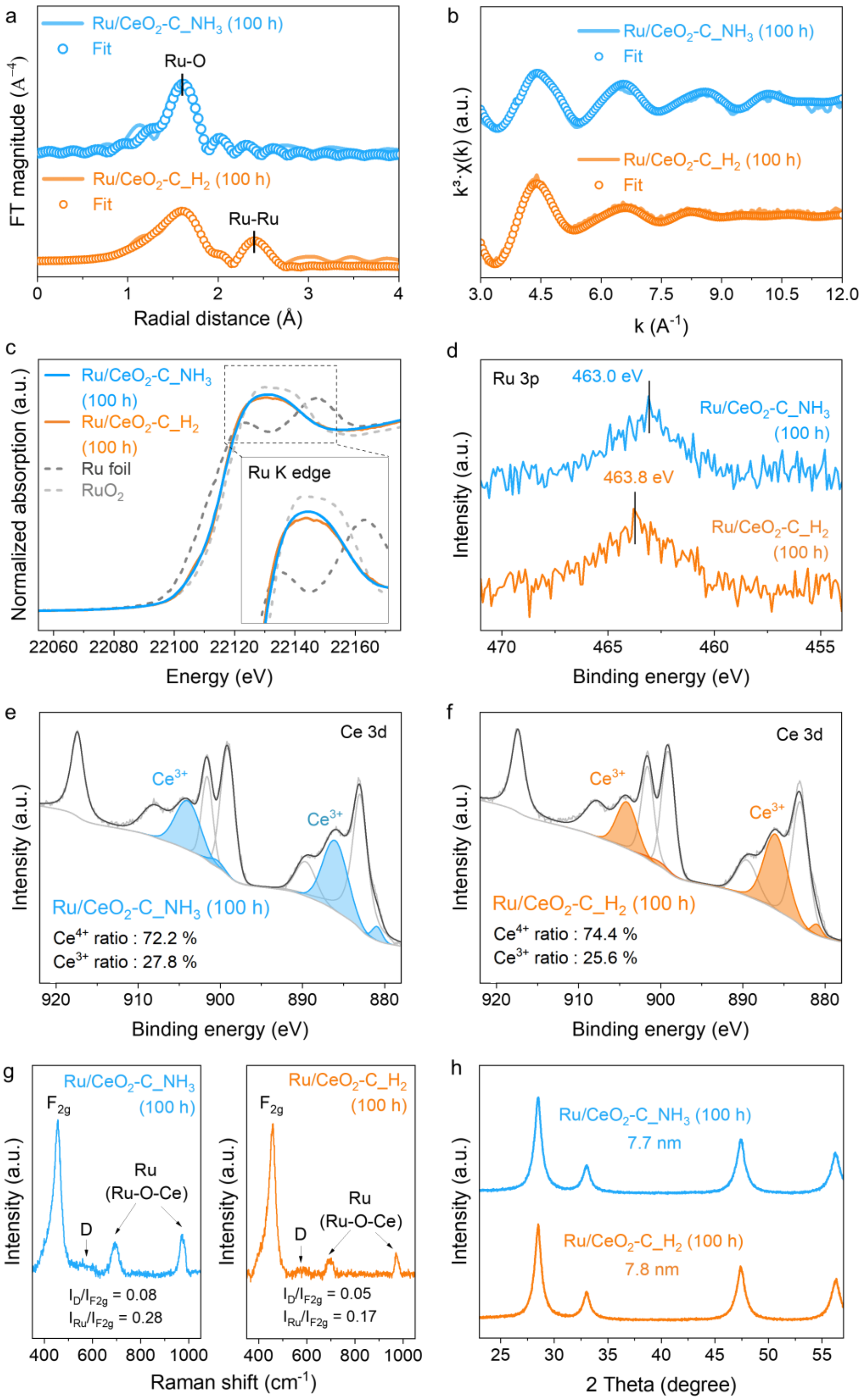


**Fig. R6** EXAFS fitting results of Ru K edge for Ru/$CeO_2$-C_$NH_3$ and Ru/$CeO_2$-C_$H_2$ after high-pressure long term durability test in (a) R space, and (b) K space. The solid lines indicate experimental data, and the dots denote the fitting results. (c) XANES, (d-f) Ru 3p and Ce 3d XPS, (g) Raman, and (h) XRD results of Ru/$CeO_2$-C_$NH_3$ and Ru/$CeO_2$-C_$H_2$ after high-pressure long term durability test.

**Table R1** Best-fit values for the EXAFS results of Ru/$CeO_2$-C_$NH_3$ and Ru/$CeO_2$-C_$H_2$ after 100 h of long-term reaction at high pressure.

| Sample | Path | Coordination number | Debye-Waller Factor [$\sigma^2 \cdot 10^{-3}$ / Å$^2$] | R [Å] | R-factor |
|---|---|---|---|---|---|
| Ru/$CeO_2$-C_$NH_3$ (100 h) | Ru-O | 3.9 ± 0.6 | 5.6 ± 1.9 | 2.0 ± 0.1 | 0.028 |
| | Ru-Ru | 0.2 ± 0.2 | 3.0* | 2.6 ± 0.1 | |
| Ru/$CeO_2$-C_$H_2$ (100 h) | Ru-O | 3.4 ± 0.4 | 6.5 ± 1.6 | 2.0 ± 0.1 | 0.024 |
| | Ru-Ru | 1.4 ± 0.5 | 6.8 ± 2.0 | 2.6 ± 0.1 | |

* This factor was fixed during the EXAFS fitting

**[Revised manuscript, page 19]**

The catalysts were investigated after long term reaction at high pressure to confirm structure stability. The EXAFS analysis of the Ru/$CeO_2$-C_$NH_3$ after durability test showed that the Ru-O coordination remains high (3.9), while the Ru-Ru coordination remains negligible (0.2), indicating that no significant aggregation of Ru species occurred during reaction. In contrast, the Ru/$CeO_2$-C_$H_2$ after durability test exhibited a substantially higher Ru-Ru coordination (1.4), consistent with the presence of Ru nanoparticles. These results demonstrated that the atomic Ru structure formed after $NH_3$ treatment was preserved even under prolonged high-pressure ammonia decomposition (Supplementary Fig. 28a, b, and Supplementary Table 5). Furthermore, Ru K-edge XANES spectra showed negligible changes before and after reaction, indicating that the oxidation state of Ru was well maintained (Supplementary Fig. 28c). Ru 3p XPS analysis exhibited slight shift to higher binding energy (~0.4 eV), without any evidence of metallic Ru formation, while preserving the relative difference between catalysts (Supplementary Fig. 28d). In addition, Ce 3d XPS and Raman analysis confirmed that the ceria structure and the Ru-$CeO_2$ interaction remain largely unchanged after reaction (Supplementary Fig. 28e-g). XRD results for the Ru/$CeO_2$-C-based catalysts after 100 h of reaction showed that the $CeO_2$ domains did not aggregate or sinter during the operation (Supplementary Fig. 28h). Finally, the UHR-TEM and multi-EDS results confirmed that the ruthenium in Ru/$CeO_2$-C_$NH_3$ remained atomically dispersed on $CeO_2$ domains even after the long-term high-pressure reaction (Supplementary Fig. 29a). In contrast, the ruthenium in Ru/$CeO_2$-C_$H_2$ still existed as nanoparticles randomly distributed across the support without preferential interaction with $CeO_2$ (Supplementary Fig. 29b).

*5. Simply attributing the N 1s XPS peak (~398.7 eV) to "nitrogen incorporation into the $CeO_2$ lattice" is hasty. This binding energy may also correspond to adsorbed nitrogen species (e.g. -NH2, -NH) or surface nitrides rather than bulk doping. The authors do not provide any higher-level spectroscopic evidence (such as N K-edge XANES) or structural evidence (such as changes in lattice parameters in XRD) to support the strong conclusion of "lattice doping".*

**Answer)**
This comment is answered together with *Reviewer's comment 6.*

*6. The interpretation of the N 1s XPS peak should be more cautious, and it is recommended to change it to "nitrogen-containing species are preferentially incorporated/adsorbed on $CeO_2$". It is strongly recommended to try to obtain N K-edge soft X-ray absorption spectrum, which can clearly distinguish the chemical environment of nitrogen (such as π bond, σ bond) and provide decisive evidence for the existence form of nitrogen. The DFT calculation only considers the adsorption of Ru-$NH_2$. The adsorption energy of Ru atoms on the N-doped $CeO_2$ surface should be additionally calculated and compared with the pure $CeO_2$ surface to directly verify the hypothesis of "nitrogen doping guided anchoring".*

**Answer)**
We thank the reviewer for these thoughtful comments regarding the interpretation of the N 1s XPS results and the nature of nitrogen species in our catalyst. We acknowledge that assigning the N 1s peak solely to "nitrogen incorporation into the $CeO_2$ lattice" may be hasty, as this binding energy region can include multiple nitrogen configurations, such as $NH_x$ species, surface-bound nitrogen species, or other non-bulk nitrogen environments. Our intention was not to claim definitive substitutional lattice doping, but rather to describe the presence of nitrogen species associated with $CeO_2$ domains under high-temperature $NH_3$ treatment conditions. Accordingly, we have revised the manuscript to adopt a more accurate and cautious description as suggested, to "nitrogen-containing species preferentially incorporated/adsorbed on $CeO_2$ domains."

In addition, to avoid overinterpretation of the N 1s XPS results, we have revised the peak assignment scheme in the manuscript. The N 1s spectrum is now deconvoluted into three components to N-1 (398.3 eV), N-2 (400.1 eV), and N-3 (402.3 eV). The N-1 peak is assigned to substitutional nitrogen (i.e., N replacing lattice oxygen) and/or surface NH species, the N-2 peak to interstitial nitrogen species and/or $NH_2$ species, and the N-3 peak to chemisorbed $NH_3$, $NH_4^+$ species, or oxidized nitrogen species (e.g., $NO_x$)[1-5]. This revised assignment explicitly reflected the possible coexistence of multiple nitrogen configurations and avoids attributing the observed N 1s peak to a single, specific nitrogen species.

To further clarify the nature of nitrogen species and directly address the reviewer's concern, we performed additional N K-edge soft X-ray absorption measurements with construction of a reference system for comparison. Specifically, N-doped $CeO_2$ was synthesized via urea thermolysis under $N_2$ (following *Zhan et al., Applied Catalysis B, 2023*)[6], which is known to have dominant interstitial nitrogen species. The N 1s XPS of this reference sample shows a dominant peak at 400.1 eV, consistent with our catalyst. However, its N K-edge spectrum

exhibited a clear σ*-dominant feature with negligible π* contribution, providing a characteristic spectral fingerprint of interstitial nitrogen in $CeO_2$ (**Fig. R7a**).

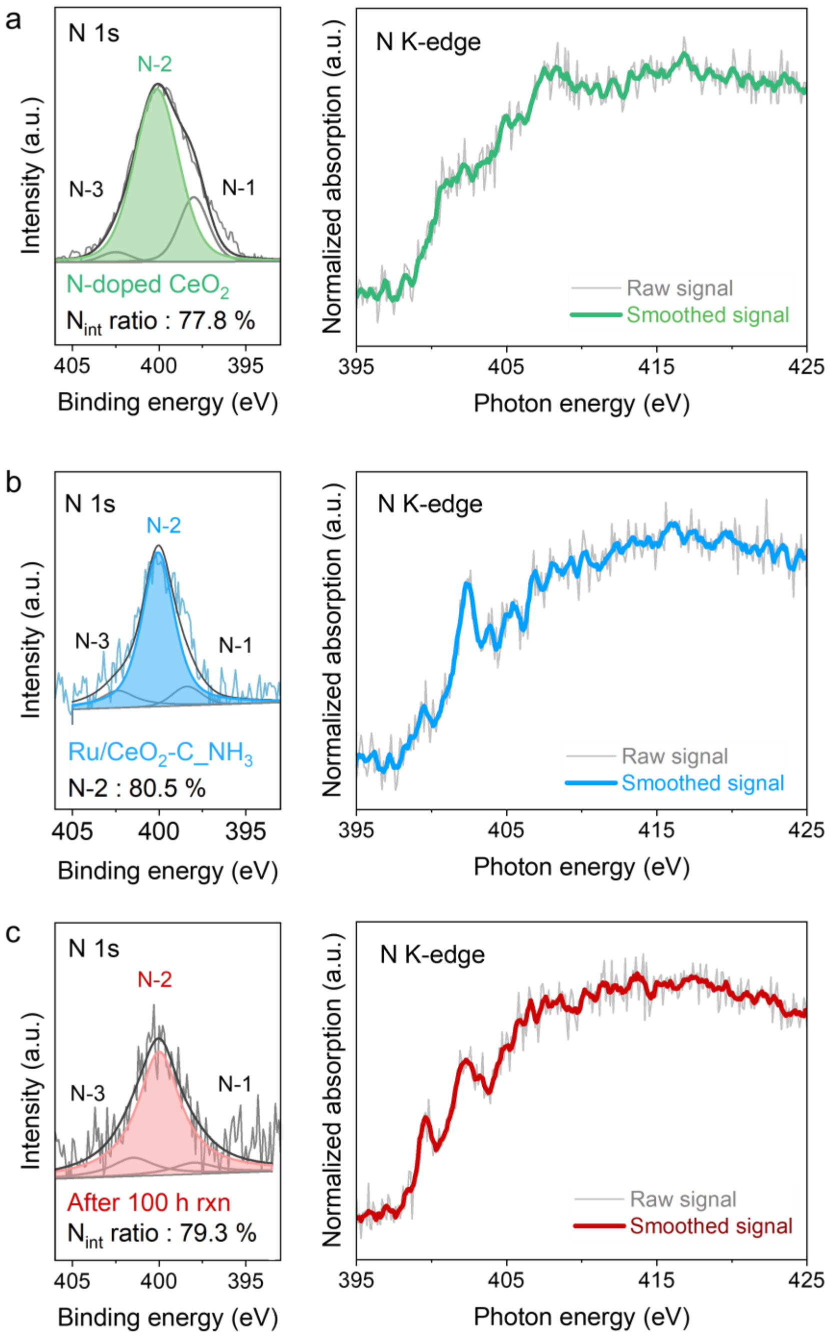


**Fig. R7** N 1s XPS and N K-edge X-ray absorption spectroscopy results of (a) N-doped $CeO_2$, (b) $Ru/CeO_2$-C_$NH_3$, and (c) $Ru/CeO_2$-C_$NH_3$ after 100 h of reaction.

In contrast, the $Ru/CeO_2$-C_$NH_3$ catalyst exhibited a markedly different N K-edge spectrum, characterized by a pronounced π* feature at 402.2 eV and a broad, less defined σ* region (**Fig. R7b**)[7-9]. This indicated that, although the N 1s XPS result was similar, the nitrogen species in $Ru/CeO_2$-C_$NH_3$ were not equivalent to those in the N-doped $CeO_2$ reference. Instead, these

features were consistent with more electronically hybridized nitrogen species, likely originating from surface chemisorbed $*NH_2$ on $CeO_2$ domains.

To further investigate the evolution of nitrogen species under reaction conditions, we analyzed Ru/$CeO_2$-C_$NH_3$ after 100 h of ammonia decomposition. While the N 1s XPS result remains similar, the N K-edge spectrum showed a clear change. The intensity of the $\pi^*$ feature at 402.2 eV decreases, while the contribution at 399.8 eV becomes more pronounced (**Fig. R7c**). Importantly, although the spectral change occurred mainly within the $\pi^*$ region rather than through the emergence of a fully $\sigma^*$-dominant spectrum, the overall peak shifted toward that of the interstitial-N-dominant N-doped $CeO_2$ reference. These results suggest that nitrogen species are initially formed as chemisorbed $*NH_2$, but undergo partial evolution toward a more stabilized, lattice-associated interstitial configuration during reaction.

To support this interpretation, we performed additional DFT calculations as suggested by the reviewer. We constructed models of N-doped $CeO_2$ (111) with nitrogen located at interstitial sites and evaluated their stability. To model the stable N-doped $CeO_2$(111) system, and to reflect the experimental observation of interstitial nitrogen, three potential sites of N were simulated. Case 1 represented nitrogen atom located at the Ce-Ce-O site on the surface of $CeO_2$(111) slab, case 2 represented nitrogen atom located at the Ce-O-Ce-O site, and case 3 represented nitrogen atom located at the Ce-Ce-O site on the subsurface of $CeO_2$(111) slab. Among the considered configurations, surface interstitial (case 1 in **Fig. R8**) was found to be the most stable (nitrogen binding energy of -2.45 eV), compared to subsurface configurations.

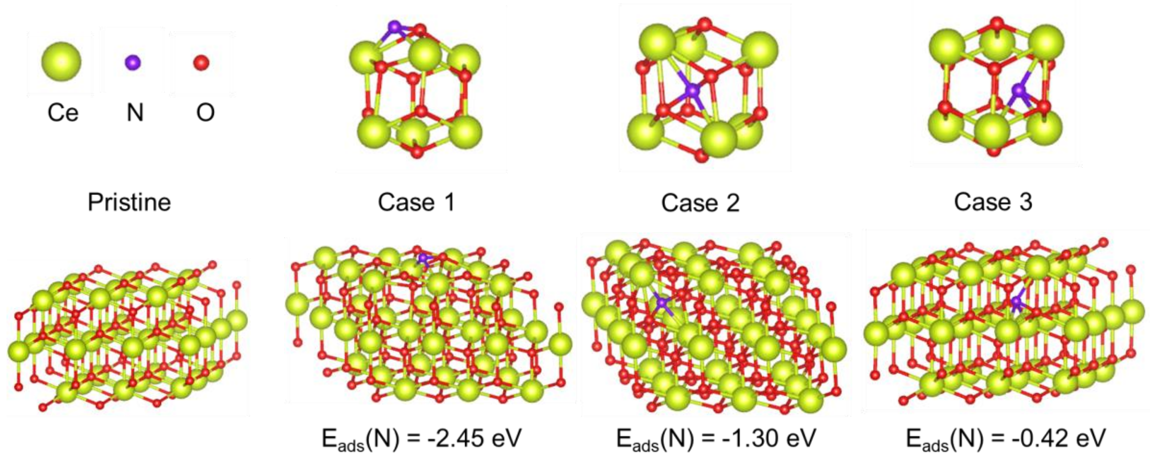


**Fig. R8** The atomic configuration and binding energy of N atom for three potential sites for N-doping. Case 1 for surface interstitial N stabilization, case 2 and 3 for subsurface interstitial N stabilization.

We then calculated the adsorption energy of a single Ru atom on both pristine and N-doped $CeO_2$ surfaces (**Fig. R9**). Adsorption energy of Ru atom on the surface was calculated on the N-doped $CeO_2$(111) and pristine $CeO_2$(111), according to the equation shown below.

$$E_{ads}(Ru) = E(Ru/substrate) - E(substrate) - E(Ru)$$

Where, E(Ru/substrate), E(substrate), and E(Ru) denotes the total energy of a Ru-adsorbed substrate (pristine or N-doped $CeO_2$ (111) slab), the total energy of bare substrate, and the total energy of an isolated single Ru atom at vacuum system. The adsorption energy was significantly enhanced from -4.97 eV (Ru adsorption on pristine $CeO_2$) to -6.58 eV (Ru adsorption on N-doped $CeO_2$), indicating a substantially stronger interaction between Ru and the N-modified surface. These results suggest that nitrogen species, particularly interstitial-like configurations can significantly enhance the anchoring of Ru species on $CeO_2$.

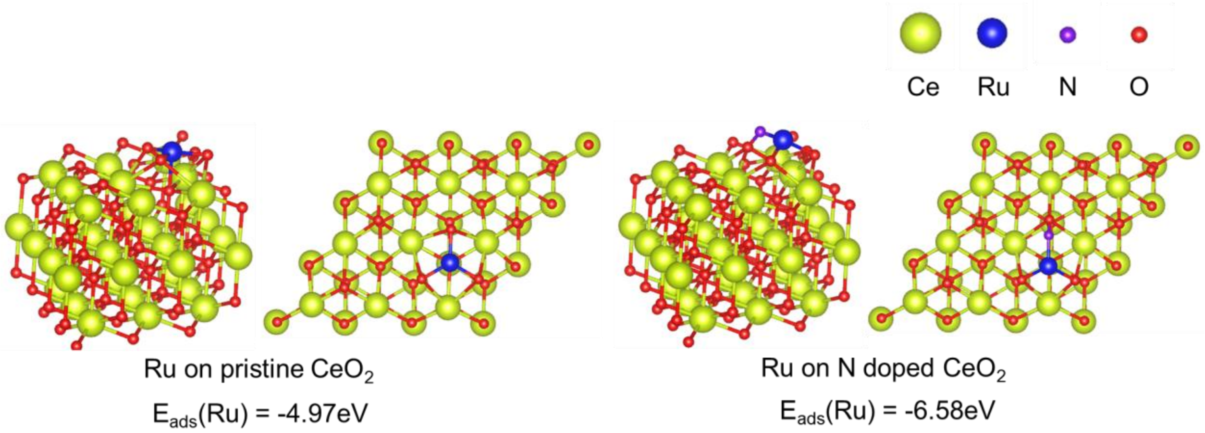


**Fig. R9** Adsorption energy calculation of a single Ru atom on the surface pristine $CeO_2$ (111) and N-doped $CeO_2$ (111).

Taken together, we agree that the original interpretation based solely on N 1s XPS was too strong, and we have revised the manuscript accordingly. Rather than assigning a single nitrogen configuration, our results now support a more comprehensive picture with N 1s XPS, N K-edge results, and additional DFT-calculation results. According, **Fig. R7** was added as **Supplementary Fig. 20**, and **Fig. R8 and Fig. R9** were added as **Supplementary Fig. 23**.

**[References]**

1 Sandoval-Díaz, L. et al. Decoding technical multi-promoted ammonia synthesis catalysts. *Nature Communications* **16**, 7820 (2025).
2 Zhan, S. et al. Controllable synthesis of substitutional and interstitial nitrogen-doped ceria: The effects of doping sites on enhanced catalytic ozonation of organic pollutants. *Applied Catalysis B: Environmental* **321**, 122040 (2023).
3 Paick, J. et al. Effective atomic N Doping on $CeO_2$ nanoparticles by environmentally benign urea thermolysis and its significant effects on the scavenging of reactive oxygen radicals. *ACS Omega* **8**, 22646-22655 (2023).
4 Ben Yaala, M. et al. Plasma-assisted catalytic formation of ammonia in $N_2$–$H_2$ plasma on a tungsten surface. *Physical Chemistry Chemical Physics* **21**, 16623-16633 (2019).
5 Egawa, C., Naito, S. & Tamaru, K. Adsorption and decomposition of ammonia on W(100); XPS and UPS studies. *Surface Science* **131**, 49-60 (1983).
6 Zhan, S. *et al.* Controllable synthesis of substitutional and interstitial nitrogen-doped ceria: The effects of doping sites on enhanced catalytic ozonation of organic pollutants. *Applied Catalysis B: Environmental* **321**, 122040 (2023).

7 Park, K. et al. Structural effects of nitrogen-doped titanium oxide supports on stabilization of ruthenium active species in carbon dioxide hydrogenation to formate. *Applied Catalysis B: Environmental* **335**, 122873 (2023).

8 Soriano, L., Abbate, M., Pen, H., Czyżyk, M. T. & Fuggle, J. C. The interaction of N with Ti and the oxidation of TiN studied by soft X-ray absorption spectroscopy. *Journal of electron spectroscopy and related phenomena* **62**, 197-206 (1993).

9 Lee, D. K., Choi, J. I., Lee, G. H., Kim, Y. H. & Kang, J. K. Energy states of a core-shell metal oxide photocatalyst enabling visible light absorptions and utilization in solar-to-fuel conversion of carbon dioxide. *Advanced Energy Materials* **6** (2016).


**[Revised manuscript, page 12]**

N 1s XPS measurements were conducted to determine the nitrogen incorporation after treatment. As shown in Fig. 3c, nitrogen doping was observed only in the samples containing $CeO_2$ (Ru/$CeO_2$-C, Ru/$CeO_2$, and $CeO_2$-C), whereas no significant nitrogen signals were detected for Ru/C or the bare carbon support. The N 1s spectrum was deconvoluted into three components N-1 (398.3 eV), N-2 (400.1 eV), and N-3 (402.3 eV). The N-1 peak is assigned to substitutional nitrogen (i.e., N replacing lattice oxygen) and/or surface NH species, the N-2 peak to interstitial nitrogen species and/or $NH_2$ species, and the N-3 peak to chemisorbed $NH_3$, $NH_4^+$ species, or oxidized nitrogen species (e.g., $NO_x$)[44-48]. The largest N 1s XPS peak appeared at 400.1 eV, suggesting that nitrogen-containing species preferentially incorporated as interstitial structure, or *$NH_2$ species adsorbed on $CeO_2$ domains, thereby guiding the migration and anchoring of ruthenium onto $CeO_2$ surfaces. Notably, this behavior is distinct from that observed for ruthenium supported on nitrogen-doped carbon, in which nitrogen species predominantly exist as substitutes rather than interstitial nitrogen (Supplementary Fig. 16).

To further resolve the temporal evolution of this process, time-resolved UHR-TEM and N 1s XPS analyses were conducted. Ru/$CeO_2$-C_$N_2$ was exposed to $NH_3$ at 500 °C for varying durations (1, 5, 10, 20, and 40 min), followed by rapid quenching (Supplementary Fig. 18). At early stages (1 and 5 min), Ru species remained broadly distributed, including on carbon domains. A pronounced redistribution occurred between 5 and 10 min, where Ru signals on carbon decreased and became increasingly localized on $CeO_2$ domains. With prolonged treatment, Ru species were predominantly associated with $CeO_2$, indicating structural stabilization. Consistently, time-resolved N 1s XPS results showed that nitrogen species were not detected at early stages but emerged from ~10 min onward, coinciding with the onset of Ru migration (Supplementary Fig. 19). Collectively, these findings demonstrate that nitrogen-containing intermediates generated during $NH_3$ decomposition not only mobilize ruthenium, but also direct its relocation to the $CeO_2$ domains, thereby promoting the formation of Ru-$CeO_2$ interfaces.

To clarify the nature of nitrogen species, N K-edge soft X-ray absorption measurements were performed. As a reference, N-doped $CeO_2$ was synthesized via urea thermolysis under $N_2$, which is known to have dominant interstitial N species[45]. Although N-doped $CeO_2$ exhibited a similar N 1s XPS peak to Ru/$CeO_2$-C_$NH_3$, its N K-edge spectrum showed a $\sigma^*$-dominant feature with negligible $\pi^*$ contribution (Supplementary Fig. 20a). In contrast, Ru/$CeO_2$-C_$NH_3$

showed a markedly different N K-edge spectrum, characterized by a pronounced $\pi^*$ feature at 402.2 eV and a broader, less defined $\sigma^*$ region (Supplementary Fig. 20b)[49-51]. This indicates that, although the N 1s XPS results are similar, the nitrogen species in Ru/$CeO_2$-C_$NH_3$ are not equivalent to interstitial nitrogen, but instead exist in more electronically hybridized environments, likely surface chemisorbed *$NH_2$ on $CeO_2$ domains.

To further examine the evolution of nitrogen species under reaction conditions, the catalyst after 100 h of ammonia decomposition was analyzed. While the N 1s XPS remains centered at 400.1 eV, the N K-edge spectrum showed a clear change, with decreased $\pi^*$ intensity at 402.2 eV and an increased intensity at 399.8 eV (Supplementary Fig. 20c). Although this change primarily occured within the $\pi^*$ region rather than forming a fully $\sigma^*$-dominant spectrum, the overall spectral shape shifted toward that of the interstitial-N-dominant reference material. These results suggest that nitrogen species are initially formed as chemisorbed *$NH_2$, but undergo partial evolution toward a more stabilized, lattice-associated interstitial configuration during reaction.

Finally, density functional theory calculations were performed to elucidate the role of nitrogen-containing intermediates in both the mobilization of Ru species and their subsequent stabilization on $CeO_2$ surfaces. The adsorption energies and the energy required to eject a single ruthenium atom from the Ru(0001) surface in the presence of the adsorbed *$NH_3$, *$NH_2$, *NH, and *H species were calculated (Supplementary Fig. 21 and Supplementary Table 3). Among these, *$NH_2$ was the most effective at promoting ruthenium detachment, corresponding to the highest upshift in the ruthenium d-band center. The resulting Ru-$NH_2$ species were then stabilized on both carbon and $CeO_2$ (111) supercells, with adsorption showing preference for the $CeO_2$ than for carbon (Supplementary Fig. 22). Also, we constructed models of N-doped $CeO_2$ (111) with nitrogen located at interstitial sites and evaluated their stability. Among the considered configurations, surface interstitial (case 1 in Supplementary Fig. 23a) was found to be the most stable (nitrogen binding energy of -2.45 eV), compared to subsurface configurations. Based on this configuration, adsorption energies of Ru on pristine $CeO_2$ and N-doped $CeO_2$ surface were calculated. The adsorption energy of Ru was significantly enhanced from -4.97 eV on pristine $CeO_2$ to -6.58 eV on N-doped $CeO_2$, indicating stronger interaction between Ru and N-doped surface.

**[Revised Supplementary Information, Catalyst Synthesis]**

Nitrogen-doped $CeO_2$ was synthesized following a previously reported method[2]. 200 mg of urea (Sigma-Aldrich) was well mixed with 400 mg of $CeO_2$ (Sigma-Aldrich). Then, this powder was thermally treated in $N_2$ at 400 °C for 4 h.

**[Revised Supplementary Information, Characterization]**

X-ray absorption spectroscopy (XAS) at the Ru K-edge was performed at the BM31 setup of Swiss Norwegian beamlines (SNBL at ESRF) and 7D beamline of the Pohang Light Source (PLS). The Ru K-edge spectra were calibrated against Ru foil, and data processing was carried out using ATHENA and ARTEMIS software packages. N K-edge spectra were obtained in 10D beamline of Pohang Light Source (PLS).

**[Revised Supplementary Information, Computational detail]**

To model the stable N-doped $CeO_2(111)$ system, and to reflect the experimental observation of interstitial nitrogen, three potential sites of N were simulated. Case 1 represented nitrogen atom located at the Ce-Ce-O site on the surface of $CeO_2(111)$ slab, case 2 represented nitrogen atom located at the Ce-O-Ce-O site, and case 3 represented nitrogen atom located at the Ce-Ce-O site on the subsurface of $CeO_2(111)$ slab. Adsorption energy of Ru atom on the surface was calculated on the N-doped $CeO_2(111)$ and pristine $CeO_2(111)$, according to the equation shown below.

$$E_{ads}(Ru) = E(Ru/substrate) - E(substrate) - E(Ru)$$

Where, E(Ru/substrate), E(substrate), and E(Ru) denotes the total energy of a Ru-adsorbed substrate (pristine or N-doped $CeO_2$ (111) slab), the total energy of bare substrate, and the total energy of an isolated single Ru atom at vacuum system.

*7. The experimental conditions of the reaction series experiments (Supplementary Fig. 15b, c) are not clearly described. For example, when measuring the $H_2$ reaction series, how do you keep the total flow rate constant while changing the $H_2$ partial pressure? Is it balanced with Ar? This needs to be written clearly in the methods section or figure legend, otherwise the rigor of the experiment is called into question.*

*The explanation for "mitigated hydrogen poisoning" is mainly based on the fact that the $H_2$ reaction series is more negative. But this is only circumstantial evidence. If in situ DRIFTS can be performed to observe the coverage of adsorbed H species on different catalysts, or $H_2$-TPD can be performed to compare the desorption behavior of $H_2$, it will provide more direct experimental support for this conclusion. Specify the exact conditions for reaction series experiments. It is strongly recommended to supplement experiments such as $H_2$-TPD to strengthen the mechanism of resistance to hydrogen poisoning.*

**Answer)**

We apologize for the lack of clarity in the description of the reaction-series experimental conditions. In the $H_2$ reaction-order experiments, the total flow rate was maintained constant by balancing with Ar. Specifically, while varying the $H_2$ flow rate to change the $H_2$ partial pressure, the Ar flow rate was adjusted accordingly so that the total flow corresponded to a space velocity of 60,000 mL/$g_{cat}$·h, while all other parameters remain unchanged. This clarification has now been added to the Supplementary Information.

**[Revised Supplementary Information, Ammonia decomposition]**

The reaction order experiments were conducted by varying the partial pressures of $H_2$ and $NH_3$ while maintaining a constant total flow rate. The total flow rate was balanced with Ar to keep the overall space velocity constant at 60,000 mL/$g_{cat}$·h. For $H_2$ reaction order experiment, Ru/$CeO_2$-C_$NH_3$ was tested at 450 °C with $H_2$ concentrations set to 10 %, 20 %, 30 %, and 40 %. In the case of Ru/$CeO_2$-C_$H_2$, experiment was conducted at 500 °C. $NH_3$ reaction order experiments were carried out by adjusting the $NH_3$ concentration to 100 %, 60 %, 40 %, and 20 %. Ru/$CeO_2$-C_$NH_3$ was tested at 400 °C, while Ru/$CeO_2$-C_$H_2$ was evaluated at 500 °C.

Regarding the reviewer's suggestion to provide more direct evidence for the mitigation of hydrogen poisoning, we performed additional surface-sensitive experiments. First, in-situ DRIFTS analysis using ammonia was performed to probe surface hydroxyl species under $NH_3$ decomposition conditions. To improve signal/noise ratio, the Ru/$CeO_2$-C_$NH_3$ catalyst was diluted with KBr powder to final catalyst content of 4, 2, 1, and 0.5 wt %, and the mixture was loaded into the DRIFTS cell (Harrick) equipped FT-IR (Thermofischer) with a ZnSe window. For catalyst loadings of 4, 2, and 1 wt % to KBr powder, the infrared signal intensity remained below the minimum peak-to-peak signal amplitude required by the instrument for reliable spectral analysis. Only after dilution to 0.5 wt % with KBr powder could be the minimum peak-to-peak signal criterion be satisfied, and therefore all subsequent DRIFTS measurements were performed using the 0.5 wt % diluted sample.

The sample was initially heated to 400 °C under flowing He, after which $NH_3$ was introduced. After 15 min of $NH_3$ exposure, the gas feed was switched back to He, and the system was purged for an additional 30 min until no further spectral changes were observed, ensuring complete removal of gas-phase $NH_3$. **Fig. R10** shows $NH_3$-DRIFTS results.

Under $NH_3$ exposure, only gas-phase $NH_3$ features were clearly detected (**Fig. R10 left**). After purging, the remaining signals corresponded to strongly adsorbed $NH_3$-derived species, while surface hydroxyl-related features could not be resolved (**Fig. R10 right**). In particular, peaks at 3133, 3037, and 1406 $cm^{-1}$ were observed, which are assigned to $NH_4^+$ species formed by protonation of $NH_3$ at Brønsted acid sites[1-2]. These observations indicate that, although strongly bound gas $NH_3$-derived species can be detected, this in-situ DRIFTS approach was not suitable for directly monitoring surface OH species or hydrogen-related surface coverage in carbon-containing catalyst system.

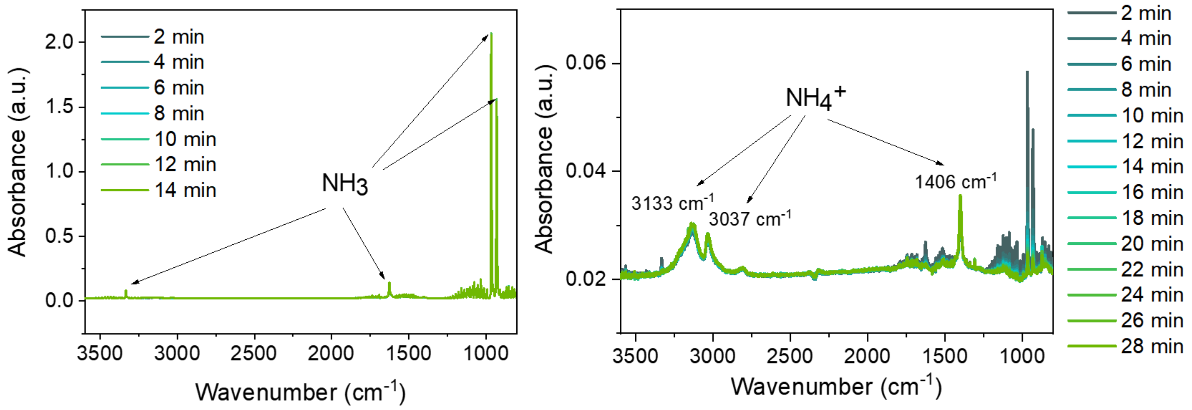


**Fig. R10.** $NH_3$-DRIFTS spectra of Ru/$CeO_2$-C_$NH_3$ collected under flowing $NH_3$ (left) and switching to He purge (right) at 400 °C

To directly investigate hydrogen adsorption behavior, temperature-programmed desorption using $H_2$ ($H_2$-TPD) experiments were subsequently performed (**Fig. R11**). In these experiments, the catalyst was pretreated in flowing Ar at 200 °C, and cooled down to 30 °C under Ar. This was followed by hydrogen adsorption at 30 °C using a 4 vol% $H_2$/Ar mixture and subsequent Ar purging to remove gas-phase and weakly physisorbed hydrogen. During the temperature ramp from 30 to 800 °C under Ar, two distinct desorption regions were observed. The low-temperature desorption feature below approximately 200 °C was attributed to hydrogen desorption from Ru sites, while the broader desorption feature appearing in the 300-600 °C range was associated with hydrogen stored on or within the $CeO_2$ support by $H_2$-spillover[3, 4].

Notably, the Ru/$CeO_2$-C_$NH_3$ catalyst exhibited a pronounced high-temperature $CeO_2$-related desorption peak despite hydrogen adsorption was conducted only at room temperature. This observation indicates that a substantial fraction of hydrogen initially adsorbed on Ru migrated to the $CeO_2$ support via hydrogen spillover. Such efficient hydrogen redistribution away from Ru active sites suggests that hydrogen produced during ammonia decomposition can be accommodated by the support, thereby reducing hydrogen accumulation on Ru and mitigating hydrogen poisoning. This interpretation provides direct experimental support for the observed kinetic behavior. Accordingly, **Fig. R11** was added as **Supplementary Fig. 30**, and manuscript was revised as follows.

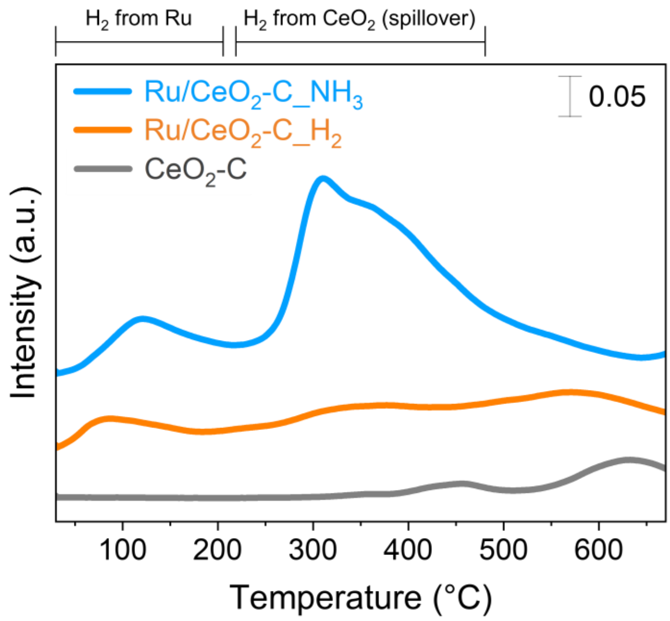


**Fig. R11** $H_2$-TPD results of $CeO_2$-C, Ru/$CeO_2$-C_$H_2$, and Ru/$CeO_2$-C_$NH_3$

**[References]**


1 Yang, X., Zhao, B., Zhuo, Y., Gao, Y., Chen, C., & Xu, X., DRIFTS study of ammonia activation over CaO and sulfated CaO for NO reduction by $NH_3$. *Environmental Science & Technology*, **45**, 1147-1151, (2011).

2 Giordanino, F., Borfecchia, E., Lomachenko, K. A., Lazzarini, A., Agostini, G., Gallo, E., ... & Lamberti, C., Interaction of $NH_3$ with Cu-SSZ-13 catalyst: a complementary FTIR, XANES, and XES study. *The Journal of Physical Chemistry Letters*, **5**, 1552-1559, (2014).

3 Wang, N., Liu, B., Liao, Z., Liu, Q., Lin, X., & Qiu, X., Structurally confined Ni with oxygen-deficient $CeO_2$ for efficient self-transfer hydrogenolysis of lignin into jet fuel precursors. *Small*, **21**, e08631, (2025).

4 Feng, Z., Guo, F., Zhang, Y., Ichikawa, T., & Zheng, J. Oxygen vacancies rich $CeO_2$ supported Ru catalyst for efficient hydrogenation of N-ethylcarbazole at mild temperature. *Applied Catalysis B: Environmental*, **366**, 125059, (2025).


**[Revised manuscript, page 22]**

The formation of basic surface sites due to hydrogen trapping on $CeO_2$ was supported by the results of temperature programmed desorption using $CO_2$ ($CO_2$-TPD). Ru/$CeO_2$-C_$NH_3$ exhibited a desorption peak that shifted toward higher temperatures with a larger peak signal, indicating enhanced basicity of the surface (Fig. 5d). This agrees with the increase in the $Ce^{3+}$ and $O_{ads}$ ratios determined using XPS (Supplementary Fig. 15). Also, to directly investigate hydrogen adsorption behavior, temperature-programmed desorption using $H_2$ ($H_2$-TPD) experiments were performed (Supplementary Fig. 30). A low-temperature desorption peak below ~200 °C is attributed to hydrogen desorption from Ru sites, while a broader feature at

300-600 °C corresponds to hydrogen stored on $CeO_2$ via $H_2$-spillover[64,65]. Notably, Ru/$CeO_2$-C_$NH_3$ exhibited a pronounced high-temperature desorption peak despite hydrogen adsorption at room temperature, indicating efficient hydrogen spillover from Ru to the $CeO_2$ support. This redistribution of hydrogen away from Ru sites reduced hydrogen accumulation and mitigated hydrogen poisoning.

**[Revised Supplementary Information, Characterization]**

Cryo-TPO and $H_2$-TPD were conducted with AutoChem III (Micromeritics) equipped with a thermal conductivity detector (TCD). In case of cryo-TPO, the samples were first mildly reduced at 200 °C under 5% $H_2$/Ar, followed by cooling to -95 °C under He. Subsequently, the gas was switched to 5% $O_2$/He, and after stabilization, the temperature was increased to 400 °C while monitoring oxygen consumption. For $H_2$-TPD, the catalyst was pretreated in flowing Ar at 200 °C, and cooled down to 30 °C under Ar. This was followed by hydrogen adsorption at 30 °C using a 4 vol% $H_2$/Ar mixture and subsequent Ar purging to remove gas-phase and weakly physisorbed hydrogen. Finally, temperature was ramped from 30 to 800 °C under Ar.

*8. $H_2$-TPR (Fig. 2c): Only the peak temperature is discussed, but the quantitative analysis of the peak area is insufficient. The peak area is related to the number of reducible oxygen species. The peak area of Ru/CeC_$NH_3$ is larger. Its association with more interface sites or defect sites should be discussed in depth instead of talking about "strong interactions" in general. Raman spectrum (Figure 2d): The assignment of peaks at ~690 and ~971 cm-1 (Ru-O-Ce) directly quoted from the literature, but did not consider the possible complex signals of the carrier itself (CeC). The Raman spectrum of the CeC_550 support needs to be provided as a baseline to prove that these peaks indeed originate from the introduction of Ru.*

**Answer)**

We thank the reviewer for this important comment. We agree that discussion of the $H_2$-TPR results should not be limited to the peak temperature alone, and that quantitative interpretation of the peak area provides additional insight into the catalyst structure. In the revised manuscript, we therefore expanded the discussion of both the reduction temperature and the peak area. The higher reduction temperature of Ru/$CeO_2$-C_$NH_3$ indicates that the Ru species are more strongly coupled with the $CeO_2$ domains, consistent with the formation of a more intimate Ru-$CeO_2$ interfacial structure. At the same time, the larger reduction peak area suggests that a greater amount of reducible oxygen species is present in this catalyst.

We interpreted this result not only as a manifestation of "strong interaction" in a general sense, but more specifically for larger population of redox-active interfacial sites and/or defect-associated oxygen species in Ru/$CeO_2$-C_$NH_3$ in the revised manuscript. In other words, the ammonia-treated catalyst appears to contain both stronger Ru-$CeO_2$ interaction and a greater amount of oxygen species that can participate in redox processes.

This point is particularly relevant to ammonia decomposition reaction, where the Ru-$CeO_2$ interface is proposed to facilitate hydrogen transfer and alleviate hydrogen poisoning. In this context, the increased amount of reducible oxygen species in Ru/$CeO_2$-C_$NH_3$ may provide a

more favorable redox environment for hydrogen accommodation and removal, thereby contributing to the improved catalytic performance. We have revised the manuscript accordingly to clarify that the larger $H_2$-TPR peak area is associated with more abundant redox-active interfacial/defect sites, rather than referring only broadly to "strong interactions."

**[Revised manuscript, page 9]**

In addition to the XAS results, $H_2$-TPR analyses were performed to gain deeper insight into the surface reducibility of the catalysts and their metal-support interactions (Fig. 2c). Ru/$CeO_2$ exhibited a small reduction peak at 123 °C, while Ru/C exhibited no reduction peak. Ru/$CeO_2$-C_$NH_3$ exhibited a single broad reduction peak at 157 °C, whereas Ru/$CeO_2$-C_$H_2$ exhibited two distinct peaks at 89 and 112 °C. The higher reduction temperature of Ru/$CeO_2$-C_$NH_3$ indicates that Ru forms strong interactions with $CeO_2$, likely as Ru-O-Ce species. In contrast, the peaks at lower temperatures for Ru/$CeO_2$-C_$H_2$ suggest the presence of weakly bound ruthenium nanoparticles or clusters on $CeO_2$. Notably, Ru/$CeO_2$-C_$NH_3$ also exhibited a larger reduction peak area, indicating a greater amount of reducible oxygen species. This feature suggests the presence of a higher density of redox-active interfaces in the catalyst, which are associated with surface defect concentration. Such an increase in reducible oxygen is expected to enhance redox cycling at the Ru-$CeO_2$ interface, potentially facilitating hydrogen adsorption-desorption dynamics and mitigating hydrogen accumulation on Ru sites. To further probe the oxygen-related surface properties, cryo-TPO were conducted (Supplementary Fig. 13). Both catalysts exhibited oxygen uptake starting from -95 °C, indicating that the Ru surface can be readily oxidized even at very low temperatures. Notably, Ru/$CeO_2$-C_$NH_3$ showed a larger oxygen uptake with an additional low-temperature shoulder feature compared to Ru/$CeO_2$-C_$H_2$, suggesting a higher density of oxygen-taking Ru species. This result is consistent with the formation of strongly interacting and highly dispersed Ru species on $CeO_2$, leading to a more oxygen-rich interfacial environment. Overall, the combined $H_2$-TPR and cryo-TPO results support that Ru/$CeO_2$-C_$NH_3$ possesses a higher density of redox-active interfacial oxygen species.

We also thank about the comment regarding the Ru-O-Ce peaks in Raman spectra. To clarify the origin of the Raman features, additional Raman measurements of the $CeO_2$-C supports after different thermal treatments were conducted as a reference (**Fig. R12**). The Raman spectra of $CeO_2$-C didn't exhibit any peaks at ~690 and ~971 $cm^{-1}$. These bands were only observed after the incorporation of Ru, indicating that they were not originated from $CeO_2$-C support. On this basis, the peaks at ~690 and ~971 $cm^{-1}$ are attributed to Ru-O-Ce interfacial species, in agreement with previous reports. The Raman spectra of $CeO_2$-C were included in the **Supplementary Fig. 14** in the revised manuscript.

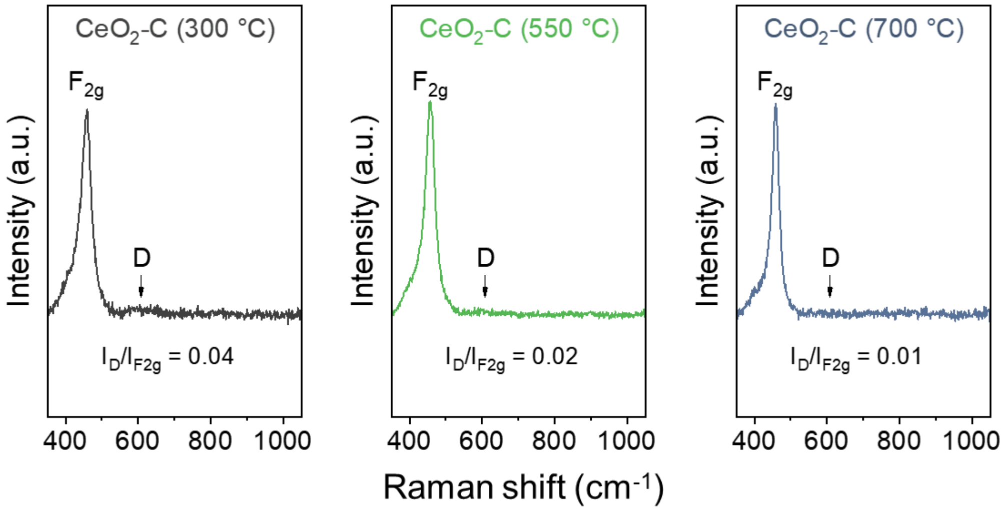


**Fig. R12** Raman spectra results of $CeO_2$-C calcined at 300, 550, and 700 °C under $N_2$.

**[Revised manuscript, page 10]**

Enhanced interaction with Ru and $CeO_2$ was also confirmed with Raman spectra (Fig. 2d). The peak at 465 $cm^{-1}$ in the Raman spectra indicates an octahedrally symmetrical vibration mode of $CeO_2$ ($F_{2g}$), and that at 600 $cm^{-1}$ indicates a defect-induced mode (D). Furthermore, the peaks observed at 690 and 971 $cm^{-1}$ were attributed to Ru-O-Ce (Ru) interactions[38-41]. Those peaks were not observed in Raman spectrum of $CeO_2$-C (Supplementary Fig. 14).

*9. It should be mentioned that some of the conclusions drawn in this paper seem to be a little unconvincing. For example, it is slightly far-fetched to directly infer the migration kinetics of Ru from $CH_4$ generation experiments (Fig. 5a-c). The limitations of this circumstantial evidence need to be expressed more carefully.*

**Answer)**

We thank the reviewer for this important comment regarding the interpretation of the methane formation experiments and their relation to Ru migration. We agree that methane generation alone cannot provide direct information on the migration kinetics of Ru species, and that conclusions based solely on this observation may be overinterpreted. To further support our interpretation, we have incorporated additional time-resolved analyses that provide more direct insight into the structural evolution of Ru species. First, time-resolved TEM observations showed that significant Ru redistribution occurs between 5 and 10 minutes under $NH_3$ treatment, followed by gradual stabilization at longer times. This provides direct visual evidence of the migration process (**Fig. R5**).

Second, time-resolved N 1s XPS measurements revealed that nitrogen species are not detected in the early stages (1 and 5 min), but begin to appear from 10 minutes onward. This indicates that the formation of nitrogen species, which are proposed to interact with and stabilize Ru, occurs on a similar timescale as the observed Ru migration (**Fig. R13**). Importantly, these observations are consistent with the temporal evolution of methane formation (**Fig. R14; Fig. 5a-c in manuscript**). The $CH_4$ signal reaches a maximum at around

5 minutes and decreases significantly thereafter, indicating that the most active restructuring processes occur at early times. The coincidence of this transition with the onset of Ru migration (TEM) and nitrogen incorporation (XPS) suggests a strong correlation between these processes.

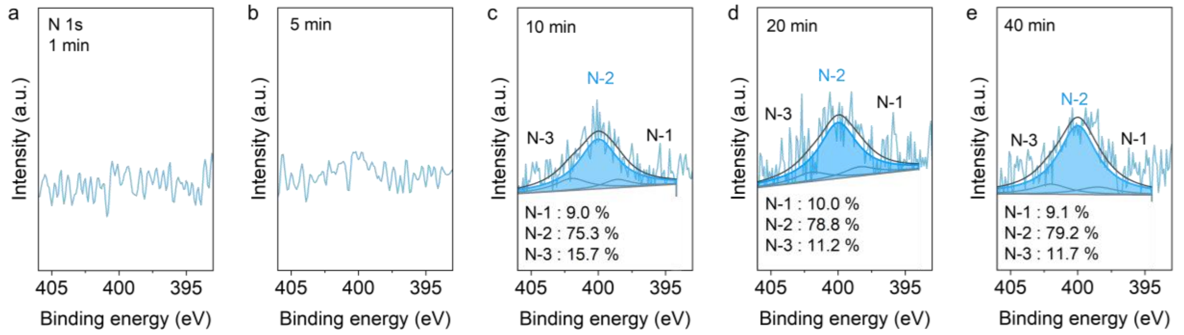


**Fig. R13** Time-resolved N 1s XPS results $NH_3$ treatment on Ru/$CeO_2$-C_$N_2$ for 1 min, 5 min, 10 min, 20 min, and 40 min.

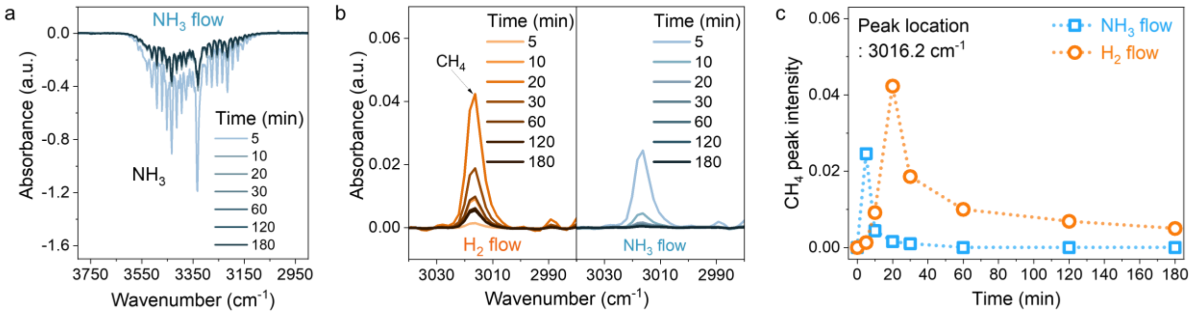


**Fig. R14; Fig. 5** (a) $NH_3$ consumption for Ru/$CeO_2$-C_$N_2$ monitored using FT-IR gas cells at 500 °C for 3 h. (b, c) $CH_4$ formation observed using FT-IR gas cells for Ru/$CeO_2$-C_$N_2$ after treatment with 50% $H_2$/Ar (orange) and 100% $NH_3$ (blue) at 500 °C for 3 h.

Taken together, while methane evolution alone does not directly quantify migration kinetics, the combined time-resolved TEM, XPS, and gas analysis results provide consistent and mutually supportive evidence for the dynamic restructuring of Ru species during $NH_3$ treatment. Accordingly, we have revised the manuscript to clarify that methane formation should be interpreted as supporting, rather than definitive, evidence of Ru migration.

**[Revised manuscript, page 21]**

This behavior was first confirmed by $CH_4$ detection using Fourier-transform infrared spectroscopy with a gas-cell setup. Treating Ru/$CeO_2$-C_$N_2$ with pure $NH_3$ at 500 °C resulted in rapid consumption of $NH_3$ within 10 min, which stabilized for 3 h (Fig. 5a). Simultaneously, $CH_4$ was produced, but diminished quickly and disappeared after approximately 20 min, indicating the rapid rearrangement of ruthenium onto the $CeO_2$ surface. This $CH_4$ formation result was consistent with time-resolved TEM and time-resolved N 1s XPS results (Supplementary Fig. 18 and 19), which showed that Ru migration occurs within a similar time window accompanied by the formation of nitrogen species. The coincidence of methane

formation with the Ru migration onset and nitrogen incorporation suggests a strong correlation between these results. In contrast, the $H_2$-treatment at 500 °C on Ru/$CeO_2$-C_$N_2$ exhibited continuous $CH_4$ formation over 3 h with a larger total amount, suggesting the prolonged residence of ruthenium on the carbon support (Fig. 5b, c).

*10. It is observed that an apparent discrepancy in Figure 4c, where the ammonia decomposition rates at 450 °C appear to be higher than those at 500 °C for the catalyst, which is not match the ammonia decomposition performance test mentioned in Fig 4a. This seems counterintuitive, as reaction rates typically increase with temperature. Could the authors please verify if the temperature labels for the long-term stability tests are correct?*

**Answer)**

We thank the reviewer for carefully examining the data and for raising this point. We have rechecked the temperature labels and confirm that they are correctly assigned in Fig. 4c. The apparent discrepancy arises from differences in catalytic performance between the samples rather than an inconsistency in temperature labeling. As shown in Fig. 4a, the Ru/$CeO_2$-C_$NH_3$ catalyst exhibits significantly higher activity than Ru/$CeO_2$-C_$H_2$ across the investigated temperature range. In particular, the activity of Ru/$CeO_2$-C_$NH_3$ at 450 °C is higher than that of Ru/$CeO_2$-C_$H_2$ even at 500 °C.

In addition, we would like to clarify that the long-term durability tests were conducted at different temperatures for each catalyst in order to maintain a comparable ammonia conversion range (in between 30-60%). Therefore, the reaction temperatures were selected individually based on the intrinsic activity of each catalyst. We acknowledge that this experimental detail may not have been sufficiently clear in the original figure presentation. To improve clarity, Fig. 4 has been revised to more explicitly indicate the corresponding reaction temperatures for each dataset. Taken together, the observed trend in Fig. 4c is consistent with the intrinsic catalytic performance differences between the samples, and does not originate from an error in temperature labeling.

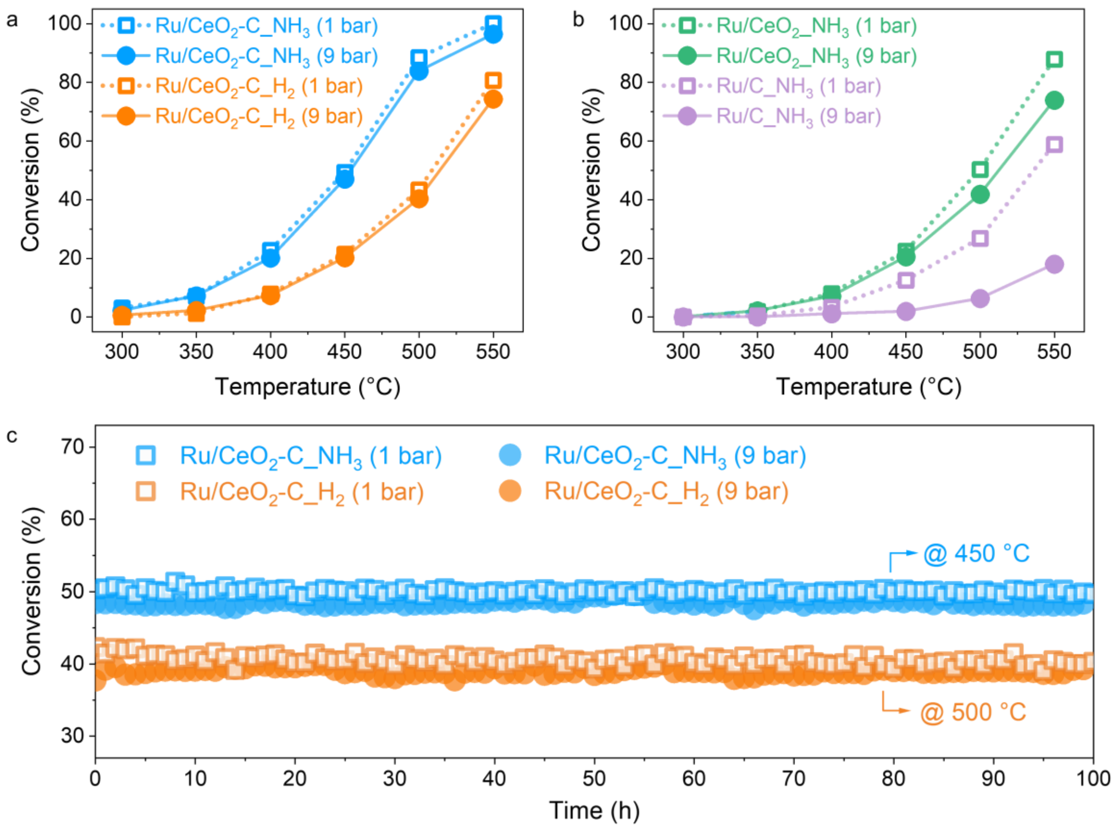


**Fig. 4: Catalytic performance in ammonia decomposition reactions** $NH_3$ conversion results for (a) Ru/$CeO_2$-C_$NH_3$, Ru/$CeO_2$-C_$H_2$, (b) Ru/$CeO_2$, and Ru/C at 1 and 9 bar. (c) Long-term stability test of Ru/$CeO_2$-C_$NH_3$ and Ru/$CeO_2$-C_$H_2$ for 100 h at 1 and 9 bar. The long-term reaction was conducted at 450 and 500 °C for Ru/$CeO_2$-C_$NH_3$ and Ru/$CeO_2$-C_$H_2$, respectively. $NH_3$ decomposition was conducted using 100% $NH_3$ with a WHSV of 30,000 mL/$g_{cat}$·h.

*11. Grammatical errors and unclear expressions should be carefully checked and corrected.*

**Answer)**

We thank the reviewer for this comment. The manuscript had been proofread by a professional English editing service prior to the initial submission. In response to this comment, the manuscript has been carefully proofread throughout, with particular attention to grammatical accuracy and clarity of expression. All identified grammatical errors have been corrected, and sentences that may have been ambiguous or syntactically awkward have been revised to improve readability.
